# Supplementary material for: Comparing patients’ and other stakeholders’ preferences for outcomes of integrated care for multimorbidity: a discrete choice experiment in eight European countries
Source: BMJ Open. 2020 Oct 10;10(10):e037547. doi: 10.1136/bmjopen-2020-037547 (PMC7552858; doi:10.1136/bmjopen-2020-037547)
Supplement: Supplementary data [file bmjopen-2020-037547supp001.pdf]

Online supplementary file

Box S1. Original, literature-based, priors.

	DCE-Level		
**DCE-Attribute**	**Worst (1)**	**Average (2) Mean (SD)**	**Best (3) Mean (SD)**
Physical functioning	Reference	0.30 (0.12)	0.60 (0.23)
Psychological wellbeing	Reference	0.20 (0.08)	0.40 (0.16)
Social relations & participation	Reference	0.25 (0.10)	0.45 (0.18)
Enjoyment of life	Reference	0.20 (0.08)	0.40 (0.16)
Resilience	Reference	0.20 (0.08)	0.40 (0.16)
Person-centeredness	Reference	0.10 (0.04)	0.25 (0.10)
Continuity of care	Reference	0.15 (0.06)	0.30 (0.12)
Total costs	Reference	0.15 (0.06)	0.35 (0.14)

Rutten-van Mölken M, *et al. BMJ Open* 2020; 10:e037547. doi: 10.1136/bmjopen-2020-037547

Box S2. Example of OpenBUGS model code for the S-MNL models.

All models were programmed in the BUGS language and estimated using OpenBUGS using Bayesian Markov Chain Monte Carlo (MCMC) methods. This entails selecting prior distributions for the unknown model parameters and updating these via the likelihood of the observed choice data. The specification of the priors and the model codes are presented below. All estimations used 10,000 MCMC iterations to let three chains converge from divergent starting points and another 20,000 MCMC iterations to reliably approximate the posterior distributions of the parameters. Convergence was evaluated based on a visual inspection of the MCMC chains and the diagnostics as included in the OpenBUGS software. Custom MCMC slice samplers were used for the GMNL-scale and the rho parameters; these are available from the authors upon request.

Model #1. Separate S-MNL models for each stakeholder group

```
model {

 # N  = number of respondents
 # N1 = number of respondents in P1 group
 # N2 = number of respondents in P2 group
 # N3 = number of respondents in P3 group
 # N4 = number of respondents in P4 group
 # T  = number of choice tasks
 # V  = number of parameters

 # likelihood of pairwise choice tasks
 for (n in 1:N){
   for(t in 1:T){
     Y[n,t] <- 0
     Y[n,t] ~ dcat2(phi[n,t])
 }}

 # utility function P1
 for (n in 1:N1){
   for (t in 1:T){
     phi[n,t]  <- (attr1[n,t] + attr2[n,t] + attr3[n,t] + attr4[n,t] + attr5[n,t] +
                   attr6[n,t] + attr7[n,t] + attr8[n,t]) * gmnl_scale[n]
     attr1[n,t] <- (beta[1,1]  * X[n,t,1]  + beta[1,2]  * X[n,t,2] )
     attr2[n,t] <- (beta[1,3]  * X[n,t,3]  + beta[1,4]  * X[n,t,4] )
     attr3[n,t] <- (beta[1,5]  * X[n,t,5]  + beta[1,6]  * X[n,t,6] )
     attr4[n,t] <- (beta[1,7]  * X[n,t,7]  + beta[1,8]  * X[n,t,8] )
     attr5[n,t] <- (beta[1,9]  * X[n,t,9]  + beta[1,10] * X[n,t,10])
     attr6[n,t] <- (beta[1,11] * X[n,t,11] + beta[1,12] * X[n,t,12])
     attr7[n,t] <- (beta[1,13] * X[n,t,13] + beta[1,14] * X[n,t,14])
     attr8[n,t] <- (beta[1,15] * X[n,t,15] + beta[1,16] * X[n,t,16])
 }}

 # utility function P2
 for (n in N1+1:N1+N2){
   for (t in 1:T){
     phi[n,t]  <- (attr1[n,t] + attr2[n,t] + attr3[n,t] + attr4[n,t] + attr5[n,t] +
                   attr6[n,t] + attr7[n,t] + attr8[n,t]) * gmnl_scale[n]
     attr1[n,t] <- (beta[2,1] * X[n,t,1]  + beta[2,2] * X[n,t,2] )
     attr2[n,t] <- (beta[2,3] * X[n,t,3]  + beta[2,4] * X[n,t,4] )
     attr3[n,t] <- (beta[2,5] * X[n,t,5]  + beta[2,6] * X[n,t,6] )
     attr4[n,t] <- (beta[2,7] * X[n,t,7]  + beta[2,8] * X[n,t,8] )
     attr5[n,t] <- (beta[2,9] * X[n,t,9]  + beta[2,10]* X[n,t,10])
     attr6[n,t] <- (beta[2,11]* X[n,t,11] + beta[2,12]* X[n,t,12])
     attr7[n,t] <- (beta[2,13]* X[n,t,13] + beta[2,14]* X[n,t,14])
     attr8[n,t] <- (beta[2,15]* X[n,t,15] + beta[2,16]* X[n,t,16])
 }}


 # utility function P3
 for (n in N1+N2+1:N1+N2+N3){
   for (t in 1:T){
     phi[n,t]  <- (attr1[n,t] + attr2[n,t] + attr3[n,t] + attr4[n,t] + attr5[n,t] +
                   attr6[n,t] + attr7[n,t] + attr8[n,t]) * gmnl_scale[n]
     attr1[n,t] <- (beta[3,1] * X[n,t,1]  + beta[3,2] * X[n,t,2] )
     attr2[n,t] <- (beta[3,3] * X[n,t,3]  + beta[3,4] * X[n,t,4] )
     attr3[n,t] <- (beta[3,5] * X[n,t,5]  + beta[3,6] * X[n,t,6] )
     attr4[n,t] <- (beta[3,7] * X[n,t,7]  + beta[3,8] * X[n,t,8] )
     attr5[n,t] <- (beta[3,9] * X[n,t,9]  + beta[3,10]* X[n,t,10])
```


Rutten-van Mölken M, *et al. BMJ Open* 2020; 10:e037547. doi: 10.1136/bmjopen-2020-037547

```
    attr6[n,t] <- (beta[3,11]* X[n,t,11] + beta[3,12]* X[n,t,12])
    attr7[n,t] <- (beta[3,13]* X[n,t,13] + beta[3,14]* X[n,t,14])
    attr8[n,t] <- (beta[3,15]* X[n,t,15] + beta[3,16]* X[n,t,16])
}}

# utility function P4
for (n in N1+N2+N3+1:N1+N2+N3+N4){
  for (t in 1:T){
    phi[n,t]  <- (attr1[n,t] + attr2[n,t] + attr3[n,t] + attr4[n,t] + attr5[n,t] +
                   attr6[n,t] + attr7[n,t] + attr8[n,t]) * gmnl_scale[n]
    attr1[n,t] <- (beta[4,1] * X[n,t,1]  + beta[4,2] * X[n,t,2] )
    attr2[n,t] <- (beta[4,3] * X[n,t,3]  + beta[4,4] * X[n,t,4] )
    attr3[n,t] <- (beta[4,5] * X[n,t,5]  + beta[4,6] * X[n,t,6] )
    attr4[n,t] <- (beta[4,7] * X[n,t,7]  + beta[4,8] * X[n,t,8] )
    attr5[n,t] <- (beta[4,9] * X[n,t,9]  + beta[4,10]* X[n,t,10])
    attr6[n,t] <- (beta[4,11]* X[n,t,11] + beta[4,12]* X[n,t,12])
    attr7[n,t] <- (beta[4,13]* X[n,t,13] + beta[4,14]* X[n,t,14])
    attr8[n,t] <- (beta[4,15]* X[n,t,15] + beta[4,16]* X[n,t,16])
}}

# priors on beta's
beta[1,1:V] ~ dmnorm(zeros[], tau_beta[,])
beta[2,1:V] ~ dmnorm(zeros[], tau_beta[,])
beta[3,1:V] ~ dmnorm(zeros[], tau_beta[,])
beta[4,1:V] ~ dmnorm(zeros[], tau_beta[,])

for (b in 1:V){
 zeros[b] <- 0
 for (bb in 1:V){
   tau_beta[b,bb] <- equals(b,bb)/100
}}

# priors on gmnl_scale parameters
# subject to a mean-of-one constraint for identification
gmnl_scale[1:N1] ~ dGMNLscale(tau[1])
gmnl_scale[N1+1:N1+N2] ~ dGMNLscale(tau[2])
gmnl_scale[N1+N2+1:N1+N2+N3] ~ dGMNLscale(tau[3])
gmnl_scale[N1+N2+N3+1:N1+N2+N3+N4] ~ dGMNLscale(tau[4])
# this implies that SD(tau) is restricted between 0.01 and 10
for (group in 1:4){
  tau[group] ~ dunif(0.01,10000)
}

# monitor SD of gmnl_scale parameters
for (group in 1:4){
  gmnl_SD[group] <- sqrt( 1.0/ tau[group] )
}

# monitor attribute importances
for (group in 1:4){
  weight[group,1] <- beta[group,2]
  weight[group,2] <- beta[group,4]
  weight[group,3] <- beta[group,6]
  weight[group,4] <- beta[group,8]
  weight[group,5] <- beta[group,10]
  weight[group,6] <- beta[group,12]
  weight[group,7] <- beta[group,14]
  weight[group,8] <- beta[group,16]
  total_weight[group] <- sum(weight[group,])
  attr_importance[group,1] <- weight[group,1] / total_weight[group]
  attr_importance[group,2] <- weight[group,2] / total_weight[group]
  attr_importance[group,3] <- weight[group,3] / total_weight[group]
  attr_importance[group,4] <- weight[group,4] / total_weight[group]
  attr_importance[group,5] <- weight[group,5] / total_weight[group]
  attr_importance[group,6] <- weight[group,6] / total_weight[group]
  attr_importance[group,7] <- weight[group,7] / total_weight[group]
  attr_importance[group,8] <- weight[group,8] / total_weight[group]
}
}
```


Rutten-van Mölken M, *et al. BMJ Open* 2020; 10:e037547. doi: 10.1136/bmjopen-2020-037547

Model #2. Combined S-MNL model with multiplicative attribute-importance parameters

```
model {

 # likelihood of pairwise choice tasks
 for (n in 1:N){
   for(t in 1:T){
     Y[n,t] <- 0
     Y[n,t] ~ dcat2(phi[n,t])
 }}

 # utility function P1
 for (n in 1:N1){
   for (t in 1:T){
     phi[n,t]  <- (attr1[n,t] + attr2[n,t] + attr3[n,t] + attr4[n,t] + attr5[n,t] +
                    attr6[n,t] + attr7[n,t] + attr8[n,t]) * gmnl_scale[n] * scale[1]
     attr1[n,t] <- (beta[1]  * X[n,t,1]  + beta[2]  * X[n,t,2] )
     attr2[n,t] <- (beta[3]  * X[n,t,3]  + beta[4]  * X[n,t,4] )
     attr3[n,t] <- (beta[5]  * X[n,t,5]  + beta[6]  * X[n,t,6] )
     attr4[n,t] <- (beta[7]  * X[n,t,7]  + beta[8]  * X[n,t,8] )
     attr5[n,t] <- (beta[9]  * X[n,t,9]  + beta[10] * X[n,t,10])
     attr6[n,t] <- (beta[11] * X[n,t,11] + beta[12] * X[n,t,12])
     attr7[n,t] <- (beta[13] * X[n,t,13] + beta[14] * X[n,t,14])
     attr8[n,t] <- (beta[15] * X[n,t,15] + beta[16] * X[n,t,16])
 }}

 # utility function P2
 for (n in N1+1:N1+N2){
   for (t in 1:T){
     phi[n,t]  <- (attr1[n,t] + attr2[n,t] + attr3[n,t] + attr4[n,t] + attr5[n,t] +
                    attr6[n,t] + attr7[n,t] + attr8[n,t]) * gmnl_scale[n] * scale[2]
     attr1[n,t] <- (beta[1] * X[n,t,1]  + beta[2] * X[n,t,2] ) * rho[2,1]
     attr2[n,t] <- (beta[3] * X[n,t,3]  + beta[4] * X[n,t,4] ) * rho[2,2]
     attr3[n,t] <- (beta[5] * X[n,t,5]  + beta[6] * X[n,t,6] ) * rho[2,3]
     attr4[n,t] <- (beta[7] * X[n,t,7]  + beta[8] * X[n,t,8] ) * rho[2,4]
     attr5[n,t] <- (beta[9] * X[n,t,9]  + beta[10]* X[n,t,10]) * rho[2,5]
     attr6[n,t] <- (beta[11]* X[n,t,11] + beta[12]* X[n,t,12]) * rho[2,6]
     attr7[n,t] <- (beta[13]* X[n,t,13] + beta[14]* X[n,t,14]) * rho[2,7]
     attr8[n,t] <- (beta[15]* X[n,t,15] + beta[16]* X[n,t,16]) * rho[2,8]
 }}


 # utility function P3
 for (n in N1+N2+1:N1+N2+N3){
   for (t in 1:T){
     phi[n,t]  <- (attr1[n,t] + attr2[n,t] + attr3[n,t] + attr4[n,t] + attr5[n,t] +
                    attr6[n,t] + attr7[n,t] + attr8[n,t]) * gmnl_scale[n] * scale[3]
     attr1[n,t] <- (beta[1] * X[n,t,1]  + beta[2] * X[n,t,2] ) * rho[3,1]
     attr2[n,t] <- (beta[3] * X[n,t,3]  + beta[4] * X[n,t,4] ) * rho[3,2]
     attr3[n,t] <- (beta[5] * X[n,t,5]  + beta[6] * X[n,t,6] ) * rho[3,3]
     attr4[n,t] <- (beta[7] * X[n,t,7]  + beta[8] * X[n,t,8] ) * rho[3,4]
     attr5[n,t] <- (beta[9] * X[n,t,9]  + beta[10]* X[n,t,10]) * rho[3,5]
     attr6[n,t] <- (beta[11]* X[n,t,11] + beta[12]* X[n,t,12]) * rho[3,6]
     attr7[n,t] <- (beta[13]* X[n,t,13] + beta[14]* X[n,t,14]) * rho[3,7]
     attr8[n,t] <- (beta[15]* X[n,t,15] + beta[16]* X[n,t,16]) * rho[3,8]
 }}

 # utility function P4
 for (n in N1+N2+N3+1:N1+N2+N3+N4){
   for (t in 1:T){
     phi[n,t]  <- (attr1[n,t] + attr2[n,t] + attr3[n,t] + attr4[n,t] + attr5[n,t] +
                    attr6[n,t] + attr7[n,t] + attr8[n,t]) * gmnl_scale[n] * scale[4]
     attr1[n,t] <- (beta[1] * X[n,t,1]  + beta[2] * X[n,t,2] ) * rho[4,1]
     attr2[n,t] <- (beta[3] * X[n,t,3]  + beta[4] * X[n,t,4] ) * rho[4,2]
     attr3[n,t] <- (beta[5] * X[n,t,5]  + beta[6] * X[n,t,6] ) * rho[4,3]
     attr4[n,t] <- (beta[7] * X[n,t,7]  + beta[8] * X[n,t,8] ) * rho[4,4]
     attr5[n,t] <- (beta[9] * X[n,t,9]  + beta[10]* X[n,t,10]) * rho[4,5]
     attr6[n,t] <- (beta[11]* X[n,t,11] + beta[12]* X[n,t,12]) * rho[4,6]
     attr7[n,t] <- (beta[13]* X[n,t,13] + beta[14]* X[n,t,14]) * rho[4,7]
     attr8[n,t] <- (beta[15]* X[n,t,15] + beta[16]* X[n,t,16]) * rho[4,8]
 }}

 # priors on beta's
 beta[1:V] ~ dmnorm(zeros[], tau_beta[,])

 for (b in 1:V){
  zeros[b] <- 0
  for (bb in 1:V){
    tau_beta[b,bb] <- equals(b,bb)/100
 }}
```

```
 # priors on gmnl_scale parameters
 # subject to a mean-of-one constraint for identification
 gmnl_scale[1:N1] ~ dGMNLscale(tau[1])
 gmnl_scale[N1+1:N1+N2] ~ dGMNLscale(tau[2])
 gmnl_scale[N1+N2+1:N1+N2+N3] ~ dGMNLscale(tau[3])
 gmnl_scale[N1+N2+N3+1:N1+N2+N3+N4] ~ dGMNLscale(tau[4])
 # this implies that SD(tau) is restricted between 0.01 and 10
 for (group in 1:4){
   tau[group] ~ dunif(0.01,10000)
 }

 # monitor SD of gmnl_scale parameters
 for (group in 1:4){
   gmnl_SD[group] <- sqrt( 1.0/ tau[group] )
 }

 # priors on relative attribute importance parameters (P1 is the base-case sample)
 # subject to a mean-of-one constraint for identification
 # see Jonker et al. 2019, this prior places 95% of the prior mass between 0.4 and 2.0
  for (att in 1:8){
   rho[1,att] <- 1
 }
 for (group in 2:4){
   rho[group,1:8] ~ dscale(6.25)
 }

 # priors on the relative scale parameters (P1 is the base-case sample)
 # see Jonker et al. 2019, this prior places 95% of the prior mass between 0.4 and 2.0
 scale[1] <- 1
 for (group in 2:4) {
   scale[group] ~ dlnorm(-0.08,6.25)C(0.1,10)
 }
 # the truncation normalizing constant in the log prior does not need to be explicitly
 # evaluated since the truncation boundaries remain constant during MCMC sampling. Hence
 # sensoring C() is sufficient and can be used instead of truncation T(), which isn't implemented


 # monitor attribute importances
 for (group in 1:4){
   weight[group,1] <- beta[2]  * rho[group,1]
   weight[group,2] <- beta[4]  * rho[group,2]
   weight[group,3] <- beta[6]  * rho[group,3]
   weight[group,4] <- beta[8]  * rho[group,4]
   weight[group,5] <- beta[10] * rho[group,5]
   weight[group,6] <- beta[12] * rho[group,6]
   weight[group,7] <- beta[14] * rho[group,7]
   weight[group,8] <- beta[16] * rho[group,8]
   total_weight[group] <- sum(weight[group,])
   attr_importance[group,1] <- weight[group,1] / total_weight[group]
   attr_importance[group,2] <- weight[group,2] / total_weight[group]
   attr_importance[group,3] <- weight[group,3] / total_weight[group]
   attr_importance[group,4] <- weight[group,4] / total_weight[group]
   attr_importance[group,5] <- weight[group,5] / total_weight[group]
   attr_importance[group,6] <- weight[group,6] / total_weight[group]
   attr_importance[group,7] <- weight[group,7] / total_weight[group]
   attr_importance[group,8] <- weight[group,8] / total_weight[group]
 }
}
```

Box S3. Indicators of questionnaire completion and perceived difficulty

**Completion**	**AT**	**HR**	**HU**	**DE**	**NL**	**NO**	**ES**	**UK**
Gave informed consent	253	229	283	207	207	203	150	208
Finished all DCE scenario's (%)	170 (67)	173 (76)	194 (93)	166 (80)	160 (77)	158 (78)	150 (100)	165 (79)
Finished entire questionnaire	169 (68)	168 (73)	192 (68)	160 (77)	156 (75)	156 (77)	150 (100)	163 (78)
Mean (SD) time to completion entire questionnaire	22.87 (21.73)	22.43 (37.68)	20.27 (14.88)	26.21 (48.74)	26.21 (48.74)	27.33 (27.90)	26.96 (10.12)	24.68 (62.64)
Required >5 minutes (%)*	167 (99)	160 (95)	180 (94)	158 (99)	156 (100)	152 (97)	149 (99)	155 (95)
**Self-reported difficulty**								
Very easy	16 (9.5)	11 (6.5)	23 (12.0)	14 (8.8)	3 (1.9)	2 (1.3)	1 (0.7)	31 (19.0)
Easy	39 (23.1)	30 (17.9)	71 (37.0)	33 (20.6)	40 (25.6)	20 (12.8)	36 (24.0)	45 (27.6)
Not too easy, not too difficult	85 (50.3)	70 (41.7)	75 (39.1)	84 (52.5)	74 (47.4)	79 (50.6)	71 (47.3)	63 (38.7)
Difficult	25 (14.8)	44 (26.2)	22 (11.5)	24 (15.0)	31 (19.9)	48 (30.8)	30 (20.0)	22 (13.5)
Very difficult	4 (2.4)	13 (7.7)	1 (0.5)	5 (3.1)	8 (5.1)	7 (4.5)	12 (8.0)	2 (1.2)

* As % of those that completed the entire questionnaire

Rutten-van Mölken M, *et al. BMJ Open* 2020; 10:e037547. doi: 10.1136/bmjopen-2020-037547

Box S4. Patients' self-reported health problems.

N (%)	AT	HR	HU	DE	NL	NO	ES	UK
I prefer not to answer	4 (2.4)	10 (6.0)	3 (1.6)	4 (1.9)	2 (1.3)	0 (0)	6 (4)	3 (1.8)
Asthma	20 (11.8)	19 (11.3)	26 (13.5)	18 (8.7)	19 (12.2)	29 (18.6)	6 (4)	41 (25.2)
Cancer during the past five years	10 (5.9)	5 (3.0)	11 (5.7)	4 (1.9)	10 (6.4)	11 (7.1)	18 (12)	10 (6.1)
Chronic back pain or sciatica	69 (40.8)	54 (32.1)	32 (16.7)	47 (22.6)	32 (20.5)	29 (18.6)	50 (33.3)	51 (31.3)
Chronic bronchitis, COPD or emphysema	32 (18.9)	8 (4.8)	9 (4.7)	14 (6.7)	26 (16.7)	7 (4.5)	29 (19.3)	28 (17.2)
Chronic kidney disease	4 (2.4)	6 (3.6)	8 (4.2)	9 (4.3)	4 (2.6)	0 (0)	9 (6)	8 (4.9)
Cognitive problems, memory loss, dementia, Alzheimer's	3 (1.8)	0 (0)	2 (1.0)	2 (1.0)	6 (3.8)	6 (3.9)	0 (0)	4 (2.5)
Colon problem, irritable bowel or colitis	28 (16.6)	18 (10.7)	15 (7.8)	9 (4.3)	34 (21.8)	31 (19.9)	7 (4.7)	32 (19.6)
Congestive heart failure	9 (5.3)	1 (0.6)	4 (2.2)	8 (3.8)	9 (5.8)	5 (3.2)	9 (6)	3 (1.8)
Depression, anxiety or emotional difficulties	63 (37.3)	47 (28.0)	63 (32.8)	36 (17.3)	29 (18.6)	32 (20.5)	36 (24)	70 (42.9)
Diabetes	29 (17.2)	17 (10.1)	43 (22.4)	35 (16.8)	36 (23.1)	16 (10.3)	26 (17.3)	45 (27.6)
Hard of hearing	18 (10.7)	16 (9.5)	27 (14.1)	14 (6.7)	28 (17.9)	35 (22.4)	17 (11.3)	18 (11.0)
Heart disease, angina (chest pain from heart problem), heart attack, bypass surgery or angioplasty	20 (11.8)	19 (11.3)	20 (10.4)	10 (4.8)	26 (16.7)	15 (9.6)	19 (12.7)	28 (17.2)
High blood pressure	67 (39.6)	40 (23.8)	95 (49.5)	68 (32.7)	61 (39.1)	58 (37.2)	50 (33.3)	64 (39.3)
High cholesterol	34 (20.1)	27 (16.1)	46 (24.0)	40 (19.2)	47 (30.1)	38 (24.4)	45 (30)	47 (28.8)
Osteoarthritis (not rheumatoid arthritis)	5 (3.0)	9 (5.4)	59 (30.7)	7 (3.4)	33 (21.2)	46 (29.5)	7 (4.7)	43 (26.4)
Osteoporosis (brittle bones)	15 (8.9)	11 (6.5)	13 (6.8)	6 (2.9)	10 (6.4)	9 (5.8)	21 (14)	16 (9.8)
Overweight	55 (32.5)	46 (27.4)	68 (35.4)	56 (26.9)	49 (31.4)	51 (32.7)	47 (31.3)	47 (28.8)
Poor circulation in your legs	22 (13.0)	27 (16.1)	49 (25.5)	17 (8.2)	21 (13.5)	22 (14.1)	35 (23.3)	23 (14.1)
Rheumatoid arthritis	25 (14.8)	10 (6.0)	19 (9.9)	17 (8.2)	21 (13.5)	10 (6.4)	12 (8)	19 (11.7)

Rheumatic disease, fibromyalgia or lupus	14 (8.3)	5 (3.0)	13 (6.8)	15 (7.2)	11 (7.1)	16 (10.3)	12 (8)	11 (6.7)
Stomach problem, ulcer, gastritis or reflux	29 (17.2)	27 (16.1)	46 (24.0)	25 (12.0)	21 (13.5)	24 (15.4)	22 (14.7)	28 (17.2)
Stroke	4 (2.4)	4 (2.4)	8 (4.2)	10 (4.8)	3 (1.9)	2 (1.3)	7 (4.7)	9 (5.5)
Thyroid disorder	30 (17.8)	33 (19.6)	23 (12.0)	27 (13.0)	14 (9.0)	18 (11.5)	10 (6.7)	16 (9.8)
Vision problem	26 (15.4)	50 (29.8)	28 (14.6)	29 (13.9)	28 (17.9)	21 (13.5)	56 (37.3)	18 (11.0)
No health problems	1 (0.6)	6 (3.6)	0 (0.0)	28 (13.5)	2 (1.3)	0 (0)	3 (2)	0 (0)
Other health problems	39 (23.1)	30 (17.9)	31 (16.2)	16 (7.7)	39 (25.0)	34 (21.8)	31 (20.7)	73 (44.8)

Supplementary file Box S5. Coefficients and 95% credibility intervals (CI) of the multiplicative interaction terms to investigate differences between patients (P1) and the other stakeholder groups, i.e., Partners & other informal caregivers (P2), professionals (P3), payers (P4), and policy makers (P5). The bold cells indicate the 95% CI of the multiplicative interaction terms for stakeholder group and attribute that excluded 1.

		**P2 vs P1**			**P3 vs P1**			**P4/5 vs P1**			**P4 vs P1**			**P5 vs P1**		
		**coefficient**	**95% CI**		**coefficient**	**95% CI**		**coefficient**	**95% CI**		**coefficient**	**95% CI**		**coefficient**	**95% CI**	
**AT**	Physical functioning	0.74	0.52	**1.00**	0.64	0.48	**0.82**	0.65	0.48	**0.87**						
	Psych.l well-being	1.25	0.95	1.62	1.14	0.91	1.42	1.06	0.82	1.35						
	Social relations & part	0.93	0.62	1.29	1.16	0.92	1.45	1.09	0.83	1.40						
	Enjoyment of life	1.11	0.88	1.38	0.94	0.78	1.11	0.89	0.72	1.09						
	Resilience	0.98	0.74	1.24	0.87	0.70	1.07	0.79	0.61	1.00						
	Person-centeredness	1.27	0.79	1.83	1.45	**1.07**	1.88	1.16	0.79	1.58						
	Continuity of care	1.01	0.76	1.30	1.17	0.94	1.41	1.07	0.83	1.34						
	Total costs	0.71	0.40	1.10	0.64	0.39	**0.97**	1.29	0.87	1.83						
**DE**	Physical functioning	0.56	0.34	**0.83**	0.52	0.38	**0.69**	0.77	0.57	1.00						
	Psychological well-being	0.81	0.50	1.22	1.04	0.77	1.40	1.19	0.88	1.58						
	Social relations & part	0.98	0.63	1.40	1.01	0.77	1.31	0.88	0.64	1.16						
	Enjoyment of life	1.07	0.84	1.35	0.80	0.66	**0.96**	0.78	0.63	**0.96**						
	Resilience	1.00	0.74	1.29	0.78	0.63	**0.96**	0.73	0.57	**0.91**						
	Person-centeredness	1.09	0.66	1.64	1.70	**1.28**	2.17	1.17	0.82	1.59						
	Continuity of care	1.32	1.00	1.69	1.19	0.98	1.44	0.89	0.69	1.12						
	Total costs	1.16	0.66	1.72	0.95	0.65	1.36	1.59	**1.15**	2.15						
**ES**	Physical functioning	1.23	**1.06**	1.44	0.51	0.41	**0.64**									
	Psychological well-being	0.77	0.64	**0.91**	0.73	0.60	**0.87**									
	Social relations & part	1.01	0.84	1.20	0.73	0.60	**0.88**									
	Enjoyment of life	0.87	0.72	1.03	0.96	0.80	1.13									
	Resilience	1.01	0.83	1.22	0.80	0.65	**0.99**									
	Person-centeredness	1.43	**1.16**	1.75	1.64	**1.35**	1.96									
	Continuity of care	0.95	0.70	1.25	1.12	0.86	1.45									
	Total costs	0.73	0.43	1.11	1.50	**1.06**	2.09									

Rutten-van Mölken M, *et al. BMJ Open* 2020; 10:e037547. doi: 10.1136/bmjopen-2020-037547

**HR**	Physical functioning	0.87	0.62	1.16	0.86	0.59	1.14	1.20	0.89	1.55						
	Psychological well-being	1.04	0.83	1.30	1.07	0.84	1.33	0.83	0.62	1.06						
	Social relations & part	0.92	0.70	1.17	1.00	0.77	1.26	0.91	0.67	1.17						
	Enjoyment of life	1.01	0.83	1.21	1.22	**1.02**	1.45	1.01	0.82	1.23						
	Resilience	0.81	0.63	1.01	0.81	0.63	1.01	1.03	0.81	1.29						
	Person-centeredness	1.14	0.91	1.39	1.08	0.86	1.33	0.95	0.73	1.19						
	Continuity of care	1.20	0.98	1.44	1.26	**1.03**	1.51	0.87	0.68	1.10						
	Total costs	1.00	0.55	1.59	0.70	0.36	1.18	1.20	0.67	1.91						
**HU**	Physical functioning	0.77	0.53	1.06	0.89	0.67	1.16	0.87	0.67	1.10						
	Psychological well-being	1.57	**1.14**	2.08	1.16	0.84	1.55	1.16	0.87	1.52						
	Social relations & part	1.01	0.70	1.35	0.93	0.69	1.22	1.05	0.82	1.32						
	Enjoyment of life	1.30	0.98	1.66	1.14	0.89	1.42	1.04	0.84	1.29						
	Resilience	0.80	0.57	1.08	0.77	0.58	1.01	0.65	0.50	**0.84**						
	Person-centeredness	0.90	0.64	1.19	1.32	**1.04**	1.66	1.01	0.79	1.25						
	Continuity of care	1.05	0.83	1.30	0.95	0.77	1.15	0.81	0.66	**0.97**						
	Total costs	0.60	0.29	1.07	0.83	0.44	1.32	1.42	0.91	2.09						
**NL**	Physical functioning	0.77	0.63	**0.92**	0.73	0.61	**0.87**				0.92	0.79	1.06	0.87	0.74	1.00
	Psychological well-being	0.91	0.74	1.12	1.10	0.93	1.32				1.12	0.94	1.34	0.91	0.77	1.09
	Social relations & part	0.97	0.76	1.22	1.15	0.96	1.38				1.09	0.90	1.32	1.09	0.91	1.30
	Enjoyment of life	1.11	0.96	1.27	0.99	0.87	1.13				1.05	0.92	1.20	0.96	0.85	1.09
	Resilience	0.95	0.81	1.11	0.83	0.71	**0.96**				0.77	0.65	**0.89**	0.93	0.81	1.06
	Person-centeredness	1.05	0.81	1.33	1.02	0.82	1.26				0.80	0.61	1.01	0.98	0.78	1.21
	Continuity of care	1.07	0.85	1.31	0.91	0.72	1.10				0.73	0.56	**0.91**	0.82	0.66	1.00
	Total costs	1.16	0.81	1.55	1.27	0.96	1.62				1.53	**1.19**	1.93	1.43	**1.12**	1.82
**NO**	Physical functioning	0.66	0.53	**0.80**	0.71	0.60	**0.83**				0.77	0.64	**0.90**	0.63	0.53	**0.75**
	Psychological well-being	1.08	0.90	1.27	0.98	0.84	1.14				0.87	0.72	1.03	0.86	0.73	1.01

	Social relations & part	1.32	**1.09**	1.56	1.19	**1.01**	1.40				0.97	0.79	1.17	1.18	0.98	1.39
	Enjoyment of life	1.12	0.94	1.32	1.11	0.94	1.29				1.04	0.87	1.23	0.99	0.84	1.15
	Resilience	0.83	0.64	1.06	1.00	0.84	1.19				1.09	0.90	1.32	1.07	0.88	1.29
	Person-centeredness	1.25	0.86	1.66	1.08	0.79	1.41				0.79	0.51	1.09	1.10	0.81	1.44
	Continuity of care	0.86	0.67	1.09	0.92	0.74	1.12				0.72	0.57	**0.92**	1.03	0.84	1.26
	Total costs	0.88	0.46	1.41	1.02	0.62	1.48				1.77	**1.25**	2.36	1.13	0.71	1.66
**UK**	Physical functioning	0.82	0.63	1.03	0.94	0.77	1.14	0.94	0.76	1.15						
	Psychological well-being	1.17	0.95	1.42	1.00	0.82	1.20	1.07	0.85	1.31						
	Social relations & part	1.06	0.84	1.29	1.03	0.83	1.25	1.01	0.79	1.25						
	Enjoyment of life	1.15	0.97	1.34	0.98	0.83	1.14	0.94	0.79	1.12						
	Resilience	1.01	0.79	1.26	0.97	0.78	1.17	1.04	0.83	1.29						
	Person-centeredness	1.06	0.79	1.34	1.11	0.87	1.39	1.18	0.90	1.50						
	Continuity of care	0.85	0.65	1.09	0.87	0.68	1.09	0.99	0.77	1.24						
	Total costs	0.89	0.57	1.24	1.11	0.81	1.48	0.82	0.52	1.17						
