# Supplementary material for: Comparing patients’ and other stakeholders’ preferences for outcomes of integrated care for multimorbidity: a discrete choice experiment in eight European countries
Source: BMJ Open. 2020 Oct 10;10(10):e037547. doi: 10.1136/bmjopen-2020-037547 (PMC7552858; doi:10.1136/bmjopen-2020-037547)
Supplement: Author's manuscript [file bmjopen-2020-037547.draft_revisions.pdf]

BMJ Open

BMJ Open is committed to open peer review. As part of this commitment we make the peer review history of every article we publish publicly available.

When an article is published we post the peer reviewers' comments and the authors' responses online. We also post the versions of the paper that were used during peer review. These are the versions that the peer review comments apply to.

The versions of the paper that follow are the versions that were submitted during the peer review process. They are not the versions of record or the final published versions. They should not be cited or distributed as the published version of this manuscript.

BMJ Open is an open access journal and the full, final, typeset and author-corrected version of record of the manuscript is available on our site with no access controls, subscription charges or pay-per-view fees (http://bmjopen.bmj.com).

If you have any questions on BMJ Open's open peer review process please email info.bmjopen@bmj.com

BMJ Open

Comparing patients' and other stakeholders' preferences for outcomes of integrated care for multimorbidity: a discrete choice experiment in eight European countries.

Journal:	*BMJ Open*
Manuscript ID	bmjopen-2020-037547
Article Type:	Original research
Date Submitted by the Author:	06-Feb-2020
Complete List of Authors:	Rutten-van Molken, Maureen; Erasmus Universiteit Rotterdam, Erasmus School of Health Policy & Management<br>Karimi, Milad; Erasmus Universiteit Rotterdam, Erasmus School of Health Policy & Management<br>Leijten, Fenna; Erasmus Universiteit Rotterdam, Erasmus School of Health Policy & Management ; Ministry of defence, Staff Defence Healthcare Organisation<br>Hoedemakers, Maaike; Erasmus Universiteit Rotterdam, Erasmus School of Health Policy & Management<br>Looman, Willemijn; Erasmus Universiteit Rotterdam, Erasmus School of Health Policy & Management<br>Islam, Kamrul; University of Bergen, Department of Economics<br>Askildsen, Jan; University of Bergen, Department of Economics<br>Kraus, Markus; Institut fur Hohere Studien,<br>Ercevic, Darija; Agency for Quality and Accreditation in Health Care and Social Welfare<br>Struckmann, Verena; Berlin University of Technology, Department of Health Care Management<br>Pitter, Janos; Syreon Research Institute<br>Cano, Isaac; Hospital Clinic de Barcelona, Institut d'Investigacions Biomèdiques August Pi i Sunyer (IDIBAPS)<br>Stokes, Jonathan; University of Manchester Institute of Population Health, Centre for Health Economics<br>Jonker, Marcel; Erasmus Universiteit Rotterdam, Health Policy & Management
Keywords:	PRIMARY CARE, HEALTH ECONOMICS, International health services < HEALTH SERVICES ADMINISTRATION & MANAGEMENT, Organisation of health services < HEALTH SERVICES ADMINISTRATION & MANAGEMENT, PUBLIC HEALTH

SCHOLARONE™
Manuscripts

I, the Submitting Author has the right to grant and does grant on behalf of all authors of the Work (as defined in the below author licence), an exclusive licence and/or a non-exclusive licence for contributions from authors who are: i) UK Crown employees; ii) where BMJ has agreed a CC-BY licence shall apply, and/or iii) in accordance with the terms applicable for US Federal Government officers or employees acting as part of their official duties; on a worldwide, perpetual, irrevocable, royalty-free basis to BMJ Publishing Group Ltd ("BMJ") its licensees and where the relevant Journal is co-owned by BMJ to the co-owners of the Journal, to publish the Work in this journal and any other BMJ products and to exploit all rights, as set out in our licence.

The Submitting Author accepts and understands that any supply made under these terms is made by BMJ to the Submitting Author unless you are acting as an employee on behalf of your employer or a postgraduate student of an affiliated institution which is paying any applicable article publishing charge ("APC") for Open Access articles. Where the Submitting Author wishes to make the Work available on an Open Access basis (and intends to pay the relevant APC), the terms of reuse of such Open Access shall be governed by a Creative Commons licence – details of these licences and which Creative Commons licence will apply to this Work are set out in our licence referred to above.

Other than as permitted in any relevant BMJ Author's Self Archiving Policies, I confirm this Work has not been accepted for publication elsewhere, is not being considered for publication elsewhere and does not duplicate material already published. I confirm all authors consent to publication of this Work and authorise the granting of this licence.

For peer review only

Comparing patients' and other stakeholders' preferences for outcomes of integrated care for multimorbidity: a discrete choice experiment in eight European countries.

Maureen Rutten-van Mölken[1,2], Milad Karimi[1], Fenna Leijten,[1,3] Maaike Hoedemakers[1], Willemijn Looman[1], Kamrul Islam[4], Jan Erik Askildsen[4], Markus Kraus[5], Darija Ercevic[6], Verena Struckmann[7], Janos Gyorgy Pitter[8], Isaac Cano[9], Jonathan Stokes[10], Marcel Jonker[1], on behalf of the SELFIE consortium

[1] School of Health Policy and Management, Erasmus University Rotterdam, Rotterdam, the Netherlands,

[2] Institute for Medical Technology Assessment, Erasmus University Rotterdam, Rotterdam, the Netherlands

[3] Staff Defence Healthcare Organisation, Dutch Ministry of Defence, the Netherlands

[4] Department of Economics, University of Bergen, Bergen, Norway

[5] Institute for Advanced Studies, Vienna, Austria

[6] Agency for Quality and Accreditation in Health Care and Social Welfare, Zagreb, Croatia

[7] Department of Health Care Management, Technische Universität Berlin, Berlin, Germany

[8] Syreon Research Institute, Budapest, Hungary

[9] Hospital Clinic de Barcelona, Institut d'Investigacions Biomèdiques August Pi i Sunyer (IDIBAPS), Universitat de Barcelona. Barcelona, Spain

[10] Centre for Primary Care and Health Services Research, University of Manchester, Manchester, United Kingdom

Email corresponding author Maureen Rutten-van Mölken: m.rutten@eshpm.eur.nl (MRvM)

ORCID-ID: 0000-0001-8706-3159

Keywords: multimorbidity, preferences, discrete choice experiment, integrated care, Triple Aim, informal care, policy making, SELFIE, Multi-Criteria Decision Analysis, Europe

Target journal: BMJ; Word count 4628

For peer review only - http://bmjopen.bmj.com/site/about/guidelines.xhtml

Abstract (word count 300)

Objectives: To measure relative preferences for outcomes of integrated care of patients with multimorbidity from eight European countries and compare them to the preferences of other stakeholders within these countries.

Design: A discrete choice experiment (DCE) was conducted in each country, asking respondents to choose between two integrated care programmes for persons with multimorbidity.

Setting: Preference data collected in Austria(AT), Croatia(HR), Germany(DE), Hungary(HU), Netherlands(NL), Norway(NO), United Kingdom(UK).

Participants: Patients with multimorbidity, partners & other informal caregivers, professionals, payers, policy makers.

Main outcome measures: Each integrated care programme was described in terms of health/wellbeing, experience with care, and cost outcomes, i.e., physical functioning, psychological wellbeing, social relationships & participation, enjoyment of life, resilience, person-centeredness, continuity of care, and total costs. Each outcome had three levels of performance.

Results: 5122 respondents completed the DCE. In all countries, patients with multimorbidity, as well as most other stakeholder groups, assigned the (second) highest preference to enjoyment of life. The patients top-three most frequently included physical functioning, psychological wellbeing, and continuity of care. Continuity of care also entered the top-three of professionals, payers and policy makers in four countries (AT, DE, HR, HU). Of the five stakeholder groups, preferences of professionals differed most often from preferences of patients. Professionals assigned lower weights to physical functioning in AT, DE, ES, NL, NO and higher weights to person-centeredness in AT, DE, ES, HU. Payers and policy makers assigned higher weights than patients to costs, but these weights were relatively low.

Conclusion: The wellbeing outcome enjoyment of life is the most important outcome of integrated care in multimorbidity. This calls for a greater involvement of social and mental care providers. The difference in opinion between patients and professionals calls for shared decision making, whereby efforts to improve wellbeing and person-centeredness should not divert attention from improving physical functioning.

For peer review only

For peer review only - http://bmjopen.bmj.com/site/about/guidelines.xhtml

Strengths and limitations

We designed an online DCE to measure the relative importance of health, well-being, experience, and cost outcomes of integrated care to patients with multimorbidity from eight European countries.

The choice of outcomes was largely driven by focus groups involving patients with multimorbidity.

We compared patients' preferences with the preferences of other stakeholders including informal caregivers, professionals, payers, and policy makers in a large sample of 5122 respondents.

Online discrete choice experiments are cognitively challenging and respondents may not represent all patients with multimorbidity.

When completing the DCE, it might be challenging for the other stakeholders to separate their professional role from the other roles they might have.

For peer review only

For peer review only - http://bmjopen.bmj.com/site/about/guidelines.xhtml

Introduction

Persons with multimorbidity may have complex care needs requiring the involvement of different types of professionals from different sectors of the healthcare, social care and/or community care systems. If the services provided by different professionals are not well aligned, they may experience conflicting treatment goals, harmful effects of interacting treatments, overly-demanding appeals on self-management capabilities, and inefficiencies like over- and under-treatment.

It is hypothesized, that persons with multimorbidity would benefit from person-centered integrated care programmes.[1-3] Integrated care programmes commonly adopt a holistic approach towards people with multimorbidity, which means that, instead of the disease, the person with his or her needs, capabilities, preferences, resources, and (social) environment is put in the centre.[4] In the EU-funded Horizon2020 project SELFIE (Sustainable intEgrated chronic care modeLs for multimorbidity: delivery, FInancing, and performancE), we study 17 promising integrated care programmes from eight European countries, i.e., Austria (AT), Croatia (HR), Germany (DE), Hungary (HU), the Netherlands (NL), Norway (NO), Spain (ES) and the United Kingdom (UK). In many of these programmes an interdisciplinary individual care plan, with well-aligned health and social care services, is designed to specifically target the goals and priorities jointly set by the person with multimorbidity and his or her formal and informal care givers. Many programmes also offer tailored self-management support and assign a case manager to the most complex patients, to ensure that they have a single contact point that helps them navigate through the system.

Integrated care programmes for persons with multimorbidity often aim to improve outcomes that go beyond health and include their broader sense of wellbeing, experience with care, and costs. This is often referred to as the Triple Aim of improving population health/wellbeing, experience and costs.[5] Therefore, we decided to measure a broad set of outcome measures in the evaluation studies of the 17 programmes in the SELFIE project. However, the importance of these outcomes is almost never measured. In the SELFIE project we were specifically interested in the importance of these outcomes to persons with multimorbidity. Understanding their preferences is vital for the optimal design of person-centered integrated care services and inform decision making on implementation and upscaling of these services. In the SELFIE project, we aim to use the preferences as weights in Multi-Criteria Decision Analyses (MCDA) of the 17 programmes.[6] In an MCDA, a wide range of different outcomes is aggregated into a single, overall value score. This overall value score combines the performance of the integrated care programmes on different outcomes, each weighted according to its respective preference weight.[7,8] Besides the persons with multi-morbidity, there are many other stakeholders involved in decision making about integrated care and their preferences may differ from patients' preferences. Therefore, we aim to repeat the MCDA's in the SELFIE project, using the preference weights from different groups of stakeholders.

The aim of the current paper is twofold. Firstly, to present the relative magnitude of the preferences for the Triple Aim outcomes in persons with multimorbidity from eight European countries. Secondly, the aim is to compare the Patients' preferences to the preferences of Partners & other informal caregivers, Professionals, Payers and Policy makers, together referred to as the 5 P's. This will be a within-country comparison because the decision context of the future MCDA's relates to local or regional level decisions about the adaptation, reimbursement, continuation, and scaling-up of the 17 existing integrated care programmes.

For peer review only - http://bmjopen.bmj.com/site/about/guidelines.xhtml

Methods

Discrete choice experiment (DCE)

The preferences were obtained using discrete choice experiments (DCEs). A DCE is a theoretically well founded method to determine how much respondents are willing to trade-off some outcomes against others.[9-11] In our DCE, respondents from the 5P's were given questions that asked them to choose between two integrated care programmes for persons with multimorbidity, labelled programme A and programme B. There was no opt-out. Each programme was described in terms of outcomes with systematically varying levels that describe the performance of the programme on the outcomes. The outcomes included measures of health and wellbeing: 1) physical functioning, 2) psychological wellbeing, 3) social relationships & participation, 4) enjoyment of life, 5) resilience; measures of experience: 6) person-centeredness, 7) continuity of care; and 8) total health- and social care costs. The definitions are given in Table 1. An example of a DCE question is given in Figure 1.

(Table 1. Definitions of the outcomes and levels)

(Figure 1. Example of a DCE question)

Each outcome (attributes in DCE terminology) had three levels indicating a poor, average and good performance of the programme on that outcome. The definitions of the outcomes and levels were identical across the eight countries, except for costs. The three levels of costs were based on country-specific estimates of the mean total health and social care costs for people with multimorbidity in 2017 (middle level) and increased and decreased by 20% to obtain the poor and good performance level. This percentage was chosen to ensure enough variation in levels while at the same time remaining within realistic boundaries. The costs were expressed in the national currency.

DCE design

A Bayesian efficient heterogeneous design algorithm was used to optimise the DCE designs based on the D-efficiency criterion.[12] Ten different sub-designs with 18 choice tasks per sub-design were used. In each sub-design either four or five attribute levels were overlapped between programme A and B, to reduce the overall complexity of the choice tasks and improve response efficiency.[13] One restriction was built into the design, namely, the best level of the psychological wellbeing attribute and the worst level of enjoyment of life, and vice versa, were not presented simultaneously for the same programme.

The initial DCE designs were optimized using priors that were based on the available literature (See online supplementary file Box S1). These DCE designs were subsequently updated, based upon updated priors that reflected the information obtained from approximately the first 50 respondents in each stakeholder group. The design updates were performed separately for each country/stakeholder group and the updated priors were the average of the literature-based priors and the conditional logit estimates of the first 50 respondents.

For peer review only - http://bmjopen.bmj.com/site/about/guidelines.xhtml

Questionnaire design

The online DCE survey started with an explanation of the SELFIE project, the outcomes, type of questions, and the perspective from which the questions should be answered (i.e. one of the five P's). Each respondent completed a randomly chosen sub-design with 18 choice tasks, preceded by two practice-questions. The order of appearance of the choice tasks within each sub-design was randomised. The 18 choice tasks were presented in three sections of six choice tasks each, with several background questions in between. The background questions related to sociodemographic characteristics and the respondents' health conditions. Colour coding was used for the different levels of the outcomes as shown in Figure 1, and outcomes that had the same level in the two integrated care programmes were presented in grey.[13] When respondents hovered the mouse over an outcome, the definition of that outcome became visible. At the end of the survey, respondents received a multiple-choice question on the level of difficulty of the questionnaire.

The questionnaire was designed in English and translated into each country's respective language, using the same translation protocol, which included forward and backward translations by native-language speakers with an excellent level of English.

Respondents

Respondents from the stakeholder groups were recruited in the eight countries participating in the SELFIE project during 2017-2018. Sample size calculations[14] led to a target number of 150 respondents per country per stakeholder group. Each country recruited respondents from three to five different groups of stakeholders. All countries recruited respondents from the first three P's (i.e., patients with multimorbidity, partners & other informal caregivers, professionals). The Netherlands and Norway recruited respondents from all five P's. The other countries combined the respondents from the payers and policy makers into one group, either because they have a Beveridge-like healthcare model in which there is a national/regional health service with a single payer (HR, HU, UK, ES) or because they were in the midst of a comprehensive health insurance reform (AT) or an election (DE), making it difficult to recruit a sufficient number of respondents from the two groups separately.

Patients with multimorbidity and *partners & other informal caregivers* were recruited from internet panels managed by market research organisations, except in Spain where they were personally invited to participate by the members of the Spanish team of the SELFIE project who were physicians and nurses. In Spain, patients were recruited in the waiting rooms of a hospital, geriatric residences, and support groups for families with Alzheimer. All patients had two or more chronic conditions. Partners & other informal caregivers had to provide voluntary care or support to a family member, friend, or other acquaintance who needed help due to physical, mental or ageing-related health problems, for at least two weeks.

Professionals were also recruited through an internet panel organisation in the UK and the Netherlands. In the other countries, they were recruited through the networks of the authors and a snowball sampling method, in which we asked respondents if they could invite other responders within their organisations. Examples of organisations approached include care provider organisations or professional associations like nursing associations.

For peer review only - http://bmjopen.bmj.com/site/about/guidelines.xhtml

All *payers* and *policy makers* in the UK and about one third of the payers and one sixth of the policy makers in the Netherlands were recruited via an internet panel. Payers had to have paid employment in an occupation in which they were (in)directly involved in the financing or payment of integrated care and/or care for persons with multimorbidity. Policy makers had to be (in)directly involved in policy- or decision making about health care, social care or welfare. In the other countries, payers and policy makers were recruited through our own networks and snowball sampling. Examples of payer organisations that were approached include health care insurance companies and departments of municipalities responsible for paying social care. Examples of policy makers include politicians, public servants from the ministries of health, ministries of social care, provincial or local governments, and official governmental advisory bodies.

Patient and public involvement

The selection of the outcomes in the DCE was driven by the results of eight focus groups with in total 58 persons that had multiple chronic conditions. These focus groups were organised in 2016 in the countries participating in the SELFIE project.[15] As the participants of these focus groups frequently mentioned the importance of outcomes that went beyond the traditional health outcomes such as physical functioning, we included enjoyment of life, social relationships & participation, and resilience to represent aspects of wellbeing and 'positive health'.[16] They also frequently mentioned negative experiences related to the single-disease focus of each provider, misaligned treatment goals and advices, and fragmentation or duplication of care. That is the reason why 'person-centeredness' and 'continuity of care' were identified as the two key elements of experience of care.

The selection of outcomes was further informed by national workshops with stakeholder representatives in these countries, an international advisory board meeting within the SELFIE project (see acknowledgement), a review of outcomes measured in the 17 integrated care programmes, and a literature review on outcome measures of integrated care.[17] In each country, two to four persons with multimorbidity and two to four informal caregivers were present at the national workshops. One patient representative and one representative from an informal caregiver organisation were present at the international advisory board meeting.

The online DCE survey was pilot tested in six elderly with multimorbidity from the Netherlands, including two think-aloud completions of the online questionnaire. Their feedback was used to improve the visual design and instructions of the questionnaire.

The results of the weight elicitation study were discussed in national stakeholder workshops and the international advisory board meetings of the SELFIE project, where both patients and informal caregivers were present. They were reimbursed for their travel and accommodation costs and did not receive other remuneration.

Ethics approval

We adhered to the national regulations regarding medical ethics approvals and waivers. Letters of Medical Ethics Approval of the MCDA's and waivers were send to and approved by the European

For peer review only - http://bmjopen.bmj.com/site/about/guidelines.xhtml

Commission as a Deliverable of the SELFIE project. All respondents read the project information and provided online consent to take part before starting the online DCE survey.

Statistical analysis

A scale heterogeneity multinomial logit (S-MNL) model was used to analyse the responses by stakeholder group.[18,19] This model contains respondent-specific scale parameters that account for differential choice variability caused by the fact that in some respondents the error-terms are more important relative to the observed attribute-coefficients than in others. This is often interpreted as a difference in choice consistency.[20] The model also included a dummy variable to test whether respondents were more likely to choose the integrated care programme that was presented left or right. As this was not the case, this dummy was not included in the final model.

The relative importance weight of each of the included attributes was calculated as the contribution of the attribute's best level coefficient (level 3) to the sum of all attributes' best level coefficients. This was done because the weights, that will later be used in the MCDA's of the integrated care programmes,[6] need to represent the preference for the full swing from the worst to the best level of an attribute.

Differences in the mean preferences of stakeholder groups were evaluated using S-MNL models that analyzed all stakeholder groups within a country simultaneously. Multiplicative interaction terms were used to assess the difference in preferences between the first stakeholder group (i.e. patients with multimorbidity) and each of the other stakeholder groups. There is an indication of a difference when the 95% credible interval (CI) of the interaction term of a particular stakeholder group for a particular attribute does not contain 1.

All models were programmed in the BUGS language and estimated using OpenBUGS using Bayesian Markov Chain Monte Carlo (MCMC) methods (Syntax provided in online supplementary file Box S2).

Results

Respondents

Altogether, 5122 respondents completed the study, among which 1314 patients with multimorbidity. Of all patients who consented to complete the questionnaire, the proportion that completed all DCE questions was above 75%, except in Austria (67%). The mean time to complete the questionnaire was around 25 minutes (See online supplementary file Box S3).

Table 2 presents the characteristics of the respondents. Patients were older, less educated, in poorer health, and less often had a paid job than the other stakeholders. The health of the partners & informal caregivers was less good than the health of the professionals, payers and policy makers. There was a relatively high proportion of females among the professionals. The top five of most frequently reported diseases (not risk factors) among patients across all countries included 1) depression, anxiety or emotional difficulties, 2) chronic back pain or sciatica, 3) diabetes, 4) stomach problem, ulcer, gastritis or reflux, and 5) cardiovascular diseases (See online supplementary file Box S4).

For peer review only - http://bmjopen.bmj.com/site/about/guidelines.xhtml

(Table 2. Characteristics of the respondents included in the analysis)

Preferences of patients with multimorbidity

Table 3 shows the results of the regression analyses for the patients with multimorbidity. A positive regression coefficient means that respondents had a higher preference for (i.e., placed a higher weight on) that level of the outcome than level one. The larger the coefficient the stronger the preference. All outcomes contributed significantly to patients' choices, in the expected direction. The DCE-coefficients of outcome-levels two and three were almost all significantly different from level one. The coefficients of level three were always higher than of level two, except for non-significant disordered levels of social relationships & participation and person-centeredness in Germany, continuity of care in Norway and Spain, and costs in Spain.

The magnitude of the preference for the best level (level 3) was used as an indicator of the importance of the outcome measures. In all countries, except Hungary and Spain, enjoyment of life was valued most by patients, followed by either continuity of care (HR, DE), psychological wellbeing (NL, UK), physical functioning (NO), or resilience (AT). In Hungary, patients assigned the highest value to continuity of care, whereas in Spain, patients assigned the highest value to psychological wellbeing, both followed by enjoyment of life. In six of the eight countries (not HR and HU), physical functioning was placed in the top three. Social relationships & participation, person-centeredness and costs were generally valued lower by the patients than other outcomes. The scale parameter was highest in Austria, indicating that the difference in choice consistency was greatest among the Austrian patients.

(Table 3. Preferences for outcomes of integrated care of patients with multimorbidity)

Within-country comparison of preferences of patients and other stakeholders

Figure 2 presents the relative preference weights, sorted by importance among patients with multimorbidity. Within countries, there was considerable agreement between patients and the other stakeholders, with enjoyment of life mostly at the top and costs at the bottom. Nevertheless, there were differences.

As an indication of these differences, we counted the number of times that the 95% credibility intervals of the multiplicative interaction terms for stakeholder group and attribute excluded one (See online supplementary file Box S5). Of all stakeholder groups, the preferences of professionals differed most often from the preferences of patients. These differences were most frequently related to physical functioning and person-centeredness. Physical functioning was valued lower by professionals than patients in Austria, Germany, Netherlands, Norway and Spain. Person-centeredness was valued higher by professionals than patients in Austria, Germany, Hungary, and Spain.

Differences in relative weights between patients and payers/policy makers within a country were most frequently related to costs, which was more important to the latter in Austria, Germany, the Netherlands, and Norway. However, the relative weight that they assigned to costs remained low.

For peer review only - http://bmjopen.bmj.com/site/about/guidelines.xhtml

Payers and/or policy makers assigned lower values to continuity of care (HU, NL, NO), and resilience (DE, ES, HU, NL) than patients.

When the partners & other informal caregivers' weights differed from the patients, that mostly related to physical functioning, which was valued lower than patients in Austria, Germany, Netherlands, and Norway and higher than patients in Spain.

(Figure 2. Relative weight of the outcomes by stakeholder and country.)

Discussion

Main findings

This is the first international preference study among such a large number of persons with multimorbidity and other stakeholders involved in decision making about person-centered integrated care. We found that there was considerable agreement between patients, partners & other informal caregivers, professionals, payers and policy makers. In all countries, they assigned the highest or second highest value to enjoyment of life and the lowest or second lowest value to costs. Of the health and wellbeing outcomes, psychological wellbeing, physical functioning and resilience were generally valued higher than social relationships & participation. Of the two experience outcomes, continuity of care was generally valued higher than person-centeredness. Patients as well as the other stakeholders in the two Eastern European countries, Hungary and Croatia, assigned a relatively high value to continuity of care. Continuity of care also entered the top-three of professionals, payers and policy makers in Germany and Austria. Representatives from Eastern European countries suggested that the high preference assigned to continuity of care might illustrate problems with access to care and long waiting lists in various parts of their healthcare systems. German and Austrian stakeholders also pointed to a relation with capacity issues in especially the elderly care in their countries.

We also found indications of within-country differences in preferences between patients and other stakeholders. Of all stakeholder groups, professionals' preferences differed most frequently from patients' preferences and these differences most often pertained to physical functioning and person-centeredness. Professionals assigned lower values to physical functioning than patients in five countries, and higher values to person-centeredness in four countries. Preference heterogeneity was also found for costs, which received higher values from payers and policy makers than from patients in four countries. However, the relative value of costs compared to the other outcomes remained low for these stakeholders as well. When discussing why costs commonly ranked last, even among payers and policy makers, stakeholder representatives generally felt that this was in not in line with real world decision making. The low value that patients assigned to costs might be related to the fact that they were informed that these costs did not have to be paid out-of-pocket. The low value assigned by payers and policy makers might be a reflection of the normative viewpoint that costs should not play an important role when it comes to health. It might also be related to the challenging task of separating their professional role from the other roles they might have. Payers and policy makers can easily relate to, or might be, patients and informal caregivers themselves.

Comparison with previous research

For peer review only - http://bmjopen.bmj.com/site/about/guidelines.xhtml

In contrast to most previous DCE studies of similar interventions, we defined the DCE attributes in terms of Triple Aim outcomes and not in terms of characteristics of the services provided. The choices made by the respondents indicated that they did make trade-offs between health, wellbeing and experience outcomes. That finding is in line with a recent study that also measured preferences for both health and wellbeing outcomes in the same valuation procedure. They did so in response to the recognition that the Quality Adjusted Life Year (QALY) does not capture the full value of interventions that involve social care.[21] Mulhern et al. included the health domains of the EQ-5D (EuroQol five-dimension questionnaire) and the wellbeing domains of the ASCOT (Adult Social Care Outcomes Toolkit) in the same DCE and found that the top-4 of most valued outcomes by the Australian general public included one ASCOT domain, namely 'control over ones daily life', which ranked fourth after the EQ-5D domains pain and discomfort, mobility and anxiety/depression.[21] They further found that social participation got the lowest value. This is in line with our study, where this outcome ended-up in the bottom-three in five countries (AT, DE, HR, HU, NL), based on patients' values.

Mühlbacher and Bethge conducted a series of online DCE's among US patients and German insurees (not only patients) that focused on different features and building blocks of integrated service delivery.[22] In contrast to our study, costs were an important attribute in both countries. It ranked highest in Germany and in the US only 'shared decision making' was slightly more important. The difference is most likely explained by the fact that costs were defined as out-of-pocket costs.

Burton et al, conducted a DCE that specifically aimed to value different aspects of person-centeredness in UK patients with either chronic pain or chronic respiratory disease. They found that 'taking account of the current living situation', and 'working with the patients on what they want to get from life' were the most important aspects.[23] Although our definition of person-centeredness as 'care that matches an individual's needs, capabilities, and preferences and where decisions are made jointly based on good information' more or less incorporated these two aspects, that did not prevent this outcome to be ranked relatively low when it was compared to the other outcomes by the patients in Western European countries.

Limitations of the study

The first limitation is the cognitive challenge of completing a DCE. Respondents are expected to understand and interpret the definitions of the outcomes as provided. For that reason, we did not only explain the outcomes before the start of the DCE task, but also ensured that the definitions of the outcomes became visible in every choice task when respondents hovered the mouse over an outcome-heading. Respondents are further expected to consider all outcomes and make choices based on trade-offs between all outcomes. It has been reported in the literature that, although the majority of respondents does this, lower educated and less health literate respondents more often use simplified heuristics such as basing their choices on one or two high priority attributes.[24] To reduce the complexity of the choices, we have used attribute-level overlap and colour coding. Nevertheless, the proportion of patients that found the questionnaire (very) difficult varied from 8.4% in Hungary to 35.3% in Norway. However, the observation that only a small proportion of patients in all countries completed the questionnaire in less than five minutes may illustrate their engagement in the DCE task. Excluding these patients from the analyses did not affect the results (available upon request).

For peer review only - http://bmjopen.bmj.com/site/about/guidelines.xhtml

The second limitation is related to the assumption of preference independence underlying an MCDA,[8] which means that the preference for a particular outcome is not influenced by the level of performance on the other outcomes. This was an important criterion when selecting the outcomes for the DCE. It was also the reason why enjoyment of life was framed positively (having pleasure and happiness in life) and psychological wellbeing was framed negatively (the absence of stress, worrying, listlessness, anxiety, and feeling down). However, that does not avoid conceptual overlap entirely. We acknowledge that enjoyment of life is a broad outcome measure that might be associated with some of the other outomes measures.

A last limitation concerns the generalizability of the results, especially of the persons with multimorbidity and the informal caregivers which were recruited through an existing internet panel. Because of that, respondents with lower levels of education, health literacy and health numeracy are likely to be underrepresented in our study. This does not apply to Spain, where patients were recruited by professionals, which might have led to an over-representation of patients with high healthcare utilization and informal caregivers with a higher burden of informal care.

Implications

First, the high preference for enjoyment of life, which was defined as having pleasure and happiness in life, underlines the importance of measuring outcomes beyond health to assess the wider value of integrated care programmes for people with multimorbidity. This is not often done, and certainly not routinely. Second, this high preference for enjoyment of life calls for more attention for social and mental support, something which is not exclusively provided by the healthcare sector. Patients care more about the big picture, i.e., the pleasure and happiness in life, rather than the process of care, unless there is something clearly lacking in this process, such as continuity of care. Third, although it is good that many of the current integrated care programmes already pay a lot of attention to wellbeing and person-centeredness, these efforts should not divert attention from improving physical functioning where possible. Fourth, the differences in relative preferences for physical functioning and person-centeredness between patients and professionals in several countries stress the need for shared decision making to prioritise services that improve outcomes most important to patients. Fifth, the existence of preference heterogeneity with respect to outcomes of integrated care among stakeholders stresses the importance of informing decision makers from multiple perspectives. The MCDA's of the 17 integrated care programmes that we are conducting are particularly suited for that.[7] These MCDA's will be used to inform pragmatic, local and regional level decisions on different options to implement, adapt, or scale-up existing integrated care programmes. In this context, the downside of an MCDA of using an outcome that does not allow a comparison between integrated care and other interventions, like the QALY, is acceptable. When such a comparison is required for system-level reimbursement decisions, it can be informative to compare the results of the MCDA to the results of a cost per QALY analysis.

Conclusion

Integrated care programmes aim to improve multiple outcomes. The most important outcome to patients with multimorbidity was the wellbeing outcome enjoyment of life, which calls for a greater involvement of the social and mental care sector. This also stresses the importance of including

For peer review only - http://bmjopen.bmj.com/site/about/guidelines.xhtml

outcomes beyond health into the evaluation of these programmes. Process-related outcomes were less important to patients unless there were issues with the process, like the lack of continuity of care. The other stakeholders largely agreed with the preferences of the patients, although the preference heterogeneity with respect to physical functioning and person-centeredness underlines the importance of shared decision making between patients with multimorbidity and professionals.

For peer review only

For peer review only - http://bmjopen.bmj.com/site/about/guidelines.xhtml

Acknowledgements

We would like to thank all the anonymous respondents who devoted time to the completion of the weight-elicitation study. We are grateful for the contributions of the members of the international advisory board of the SELFIE project and the participants of the national workshops. The advisory board consisted of Rudi Westendorp (Center for Healthy Ageing), Ronald van Breugel (health insurer VGZ), Jan de Maeseneer (Emeritus professor of Family Medicine), Mieke Rijken (NIVEL), Viktoria Stein (International Foundation of Integrated Care), Luke Slawomirski (OECD), Ian Forde (formerly OECD), Rick Greene (International Alliance of Carer Organizations), Nadine Henningsen (Canadian Homecare Organization), Dominik Tomek, (European Patients' Forum), Derek Steward (NHS NIHR, Patient and Public Health Involvement), Antonia Croy (patient advocate), and Ikka Kunnamo (Editor in Chief Evidence-Based Medicine Guidelines, Duodecim Medical Publications). We are also grateful for the input that we have received from the participants of the national workshops in the eight participating countries.

For peer review only

For peer review only - http://bmjopen.bmj.com/site/about/guidelines.xhtml

Funding statement

The SELFIE project has received funding from the European Union's Horizon 2020 research and innovation program under grant agreement No 634288.

Disclaimer

The content of this paper reflects only the SELFIE group's views and the European Commission is not liable for any use that may be made of the information contained herein.

For peer review only

Conflict of interest statement

All authors have completed the ICMJE uniform disclosure form at www.icmje.org/coi_disclosure.pdf and declare: all authors had financial support from the European Union's Horizon 2020 research and innovation program under grant agreement No 634288 for the submitted work; no financial relationships with any organisations that might have an interest in the submitted work; no other relationships or activities that could appear to have influenced the submitted work.

Data sharing statement

The individual-level DCE data will not be made publicly available. Upon request to the corresponding author, we may consider in collaboration with the respective SELFIE-partner, whether country-specific data can be shared. Other findings from the overarching SELFIE study can be found on the SELFIE website https://www.selfie2020.eu/.

Contributorship statements

MRvM was the coordinator of SELFIE, designed the study, had access to the data from all countries, supervised the analyses, controlled the decision to publish, and wrote the paper. She accepts full responsibility for the conduct of the study.

MK was part of the coordinating team of SELFIE, contributed to data analysis, interpretation of data for all countries, and writing the paper.

FL was part of the coordinating team of SELFIE, co-designed the study, contributed to data collection, data analyses, interpretation of data for all countries, and reviewed the paper.

MH was part of the coordinating team of SELFIE, co-designed the study, contributed to data collection, data analyses, interpretation of data for all countries, and reviewed the paper.

WL was part of the coordinating team of SELFIE, co-designed the study, contributed to data collection, data analyses, interpretation of data for all countries, and reviewed the paper

KI contributed to the translation of the DCE into Norwegian, data collection and interpretation in Norway, and reviewed the paper.

JEA contributed to the translation of the DCE for Norway, data collection and interpretation in Norway, and reviewed the paper.

MKr contributed to the translation of the DCE for Austria, data collection and interpretation in Austria, and reviewed the paper.

VS contributed to the translation of the DCE for Germany, data collection and interpretation in Germany, and reviewed the paper.

DR contributed to the translation of the DCE for Croatia, data collection and interpretation in Croatia, and reviewed the paper.

JGP contributed to the translation of the DCE for Hungary, data collection and interpretation in Hungary, and reviewed the paper.

IC contributed to the translation of the DCE for Spain, data collection and interpretation in Spain, and reviewed the paper.

JS contributed to the translation of the DCE for the UK, data collection and interpretation in the UK, and reviewed the paper.

MJ optimised the DCE designs, conducted the Bayesian statistical analyses, and reviewed the paper.

For peer review only

For peer review only - http://bmjopen.bmj.com/site/about/guidelines.xhtml

Table 1. Definitions of the outcomes and levels.

Outcome (attribute)	Definition	Levels
Physical functioning	acceptable physical functioning and being able to do daily activities without needing assistance (e.g., getting dressed, sitting down and getting up from a chair, taking your medications)	1. Severely limited in physical functioning and activities of daily living<br>2. Moderately limited in physical functioning and activities of daily living<br>3. Hardly or not at all limited in physical functioning and activities of daily living
Psychological wellbeing	the absence of stress, worrying, listlessness, anxiety, and feeling down	1. Always or mostly stressed, worried, listless, anxious, and down<br>2. Regularly stressed, worried, listless, anxious, and down<br>3. Seldom or never stressed, worried, listless, anxious, and down
Social relationships & participation	having meaningful connections with others as desired	1. No or barely any meaningful connections with others<br>2. Some meaningful connections with others<br>3. A lot of meaningful connections with others
Enjoyment of life	having pleasure and happiness in life	1. No or barely any pleasure and happiness in life<br>2. Some pleasure and happiness in life<br>3. A lot of pleasure and happiness in life
Resilience	the ability to recover from or adjust to difficulties and to restore one's balance	1. Poor ability to recover, adjust, and restore balance<br>2. Fair ability to recover, adjust, and restore balance<br>3. Good ability to recover, adjust, and restore balance
Person-centeredness	care that matches an individual's needs, capabilities, and preferences and where decisions are made jointly based on good information	1. Not or barely person-centered<br>2. Somewhat person-centered<br>3. Highly person-centered
Continuity of care	good collaboration, smooth transitions between caregivers, and no waste of time	1. Poor collaboration, transitions, and timeliness<br>2. Fair collaboration, transitions, and timeliness<br>3. Good collaboration, transitions, and timeliness
Total health- and social care costs	total health- and social care costs per participant in the programme, per year. Note:	1. 8500 euro per participant per year *<br>2. 7000 euro per participant per year<br>3. 5500 euro per participant per year

For peer review only - http://bmjopen.bmj.com/site/about/guidelines.xhtml

	These are costs paid for by the health insurer / government.

* These are the Dutch values. The value for the other countries are: Austria: 8,000, 6,600, 5,200 euro; Croatia: 7,200, 6,000, 4,800 kuna (973, 810, 648 euro); Hungary: 600,000, 500,000, 400,000 forint (1,951, 1,626, 1,300 euro); Germany: 4,800, 4,000, 3,200 euro; Norway: 115,000, 95,000, 75,000 krone (12,330, 10,185, 8,041 euro); Spain: 5,400, 4,500, 3,600 euro; United Kingdom: 3,600, 3,000, 2,400 pounds (4,130, 3,441, 2,753 euro)

For peer review only

For peer review only - http://bmjopen.bmj.com/site/about/guidelines.xhtml

Table 2. Characteristics of the respondents included in the analysis.

	Patients	Partners	Professionals	Payers	Policy makers
**Austria**	N=169	N=184	N=144	N=99 (combined)	
Age mean (SD)	51.0 (15.0)	42.9 (14.7)	44.7 (8.8)	45.8 (10.7)	
Gender (% female)	78 (46.2)	103 (56.0)	110 (77.5)	53 (53.5)	
Educational level (%)^					
Low	15 (8.9)	10 (5.4)	0 (0)	0 (0)	
Medium	126 (74.5)	125 (67.9)	40 (28.1)	15 (15.2)	
High	28 (16.6)	49 (26.6)	102 (71.8)	81 (84.9)	
General health (%)					
Poor or Fair	87 (51.5)	30 (16.3)	4 (2.8)	2 (2)	
Good	67 (39.6)	70 (38.0)	33 (23.2)	23 (23.2)	
Very good or excellent	15 (8.9)	70 (45.7)	102 (71.8)	24 (74.7)	
Number of health problems (mean, SD)	3.4 (2.2)	2.0 (2.0)	0.7 (1.1)	0.6 (1.0)	
**Croatia**	N=168	N=169	N=103	N=73 (combined)	
Age	43.1 (13.7)	42.6 (10.8)	44.4 (11.5)	45.3 (10.3)	
Gender (% female)	104 (61.9)	96 (56.8)	79 (76.7)	62 (84.9)	
Educational level (%)^					
Low	87 (51.8)	91 (53.9)	7 (6.8)	5 (6.8)	
Medium	39 (23.2)	33 (19.5)	38 (36.9)	26 (35.6)	
High	42 (25.0)	45 (26.7)	58 (56.4)	42 (57.5)	
General health (%)					
Poor or Fair	74 (44.1)	25 (14.8)	9 (8.8)	8 (10.9)	
Good	44 (26.2)	27 (16.0)	26 (25.2)	21 (28.8)	
Very good or excellent	50 (29.8)	117 (69.3)	68 (66.0)	44 (60.2)	
Number of health problems (mean, SD)	3.2 (2.3)	1.6 (1.6)	1.2 (1,3)	1.25 (1.6)	
**Germany**	N=160	N=208	N=170	N=110 (combined)	
Age	52.7 (14.6)	46.8 (14.5)	42.4 (10.7)	45.6 (11.0)	
Gender (% female)	77 (48.1)	102 (49.0)	123 (73.7)	65 (59.1)	
Educational level (%)^					
Low	53 (33.2)	76 (36.5)	31 (18.6)	2 (1.8)	
Medium	85 (53.2)	90 (43.2)	68 (40.7)	36 (32.7)	
High	22 (13.9)	42 (20.2)	68 (40.7)	72 (65.4)	
General health (%)					
Poor or Fair	106 (66.3)	49 (23.5)	18 (10.8)	8 (7.3)	
Good	48 (30.0)	98)47.1)	66 (39.5)	36 (32.7)	
Very good or excellent	6 (3.8)	61 (29.4)	83 (49.7)	66 (60.0)	
Number of health problems (mean, SD)	4.7 (2.5)	2.6 (2.7)	1.4 (1.7)	0.87 (1.2)	
**Hungary**	N=192	N=166	N=163	N=153 (combined)	
Age	51.1 (14.1)	43.9 (14.3)	45.4 (10.9)	46.7 (10.8)	
Gender (% female)	102 (53.1)	80 (48.2)	84 (51.5)	101 (66.0)	
Educational level (%)^					

For peer review only - http://bmjopen.bmj.com/site/about/guidelines.xhtml

	Patients	Partners	Professionals	Payers	Policy makers
Low	65 (33.9)	51 (30.7)	1 (0.6)	3 (2.0)	
Medium	70 (36.5)	80 (48.2)	13 (8.0)	21 (13.7)	
High	57 (29.7)	35 (21.1)	149 (90.4)	129 (84.3)	
General health (%)					
Poor or Fair	120 (62.5)	44 (26.5)	23 (14.1)	18 (11.8)	
Good	61 (31.8)	71 (42.8)	46 (28.2)	50 (32.7)	
Very good or excellent	11 (5.7)	51 (30.7)	94 (57.7)	85 (55.6)	
Number of health problems (mean, SD)	4.0 (2.4)	2.1 (2.1)	1.1 (1.5)	1.1 (1.4)	
**Netherlands**	N=156	N=158	N=155	N=104	N=151
Age	60.4 (11.8)	52.2 (13.0)	42.0 (12.5)	42.8 (11.7)	46.3 (12.1)
Gender (% female)	77 (49.0)	81 (51.3)	86 (55.5)	48 (46.2)	95 (62.9)
Educational level (%)^					
Low	14 (9.0)	8 (5.1)	1 (0.6)	0	2 (1.3)
Medium	70 (45.0)	76 (48.1)	48 (31.0)	1 (1.0)	0
High	72 (46.2)	74 (46.8)	106 (68.3)	103 (99.1)	149 (89.7)
General health (%)					
Poor or Fair	86 (55.2)	40 (25.3)	11 (7.1)	7 (6.7)	14 (9.2)
Good	63 (40.4)	77 (48.7)	59 (38.1)	38 (36.5)	51 (33.8)
Very good or excellent	7 (4.5)	42 (26.0)	85 (54.9)	59 (56.8)	86 (57.0)
Number of health problems (mean, SD)	4.0 (1.9)	2.2 (2.5)	1.1 (1.5)	0.7 (1.0)	0.8 (1.2)
**Norway**	N=156	N=156	N=162	N=122	N=180
Age	56.5 (14.7)	53.7 (12.9)	45.7 (10.7)	51.3 (10.3)	54.7 (13.2)
Gender (% female)	70 (44.9)	78 (50.00)	124 (76.5)	68 (55.7)	97 (53.9)
Educational level (%)^					
Low	11 (7.1)	1 (0.6)	2 (1.2)	1 (0.8)	0
Medium	52 (33.3)	39 (25.0)	4 (2.5)	1 (0.8)	35 (19.4)
High	93 (59.6)	116 (74.4)	156 (96.3)	120 (98.4)	145 (80.6)
Poor or Fair	78 (50.0)	38 (24.4)	8 (4.9)	5 (4.1)	17 (9.4)
Good	47 (30.1)	64 (41.0)	33 (20.4)	17 (13.9)	50 (27.8)
Very good or excellent	31 (19.9)	54 (34.6)	121 (74.7)	100 (82)	113 (62.8)
Number of health problems (mean, SD)	3.6 (1.7)	2.2 (1.7)	1.1 (1.5)	0.79 (1.1)	1.5 (1.6)
**Spain**	N=150	N=151	N=152		
Age	62.8 (9.8)	55.3 (11.7)	40.8 (10.7)		
Gender (% female)	65 (43.3)	103 (68.2)	40.76 (10.65)		
Educational level (%)^					
Low	68 (45.4)	57 (37.8)	0		
Medium	39 (26)	54 (35.7)	30 (19.7)		
High	43 (28.7)	40 (26.4)	122 (80.3)		
General health (%)					
Poor or Fair	69 (46.0)	45 (29.8)	2 (1.3)		
Good	63 (42.0)	70 (46.4)	30 (19.7)		

For peer review only - http://bmjopen.bmj.com/site/about/guidelines.xhtml

	Patients	Partners	Professionals	Payers	Policy makers
Very good or excellent	18 (12.0)	36 (23.8)	120 (78.9)		
Number of health problems (mean, SD)	3.9 (2.5)	2.08 (1.7)	0.9 (1.0)		
**United Kingdom**	N=163	N=233	N=161	N=181 (combined)	
Age	56.4 (14.4)	45 (13.8)	47.7 (10.8)	45.5 (11.0)	
Gender (% female)	94 (57.7)	126 (54.1)	51 (31.7)	74 (40.9)	
Educational level (%)^					
Low	60 (36.8)	60 (25.8)	0	3 (1.7)	
Medium	45 (27.6)	57 (24.5)	2 (1.2)	13 (7.2)	
High	58 (35.6)	116 (49.7)	159 (98.8)	165 (91.1)	
General health (%)					
Poor or Fair	129 (79.2)	61 (26.2)	5 (3.1)	18 (10)	
Good	28 (17.2)	75 (32.2)	15 (9.3)	33 (18.2)	
Very good or excellent	6 (3.7)	97 (41.7)	141 (87.6)	130 (71.8)	
Number of health problems (mean, SD)	4.6 (2.4)	1.9 (2.1)	0.7 (1.0)	1.1 (1.6)	

^low: no post-secondary education; high: Bachelor's degree or higher

For peer review only

For peer review only - http://bmjopen.bmj.com/site/about/guidelines.xhtml

Table 3. Preferences for outcomes of integrated care of patients with multimorbidity.

		Coefficients Bayesian S-MNL model: mean and 95% credibility interval															
Outcome	Level	AT		HR		HU		DE		NL		NO		ES		UK	
Physical functioning	2	1.11	0.73-1.78	0.59	0.40-0.77	0.47	0.30-0.63	0.81	0.61-1.02	1.25	1.00-1.50	1.74	1.46-2.02	1.41	1.14-1.71	0.92	0.72-1.13
	3	1.19	0.79-1.87	0.71	0.51-0.91	0.69	0.50-0.87	1.00	0.77-1.24	1.95	1.65-2.28	1.93	1.61-2.26	1.44	1.14-1.78	1.22	0.99-1.46
Psycholo-gical wellbeing	2	0.43	0.22-0.71	0.65	0.47-0.82	0.005	-0.14-0.16	0.25	0.07-0.43	0.94	0.73-1.17	0.94	0.72-1.16	1.26	0.97-1.59	0.55	0.37-0.73
	3	0.98	0.63-1.57	1.05	0.83-1.28	0.33	0.16-0.50	0.64	0.41-0.88	2.03	1.72-2.38	1.89	1.60-2.19	1.69	1.37-2.03	1.30	1.08-1.54
Social relations & participation	2	0.53	0.34-0.78	0.67	0.50-0.84	0.37	0.22-0.52	0.52	0.34-0.70	0.85	0.66-1.06	1.08	0.85-1.30	1.17	0.90-1.45	0.78	0.60-0.97
	3	0.62	0.39-0.97	0.81	0.61-1.01	0.58	0.41-0.75	0.48	0.30-0.68	1.03	0.81-1.27	1.20	0.97-1.44	1.24	0.98-1.54	1.02	0.83-1.22
Enjoyment of life	2	1.14	0.81-1.71	0.95	0.77-1.14	0.59	0.42-0.75	0.98	0.79-1.19	1.80	1.54-2.08	1.94	1.66-2.24	1.63	1.31-1.98	1.50	1.28-1.73
	3	1.56	1.15-2.28	1.41	1.18-1.65	0.72	0.54-0.91	1.36	1.12-1.62	2.76	2.45-3.14	2.68	2.31-3.08	1.65	1.30-2.05	2.16	1.88-2.47
Resilience	2	0.89	0.58-1.44	0.72	0.55-0.91	0.60	0.44-0.76	0.72	0.54-0.92	1.37	1.15-1.59	0.85	0.65-1.04	1.06	0.82-1.31	0.79	0.60-0.98
	3	1.19	0.85-1.80	1.13	0.93-1.34	0.71	0.54-0.89	1.04	0.83-1.27	1.81	1.56-2.08	1.19	0.97-1.42	1.18	0.92-1.47	1.09	0.88-1.30
Person-centered-ness	2	0.05	-0.17-0.25	0.68	0.51-0.86	0.46	0.31-0.62	0.31	0.14-0.48	0.52	0.32-0.71	0.31	0.12-0.50	0.63	0.43-0.84	0.47	0.31-0.64
	3	0.31	0.11-0.53	0.96	0.78-1.15	0.71	0.53-0.90	0.26	0.08-0.44	0.95	0.74-1.16	0.50	0.31-0.70	0.72	0.50-0.95	0.72	0.53-0.90
Continuity of care	2	0.66	0.43-1.00	0.91	0.73-1.10	0.86	0.70-1.03	0.66	0.48-0.84	1.08	0.85-1.31	1.30	1.07-1.54	0.70	0.49-0.93	0.72	0.53-0.91
	3	0.97	0.70-1.38	1.19	0.99-1.40	1.00	0.82-1.18	1.08	0.85-1.32	1.28	1.05-1.53	1.20	0.97-1.44	0.63	0.42-0.85	0.94	0.74-1.15
Total costs	2	0.38	0.15-0.69	0.21	0.06-0.37	0.15	-0.002-0.30	0.18	0.02-0.34	0.17	-0.01-0.34	0.07	-0.12-0.25	0.35	0.15-0.56	0.36	0.18-0.54

For peer review only

For peer review only - http://bmjopen.bmj.com/site/about/guidelines.xhtml

3	0.44	0.21-0.76	0.24	0.07-0.42	0.19	0.03-0.36	0.27	0.10-0.44	0.43	0.24-0.62	0.23	0.05-0.42	0.15	-0.06-0.38	0.58	0.40-0.77
Scale parameter tau	1.39	0.21-1.12	0.57	0.38-0.77	0.72	0.47-1.02	1.07	0.75-1.60	0.65	0.48-0.84	0.58	0.41-0.78	1.23	0.83-2.05	0.56	0.38-0.74
No. of observations	6,120		6,228		6,984		5,940		5,760		5,688		5,400		5,940	
No. of respondents	170		173		194		166		160		158		150		165	

For peer review only

	Care programme A	Care programme B
ⓘ **Physical functioning**	**Severely** limited in physical functioning and activities of daily living	**Moderately** limited in physical functioning and activities of daily living
ⓘ **Psychological well-being**	**Always or mostly** stressed, worried, listless, anxious, and down	**Always or mostly** stressed, worried, listless, anxious, and down
ⓘ **Social relationships and participation**	**No or barely any** meaningful connections with others	**No or barely any** meaningful connections with others
ⓘ **Enjoyment of life**	**Some** pleasure and happiness in life	**No or barely any** pleasure and happiness in life
ⓘ **Resilience**	**Good** ability to recover, adjust, and restore balance	**Good** ability to recover, adjust, and restore balance
ⓘ **Person-centeredness**	**Highly** person-centred	**Highly** person-centred
ⓘ **Continuity of care**	**Poor** collaboration, transitions, and timeliness	**Fair** collaboration, transitions, and timeliness
ⓘ **Total health- and social care costs**	**5600 pounds** per participant per year	**7000 pounds** per participant per year
**Which care programme do you prefer, A or B?**	**A** ○	**B** ○

Figure 1. Example of a DCE question.

only

For peer review only - http://bmjopen.bmj.com/site/about/guidelines.xhtml

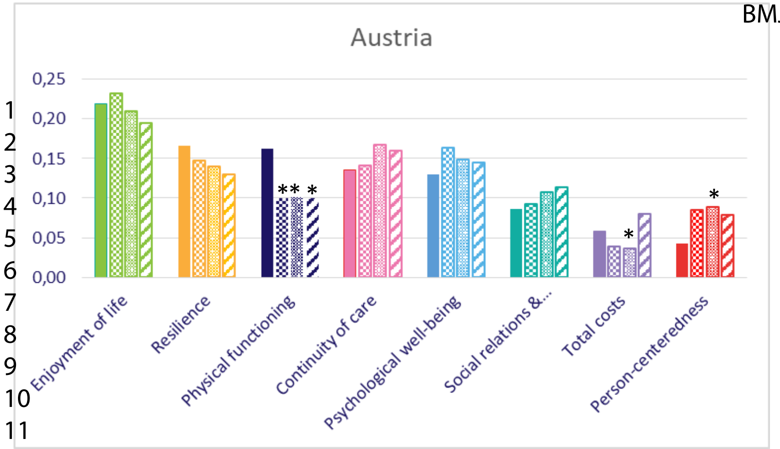
Austria
0,25
0,20
0,15
0,10
0,05
0,00
Enjoyment of life
Resilience
Physical functioning
Continuity of care
Psychological well-being
Social relations &...
Total costs
Person-centeredness

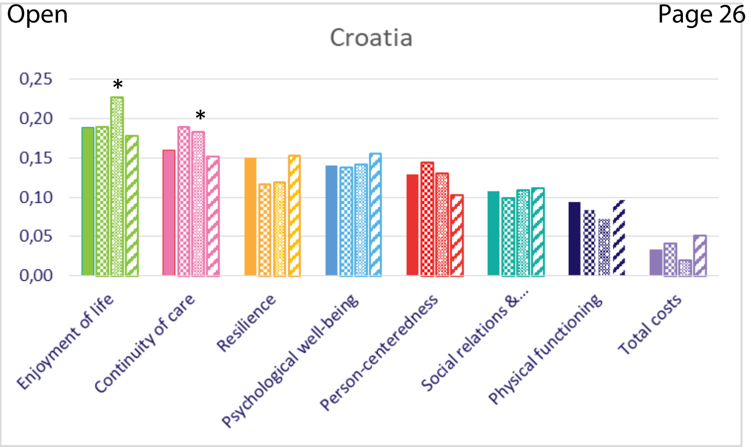
Croatia
0,25
0,20
0,15
0,10
0,05
0,00
Enjoyment of life
Continuity of care
Resilience
Psychological well-being
Person-centeredness
Social relations &...
Physical functioning
Total costs

[redacted]

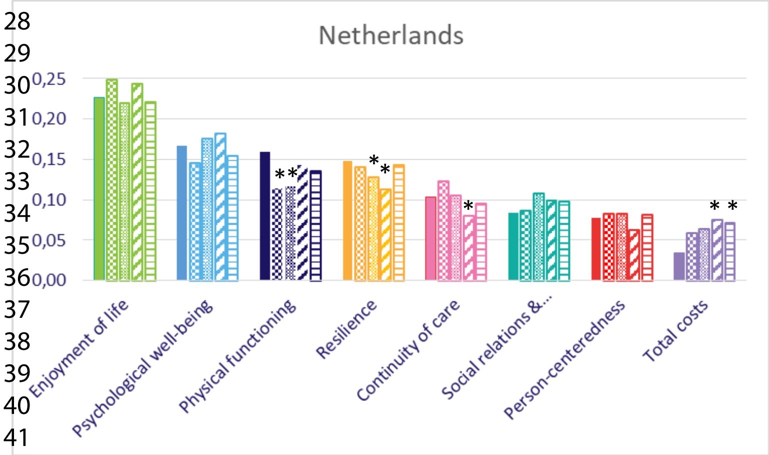
Netherlands
0,25
0,20
0,15
0,10
0,05
0,00
Enjoyment of life
Psychological well-being
Physical functioning
Resilience
Continuity of care
Social relations &...
Person-centeredness
Total costs

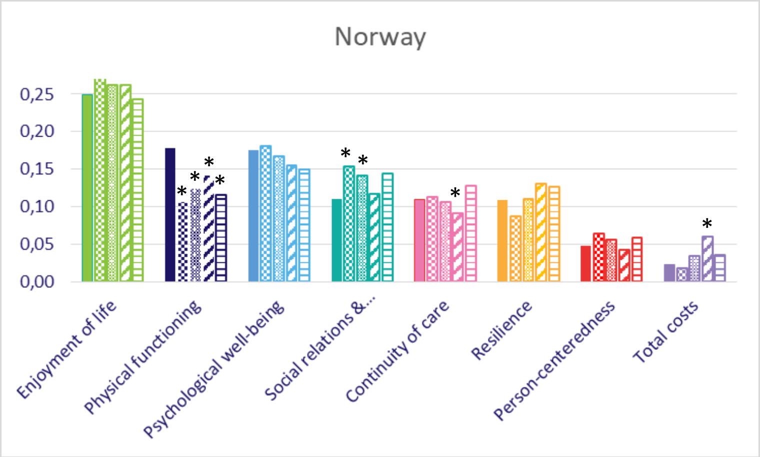
Norway
0,25
0,20
0,15
0,10
0,05
0,00
Enjoyment of life
Physical functioning
Psychological well-being
Social relations &...
Continuity of care
Resilience
Person-centeredness
Total costs

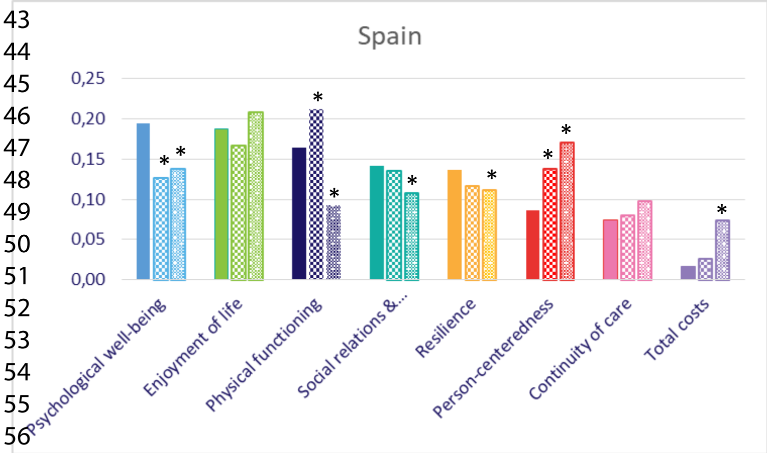
Spain
0,25
0,20
0,15
0,10
0,05
0,00
Psychological well-being
Enjoyment of life
Physical functioning
Social relations &...
Resilience
Person-centeredness
Continuity of care
Total costs

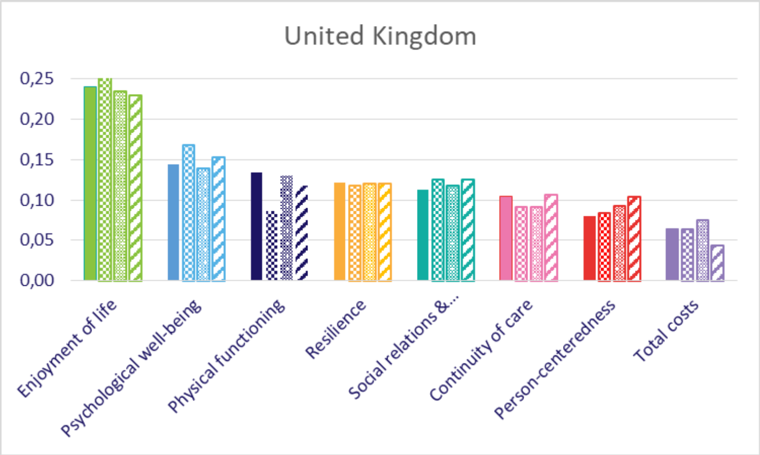
United Kingdom
0,25
0,20
0,15
0,10
0,05
0,00
Enjoyment of life
Psychological well-being
Physical functioning
Resilience
Social relations &...
Continuity of care
Person-centeredness
Total costs

Figure 2. Relative preferences of all stakeholders sorted by the preferences of patients with multi-morbidity (order of bars at each outcome: patients, partners and other informal caregivers, professionals, payers and policy makers); * 95% credibility interval excludes 1, indicating that the stakeholder group differed from the patients with multi-morbidity (See online supplementary file box S5). The relative weight of the partners and other informal caregivers is 0 in Hungary.

For peer review only

Data used in Figure 2. Relative preferences

Austria	patients	partners	professionals	payers/policy makers
Enjoyment of life	0.22	0.23	0.21	0.19
Resilience	0.17	0.15	0.14	0.13
Physical functioning	0.16	0.10	0.10	0.10
Continuity of care	0.14	0.14	0.17	0.16
Psychological wellbeing	0.13	0.16	0.15	0.15
Social relations & participation	0.09	0.09	0.11	0.11
Total costs	0.06	0.04	0.04	0.08
Person-centeredness	0.04	0.08	0.09	0.08

Croatia	patients	partners	professionals	payers/policy makers
Enjoyment of life	0.19	0.19	0.23	0.18
Continuity of care	0.16	0.19	0.18	0.15
Resilience	0.15	0.12	0.12	0.15
Psychological wellbeing	0.14	0.14	0.14	0.16
Person-centeredness	0.13	0.14	0.13	0.10
Social relations & participation	0.11	0.10	0.11	0.11
Physical functioning	0.09	0.08	0.07	0.10
Total costs	0.03	0.04	0.02	0.05

Germany	patients	partners	professionals	payers/policy makers
Enjoyment of life	0.22	0.22	0.18	0.18
Continuity of care	0.17	0.24	0.22	0.16
Physical functioning	0.17	0.06	0.08	0.12
Resilience	0.17	0.16	0.13	0.12
Psychological wellbeing	0.11	0.07	0.11	0.13
Social relations & participation	0.08	0.08	0.09	0.08
Total costs	0.04	0.08	0.06	0.11
Person-centeredness	0.04	0.09	0.12	0.09

For peer review only

Hungary	patients	partners	professionals	payers/policy makers
Continuity of care	0.20	0.21	0.19	0.17
Enjoyment of life	0.15	0.20	0.17	0.16
Person-centeredness	0.15	0.12	0.19	0.16
Resilience	0.14	0.10	0.10	0.10
Physical functioning	0.14	0.10	0.11	0.12
Social relations & participation	0.12	0.11	0.10	0.12
Psychological wellbeing	0.07	0.15	0.10	0.11
Total costs	0.04	0.00	0.03	0.06

Netherlands	patients	partners	professionals	payers	policy makers
Enjoyment of life	0.23	0.25	0.22	0.24	0.22
Psychological wellbeing	0.17	0.15	0.18	0.18	0.15
Physical functioning	0.16	0.11	0.12	0.14	0.14
Resilience	0.15	0.14	0.13	0.11	0.14
Continuity of care	0.10	0.12	0.11	0.08	0.10
Social relations & participation	0.08	0.09	0.11	0.10	0.10
Person-centeredness	0.08	0.08	0.08	0.06	0.08
Total costs	0.03	0.06	0.06	0.07	0.07

Norway	patients	partners	professionals	payers	policy makers
Enjoyment of life	0.25	0.27	0.26	0.26	0.24
Physical functioning	0.18	0.11	0.12	0.14	0.12
Psychological wellbeing	0.17	0.18	0.17	0.16	0.15
Social relations & participation	0.11	0.15	0.14	0.12	0.14
Continuity of care	0.11	0.11	0.11	0.09	0.13
Resilience	0.11	0.09	0.11	0.13	0.13
Person-centeredness	0.05	0.06	0.06	0.04	0.06
Total costs	0.02	0.02	0.03	0.06	0.04

Spain	patients	partners	professionals
Psychological wellbeing	0.19	0.13	0.14
Enjoyment of life	0.19	0.17	0.21
Physical functioning	0.16	0.21	0.09
Social relations & participation	0.14	0.14	0.11
Resilience	0.14	0.12	0.11
Person-centeredness	0.09	0.14	0.17
Continuity of care	0.07	0.08	0.10
Total costs	0.02	0.03	0.07

For peer review only - http://bmjopen.bmj.com/site/about/guidelines.xhtml

UK	patients	partners	professionals	payers/policy makers
Enjoyment of life	0.24	0.26	0.23	0.23
Psychological wellbeing	0.14	0.17	0.14	0.15
Physical functioning	0.13	0.09	0.13	0.12
Resilience	0.12	0.12	0.12	0.12
Social relations & participation	0.11	0.12	0.12	0.13
Continuity of care	0.10	0.09	0.09	0.11
Person-centeredness	0.08	0.08	0.09	0.10
Total costs	0.06	0.06	0.07	0.04

For peer review only

For peer review only - http://bmjopen.bmj.com/site/about/guidelines.xhtml

References

1. Banerjee S. Multimorbidity--older adults need health care that can count past one. *Lancet* 2015;385(9968):587-89.
2. Rijken M, Hujala A, van Ginneken E, et al. Managing multimorbidity: Profiles of integrated care approaches targeting people with multiple chronic conditions in Europe. *Health policy* 2018;122(1):44-52.
3. Struckmann V, Leijten FRM, van Ginneken E, et al. Results of a scoping review on integrated care for persons with multi-morbidity. *Health policy* 2018;122(1):23-35.
4. Leijten FRM, Struckmann V, van Ginneken E, et al. The SELFIE framework for integrated care for multi-morbidity: Development and description. *Health policy* 2018;122(1):12-22.
5. Berwick DM, Nolan TW, Whittington J. The triple aim: care, health, and cost. *Health affairs* 2008;27(3):759-69.
6. Rutten-van Mölken MPMH, Leijten F, Hoedemakers M, et al. Strengthening the evidence-base of integrated care for people with multi-morbidity in Europe using Multi-Criteria Decision Analysis (MCDA). *BMC health services research* 2018;18:576.
7. Thokala P, Duenas A. Multiple criteria decision analysis for health technology assessment. *Value in health : the journal of the International Society for Pharmacoeconomics and Outcomes Research* 2012;15(8):1172-81.
8. Marsh K, M IJ, Thokala P, et al. Multiple Criteria Decision Analysis for Health Care Decision Making--Emerging Good Practices: Report 2 of the ISPOR MCDA Emerging Good Practices Task Force. *Value in health : the journal of the International Society for Pharmacoeconomics and Outcomes Research* 2016;19(2):125-37.
9. Hensher D, Rose J, Greene W. Applied Choice Analysis: A Primer.: Cambridge: Cambridge University Press 2005.
10. Louviere J, Hensher D, J S. Stated Choice Methods: Analysis and Application.: Cambridge: Cambridge University Press 2000.
11. Ryan M, Farrar S. Using conjoint analysis to elicit preferences for health care. *Britisch Medical Journal* 2000;320(7248):1530-3.
12. Sandor Z, Wedel M. Heterogeneous Conjoint Choice Designs. *J Marketing Research* 2005;42(2):210-18.
13. Jonker MF, Donkers B, de Bekker-Grob E, et al. Attribute level overlap (and color coding) can reduce task complexity, improve choice consistency, and decrease the dropout rate in discrete choice experiments. *Health economics* 2019;28(3):350-63.
14. de Bekker-Grob EW, Donkers B, Jonker MF, et al. Sample Size Requirements for Discrete-Choice Experiments in Healthcare: a Practical Guide. *The patient* 2015;8(5):373-84.
15. Leijten FRM, Hoedemakers M, Struckmann V, et al. Defining good health and care from the perspective of persons with multimorbidity: results from a qualitative study of focus groups in eight European countries. *BMJ open* 2018;8(8):e021072.
16. Huber M, Knottnerus JA, Green L, et al. How should we define health? *Bmj* 2011;343:d4163.
17. Boland M, Leijten F, Hoedemakers M, et al. Outcomes and indicators in integrated care for persons with multi-morbidity. 2017. https://www.selfie2020.eu/wp-content/uploads/2018/05/SELFIE-WP4-Deliverable-Report-1.pdf.
18. Fiebig DG, Keane MP, Louviere J, et al. The generalized multinomial logit model: accounting for scale and coefficient heterogeneity. *Marketing Science* 2010;29(3):393-421.

For peer review only - http://bmjopen.bmj.com/site/about/guidelines.xhtml

19. Greene WH, Hensher DA. Does scale heterogeneity across individuals matter? An empirical assessment of alternative logit models. *Transportation* 2010;37(3):413-28.
20. Lancsar E, Fiebig DG, Hole AR. Discrete Choice Experiments: A Guide to Model Specification, Estimation and Software. *PharmacoEconomics* 2017;35(7):697-716.
21. Mulhern B, Norman R, De Abreu Lourenco R, et al. Investigating the relative value of health and social care related quality of life using a discrete choice experiment. *Social science & medicine* 2019;233:28-37.
22. Muhlbacher A, Bethge S. Patients Preferences. In: Ameling V SU, Goodwin N, Balicer R, Nolte E, Suter E,, ed. Handbook Integrated Care. 1st ed. Cham Switzerland: Springer 2017.
23. Burton CD, Entwistle VA, Elliott AM, et al. The value of different aspects of person-centred care: a series of discrete choice experiments in people with long-term conditions. *BMJ open* 2017;7(4):e015689.
24. Veldwijk J, Determann D, Lambooij MS, et al. Exploring how individuals complete the choice tasks in a discrete choice experiment: an interview study. *BMC medical research methodology* 2016;16:45.

For peer review only

For peer review only - http://bmjopen.bmj.com/site/about/guidelines.xhtml

	Care programme A	Care programme B
ⓘ **Physical functioning**	**Severely** limited in physical functioning and activities of daily living	**Moderately** limited in physical functioning and activities of daily living
ⓘ **Psychological well-being**	**Always or mostly** stressed, worried, listless, anxious, and down	**Always or mostly** stressed, worried, listless, anxious, and down
ⓘ **Social relationships and participation**	**No or barely any** meaningful connections with others	**No or barely any** meaningful connections with others
ⓘ **Enjoyment of life**	**Some** pleasure and happiness in life	**No or barely any** pleasure and happiness in life
ⓘ **Resilience**	**Good** ability to recover, adjust, and restore balance	**Good** ability to recover, adjust, and restore balance
ⓘ **Person-centeredness**	**Highly** person-centred	**Highly** person-centred
ⓘ **Continuity of care**	**Poor** collaboration, transitions, and timeliness	**Fair** collaboration, transitions, and timeliness
ⓘ **Total health- and social care costs**	**5600 pounds** per participant per year	**7000 pounds** per participant per year
**Which care programme do you prefer, A or B?**	**A** ◯	**B** ◯

only

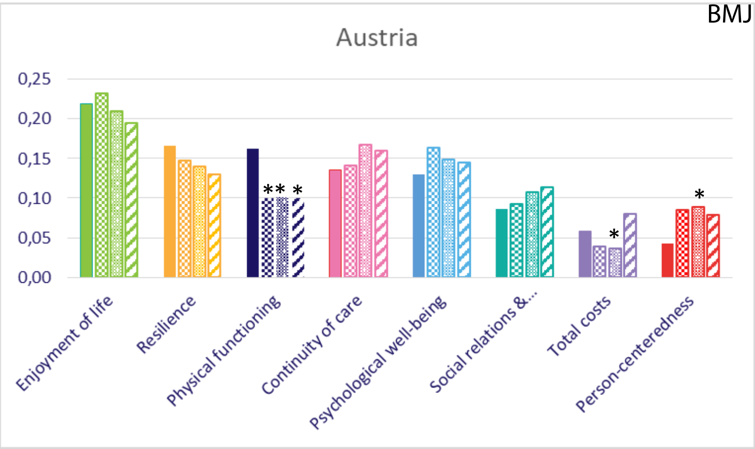
Austria
0,25
0,20
0,15
0,10
0,05
0,00
Enjoyment of life
Resilience
Physical functioning
Continuity of care
Psychological well-being
Social relations &...
Total costs
Person-centeredness

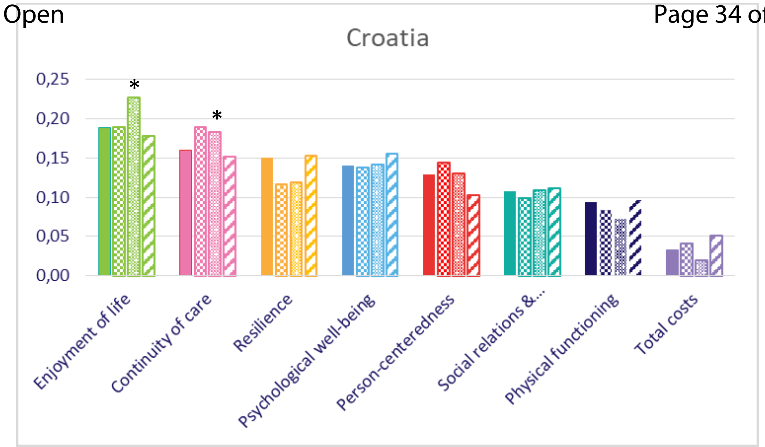
Croatia
0,25
0,20
0,15
0,10
0,05
0,00
Enjoyment of life
Continuity of care
Resilience
Psychological well-being
Person-centeredness
Social relations &...
Physical functioning
Total costs

[redacted]

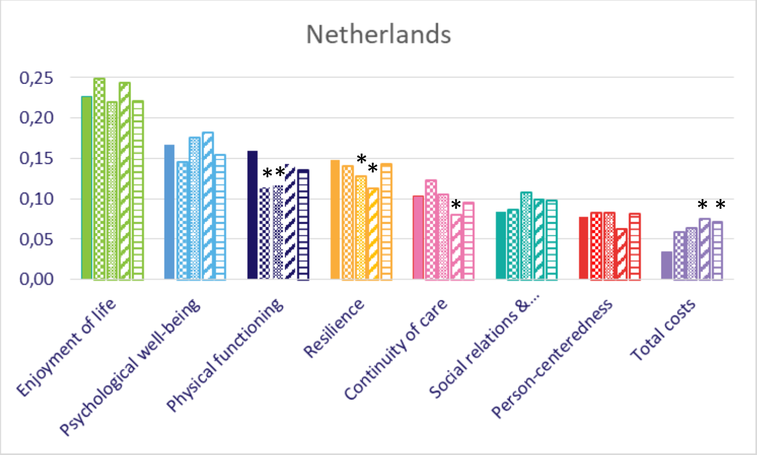
Netherlands
0,25
0,20
0,15
0,10
0,05
0,00
Enjoyment of life
Psychological well-being
Physical functioning
Resilience
Continuity of care
Social relations &...
Person-centeredness
Total costs

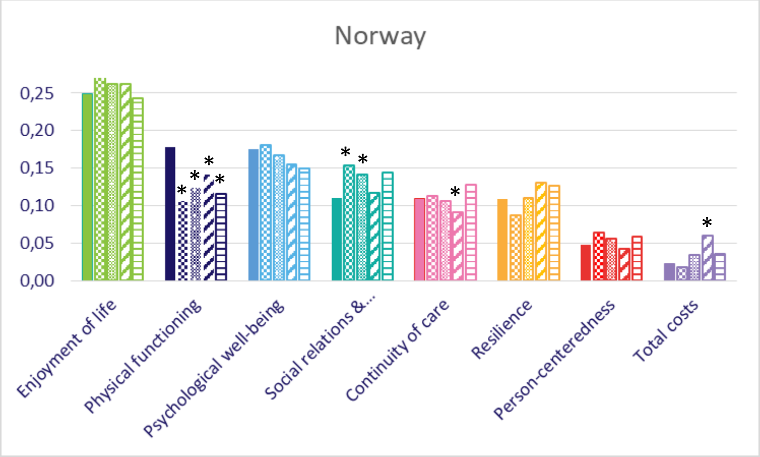
Norway
0,25
0,20
0,15
0,10
0,05
0,00
Enjoyment of life
Physical functioning
Psychological well-being
Social relations &...
Continuity of care
Resilience
Person-centeredness
Total costs

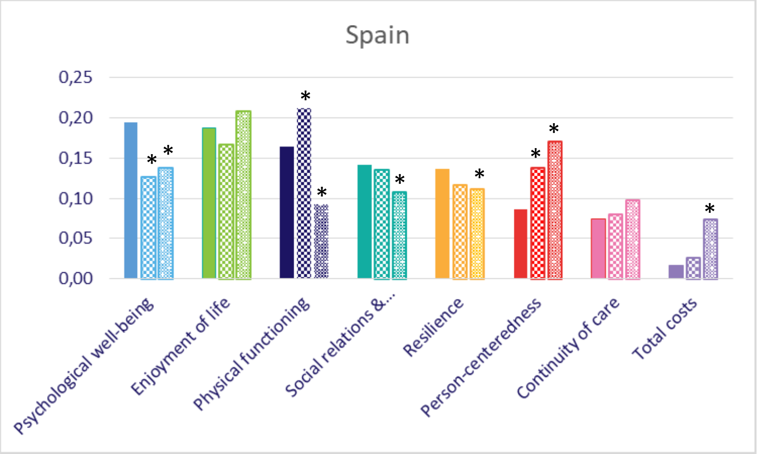
Spain
0,25
0,20
0,15
0,10
0,05
0,00
Psychological well-being
Enjoyment of life
Physical functioning
Social relations &...
Resilience
Person-centeredness
Continuity of care
Total costs

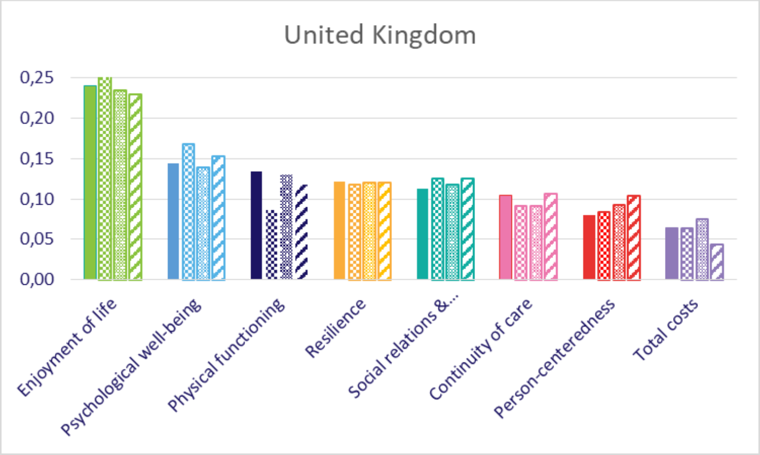
United Kingdom
0,25
0,20
0,15
0,10
0,05
0,00
Enjoyment of life
Psychological well-being
Physical functioning
Resilience
Social relations &...
Continuity of care
Person-centeredness
Total costs

Online supplementary file

Box S1. Original, literature-based, priors.

	DCE-Level		
**DCE-Attribute**	**Worst (1)**	**Average (2) Mean (SD)**	**Best (3) Mean (SD)**
Physical functioning	Reference	0.30 (0.12)	0.60 (0.23)
Psychological wellbeing	Reference	0.20 (0.08)	0.40 (0.16)
Social relations & participation	Reference	0.25 (0.10)	0.45 (0.18)
Enjoyment of life	Reference	0.20 (0.08)	0.40 (0.16)
Resilience	Reference	0.20 (0.08)	0.40 (0.16)
Person-centeredness	Reference	0.10 (0.04)	0.25 (0.10)
Continuity of care	Reference	0.15 (0.06)	0.30 (0.12)
Total costs	Reference	0.15 (0.06)	0.35 (0.14)

For peer review only

Box S2. Example of OpenBUGS model code for the S-MNL models.

All models were programmed in the BUGS language and estimated using OpenBUGS using Bayesian Markov Chain Monte Carlo (MCMC) methods. This entails selecting prior distributions for the unknown model parameters and updating these via the likelihood of the observed choice data. The specification of the priors and the model codes are presented below. All estimations used 10,000 MCMC iterations to let three chains converge from divergent starting points and another 20,000 MCMC iterations to reliably approximate the posterior distributions of the parameters. Convergence was evaluated based on a visual inspection of the MCMC chains and the diagnostics as included in the OpenBUGS software. Custom MCMC slice samplers were used for the GMNL-scale and the rho parameters; these are available from the authors upon request.

Model #1. Separate S-MNL models for each stakeholder group

```
model {

 # N  = number of respondents
 # N1 = number of respondents in P1 group
 # N2 = number of respondents in P2 group
 # N3 = number of respondents in P3 group
 # N4 = number of respondents in P4 group
 # T  = number of choice tasks
 # V  = number of parameters

 # likelihood of pairwise choice tasks
 for (n in 1:N){
   for(t in 1:T){
     Y[n,t] <- 0
     Y[n,t] ~ dcat2(phi[n,t])
 }}

 # utility function P1
 for (n in 1:N1){
   for (t in 1:T){
     phi[n,t]  <- (attr1[n,t] + attr2[n,t] + attr3[n,t] + attr4[n,t] + attr5[n,t] +
                  attr6[n,t] + attr7[n,t] + attr8[n,t]) * gmnl_scale[n]
     attr1[n,t] <- (beta[1,1]  * X[n,t,1]  + beta[1,2]  * X[n,t,2] )
     attr2[n,t] <- (beta[1,3]  * X[n,t,3]  + beta[1,4]  * X[n,t,4] )
     attr3[n,t] <- (beta[1,5]  * X[n,t,5]  + beta[1,6]  * X[n,t,6] )
     attr4[n,t] <- (beta[1,7]  * X[n,t,7]  + beta[1,8]  * X[n,t,8] )
     attr5[n,t] <- (beta[1,9]  * X[n,t,9]  + beta[1,10] * X[n,t,10])
     attr6[n,t] <- (beta[1,11] * X[n,t,11] + beta[1,12] * X[n,t,12])
     attr7[n,t] <- (beta[1,13] * X[n,t,13] + beta[1,14] * X[n,t,14])
     attr8[n,t] <- (beta[1,15] * X[n,t,15] + beta[1,16] * X[n,t,16])
 }}

 # utility function P2
 for (n in N1+1:N1+N2){
   for (t in 1:T){
     phi[n,t]  <- (attr1[n,t] + attr2[n,t] + attr3[n,t] + attr4[n,t] + attr5[n,t] +
                  attr6[n,t] + attr7[n,t] + attr8[n,t]) * gmnl_scale[n]
     attr1[n,t] <- (beta[2,1] * X[n,t,1]  + beta[2,2] * X[n,t,2] )
     attr2[n,t] <- (beta[2,3] * X[n,t,3]  + beta[2,4] * X[n,t,4] )
     attr3[n,t] <- (beta[2,5] * X[n,t,5]  + beta[2,6] * X[n,t,6] )
     attr4[n,t] <- (beta[2,7] * X[n,t,7]  + beta[2,8] * X[n,t,8] )
     attr5[n,t] <- (beta[2,9] * X[n,t,9]  + beta[2,10]* X[n,t,10])
     attr6[n,t] <- (beta[2,11]* X[n,t,11] + beta[2,12]* X[n,t,12])
     attr7[n,t] <- (beta[2,13]* X[n,t,13] + beta[2,14]* X[n,t,14])
     attr8[n,t] <- (beta[2,15]* X[n,t,15] + beta[2,16]* X[n,t,16])
 }}


 # utility function P3
 for (n in N1+N2+1:N1+N2+N3){
   for (t in 1:T){
     phi[n,t]  <- (attr1[n,t] + attr2[n,t] + attr3[n,t] + attr4[n,t] + attr5[n,t] +
                  attr6[n,t] + attr7[n,t] + attr8[n,t]) * gmnl_scale[n]
     attr1[n,t] <- (beta[3,1] * X[n,t,1]  + beta[3,2] * X[n,t,2] )
     attr2[n,t] <- (beta[3,3] * X[n,t,3]  + beta[3,4] * X[n,t,4] )
     attr3[n,t] <- (beta[3,5] * X[n,t,5]  + beta[3,6] * X[n,t,6] )
     attr4[n,t] <- (beta[3,7] * X[n,t,7]  + beta[3,8] * X[n,t,8] )
     attr5[n,t] <- (beta[3,9] * X[n,t,9]  + beta[3,10]* X[n,t,10])
```


For peer review only

For peer review only - http://bmjopen.bmj.com/site/about/guidelines.xhtml

```
    attr6[n,t] <- (beta[3,11]* X[n,t,11] + beta[3,12]* X[n,t,12])
    attr7[n,t] <- (beta[3,13]* X[n,t,13] + beta[3,14]* X[n,t,14])
    attr8[n,t] <- (beta[3,15]* X[n,t,15] + beta[3,16]* X[n,t,16])
}}

# utility function P4
for (n in N1+N2+N3+1:N1+N2+N3+N4){
  for (t in 1:T){
    phi[n,t]  <- (attr1[n,t] + attr2[n,t] + attr3[n,t] + attr4[n,t] + attr5[n,t] +
                  attr6[n,t] + attr7[n,t] + attr8[n,t]) * gmnl_scale[n]
    attr1[n,t] <- (beta[4,1] * X[n,t,1]  + beta[4,2] * X[n,t,2] )
    attr2[n,t] <- (beta[4,3] * X[n,t,3]  + beta[4,4] * X[n,t,4] )
    attr3[n,t] <- (beta[4,5] * X[n,t,5]  + beta[4,6] * X[n,t,6] )
    attr4[n,t] <- (beta[4,7] * X[n,t,7]  + beta[4,8] * X[n,t,8] )
    attr5[n,t] <- (beta[4,9] * X[n,t,9]  + beta[4,10]* X[n,t,10])
    attr6[n,t] <- (beta[4,11]* X[n,t,11] + beta[4,12]* X[n,t,12])
    attr7[n,t] <- (beta[4,13]* X[n,t,13] + beta[4,14]* X[n,t,14])
    attr8[n,t] <- (beta[4,15]* X[n,t,15] + beta[4,16]* X[n,t,16])
}}

# priors on beta's
beta[1,1:V] ~ dmnorm(zeros[], tau_beta[,])
beta[2,1:V] ~ dmnorm(zeros[], tau_beta[,])
beta[3,1:V] ~ dmnorm(zeros[], tau_beta[,])
beta[4,1:V] ~ dmnorm(zeros[], tau_beta[,])

for (b in 1:V){
 zeros[b] <- 0
 for (bb in 1:V){
   tau_beta[b,bb] <- equals(b,bb)/100
}}

# priors on gmnl_scale parameters
# subject to a mean-of-one constraint for identification
gmnl_scale[1:N1] ~ dGMNLscale(tau[1])
gmnl_scale[N1+1:N1+N2] ~ dGMNLscale(tau[2])
gmnl_scale[N1+N2+1:N1+N2+N3] ~ dGMNLscale(tau[3])
gmnl_scale[N1+N2+N3+1:N1+N2+N3+N4] ~ dGMNLscale(tau[4])
# this implies that SD(tau) is restricted between 0.01 and 10
for (group in 1:4){
  tau[group] ~ dunif(0.01,10000)
}

# monitor SD of gmnl_scale parameters
for (group in 1:4){
  gmnl_SD[group] <- sqrt( 1.0/ tau[group] )
}

# monitor attribute importances
for (group in 1:4){
  weight[group,1] <- beta[group,2]
  weight[group,2] <- beta[group,4]
  weight[group,3] <- beta[group,6]
  weight[group,4] <- beta[group,8]
  weight[group,5] <- beta[group,10]
  weight[group,6] <- beta[group,12]
  weight[group,7] <- beta[group,14]
  weight[group,8] <- beta[group,16]
  total_weight[group] <- sum(weight[group,])
  attr_importance[group,1] <- weight[group,1] / total_weight[group]
  attr_importance[group,2] <- weight[group,2] / total_weight[group]
  attr_importance[group,3] <- weight[group,3] / total_weight[group]
  attr_importance[group,4] <- weight[group,4] / total_weight[group]
  attr_importance[group,5] <- weight[group,5] / total_weight[group]
  attr_importance[group,6] <- weight[group,6] / total_weight[group]
  attr_importance[group,7] <- weight[group,7] / total_weight[group]
  attr_importance[group,8] <- weight[group,8] / total_weight[group]
 }
}
```


For peer review only - http://bmjopen.bmj.com/site/about/guidelines.xhtml

Model #2. Combined S-MNL model with multiplicative attribute-importance parameters

```
model {

 # likelihood of pairwise choice tasks
 for (n in 1:N){
   for(t in 1:T){
     Y[n,t] <- 0
     Y[n,t] ~ dcat2(phi[n,t])
 }}

 # utility function P1
 for (n in 1:N1){
   for (t in 1:T){
     phi[n,t]  <- (attr1[n,t] + attr2[n,t] + attr3[n,t] + attr4[n,t] + attr5[n,t] +
                    attr6[n,t] + attr7[n,t] + attr8[n,t]) * gmnl_scale[n] * scale[1]
     attr1[n,t] <- (beta[1]  * X[n,t,1]  + beta[2]  * X[n,t,2] )
     attr2[n,t] <- (beta[3]  * X[n,t,3]  + beta[4]  * X[n,t,4] )
     attr3[n,t] <- (beta[5]  * X[n,t,5]  + beta[6]  * X[n,t,6] )
     attr4[n,t] <- (beta[7]  * X[n,t,7]  + beta[8]  * X[n,t,8] )
     attr5[n,t] <- (beta[9]  * X[n,t,9]  + beta[10] * X[n,t,10])
     attr6[n,t] <- (beta[11] * X[n,t,11] + beta[12] * X[n,t,12])
     attr7[n,t] <- (beta[13] * X[n,t,13] + beta[14] * X[n,t,14])
     attr8[n,t] <- (beta[15] * X[n,t,15] + beta[16] * X[n,t,16])
 }}

 # utility function P2
 for (n in N1+1:N1+N2){
   for (t in 1:T){
     phi[n,t]  <- (attr1[n,t] + attr2[n,t] + attr3[n,t] + attr4[n,t] + attr5[n,t] +
                    attr6[n,t] + attr7[n,t] + attr8[n,t]) * gmnl_scale[n] * scale[2]
     attr1[n,t] <- (beta[1] * X[n,t,1]  + beta[2] * X[n,t,2] ) * rho[2,1]
     attr2[n,t] <- (beta[3] * X[n,t,3]  + beta[4] * X[n,t,4] ) * rho[2,2]
     attr3[n,t] <- (beta[5] * X[n,t,5]  + beta[6] * X[n,t,6] ) * rho[2,3]
     attr4[n,t] <- (beta[7] * X[n,t,7]  + beta[8] * X[n,t,8] ) * rho[2,4]
     attr5[n,t] <- (beta[9] * X[n,t,9]  + beta[10]* X[n,t,10]) * rho[2,5]
     attr6[n,t] <- (beta[11]* X[n,t,11] + beta[12]* X[n,t,12]) * rho[2,6]
     attr7[n,t] <- (beta[13]* X[n,t,13] + beta[14]* X[n,t,14]) * rho[2,7]
     attr8[n,t] <- (beta[15]* X[n,t,15] + beta[16]* X[n,t,16]) * rho[2,8]
 }}


 # utility function P3
 for (n in N1+N2+1:N1+N2+N3){
   for (t in 1:T){
     phi[n,t]  <- (attr1[n,t] + attr2[n,t] + attr3[n,t] + attr4[n,t] + attr5[n,t] +
                    attr6[n,t] + attr7[n,t] + attr8[n,t]) * gmnl_scale[n] * scale[3]
     attr1[n,t] <- (beta[1] * X[n,t,1]  + beta[2] * X[n,t,2] ) * rho[3,1]
     attr2[n,t] <- (beta[3] * X[n,t,3]  + beta[4] * X[n,t,4] ) * rho[3,2]
     attr3[n,t] <- (beta[5] * X[n,t,5]  + beta[6] * X[n,t,6] ) * rho[3,3]
     attr4[n,t] <- (beta[7] * X[n,t,7]  + beta[8] * X[n,t,8] ) * rho[3,4]
     attr5[n,t] <- (beta[9] * X[n,t,9]  + beta[10]* X[n,t,10]) * rho[3,5]
     attr6[n,t] <- (beta[11]* X[n,t,11] + beta[12]* X[n,t,12]) * rho[3,6]
     attr7[n,t] <- (beta[13]* X[n,t,13] + beta[14]* X[n,t,14]) * rho[3,7]
     attr8[n,t] <- (beta[15]* X[n,t,15] + beta[16]* X[n,t,16]) * rho[3,8]
 }}

 # utility function P4
 for (n in N1+N2+N3+1:N1+N2+N3+N4){
   for (t in 1:T){
     phi[n,t]  <- (attr1[n,t] + attr2[n,t] + attr3[n,t] + attr4[n,t] + attr5[n,t] +
                    attr6[n,t] + attr7[n,t] + attr8[n,t]) * gmnl_scale[n] * scale[4]
     attr1[n,t] <- (beta[1] * X[n,t,1]  + beta[2] * X[n,t,2] ) * rho[4,1]
     attr2[n,t] <- (beta[3] * X[n,t,3]  + beta[4] * X[n,t,4] ) * rho[4,2]
     attr3[n,t] <- (beta[5] * X[n,t,5]  + beta[6] * X[n,t,6] ) * rho[4,3]
     attr4[n,t] <- (beta[7] * X[n,t,7]  + beta[8] * X[n,t,8] ) * rho[4,4]
     attr5[n,t] <- (beta[9] * X[n,t,9]  + beta[10]* X[n,t,10]) * rho[4,5]
     attr6[n,t] <- (beta[11]* X[n,t,11] + beta[12]* X[n,t,12]) * rho[4,6]
     attr7[n,t] <- (beta[13]* X[n,t,13] + beta[14]* X[n,t,14]) * rho[4,7]
     attr8[n,t] <- (beta[15]* X[n,t,15] + beta[16]* X[n,t,16]) * rho[4,8]
 }}

 # priors on beta's
 beta[1:V] ~ dmnorm(zeros[], tau_beta[,])

 for (b in 1:V){
  zeros[b] <- 0
  for (bb in 1:V){
    tau_beta[b,bb] <- equals(b,bb)/100
 }}
```


For peer review only

```
# priors on gmnl_scale parameters
# subject to a mean-of-one constraint for identification
gmnl_scale[1:N1] ~ dGMNLscale(tau[1])
gmnl_scale[N1+1:N1+N2] ~ dGMNLscale(tau[2])
gmnl_scale[N1+N2+1:N1+N2+N3] ~ dGMNLscale(tau[3])
gmnl_scale[N1+N2+N3+1:N1+N2+N3+N4] ~ dGMNLscale(tau[4])
# this implies that SD(tau) is restricted between 0.01 and 10
for (group in 1:4){
  tau[group] ~ dunif(0.01,10000)
}

# monitor SD of gmnl_scale parameters
for (group in 1:4){
  gmnl_SD[group] <- sqrt( 1.0/ tau[group] )
}

# priors on relative attribute importance parameters (P1 is the base-case sample)
# subject to a mean-of-one constraint for identification
# see Jonker et al. 2019, this prior places 95% of the prior mass between 0.4 and 2.0
 for (att in 1:8){
  rho[1,att] <- 1
}
for (group in 2:4){
  rho[group,1:8] ~ dscale(6.25)
}

# priors on the relative scale parameters (P1 is the base-case sample)
# see Jonker et al. 2019, this prior places 95% of the prior mass between 0.4 and 2.0
scale[1] <- 1
for (group in 2:4) {
  scale[group] ~ dlnorm(-0.08,6.25)C(0.1,10)
}
# the truncation normalizing constant in the log prior does not need to be explicitly
# evaluated since the truncation boundaries remain constant during MCMC sampling. Hence
# sensoring C() is sufficient and can be used instead of truncation T(), which isn't implemented


# monitor attribute importances
for (group in 1:4){
  weight[group,1] <- beta[2]  * rho[group,1]
  weight[group,2] <- beta[4]  * rho[group,2]
  weight[group,3] <- beta[6]  * rho[group,3]
  weight[group,4] <- beta[8]  * rho[group,4]
  weight[group,5] <- beta[10] * rho[group,5]
  weight[group,6] <- beta[12] * rho[group,6]
  weight[group,7] <- beta[14] * rho[group,7]
  weight[group,8] <- beta[16] * rho[group,8]
  total_weight[group] <- sum(weight[group,])
  attr_importance[group,1] <- weight[group,1] / total_weight[group]
  attr_importance[group,2] <- weight[group,2] / total_weight[group]
  attr_importance[group,3] <- weight[group,3] / total_weight[group]
  attr_importance[group,4] <- weight[group,4] / total_weight[group]
  attr_importance[group,5] <- weight[group,5] / total_weight[group]
  attr_importance[group,6] <- weight[group,6] / total_weight[group]
  attr_importance[group,7] <- weight[group,7] / total_weight[group]
  attr_importance[group,8] <- weight[group,8] / total_weight[group]
 }
}
```

For peer review only

Box S3. Indicators of questionnaire completion and perceived difficulty

**Completion**	**AT**	**HR**	**HU**	**DE**	**NL**	**NO**	**ES**	**UK**
Gave informed consent	253	229	283	207	207	203	150	208
Finished all DCE scenario's (%)	170 (67)	173 (76)	194 (93)	166 (80)	160 (77)	158 (78)	150 (100)	165 (79)
Finished entire questionnaire	169 (68)	168 (73)	192 (68)	160 (77)	156 (75)	156 (77)	150 (100)	163 (78)
Mean (SD) time to completion entire questionnaire	22.87 (21.73)	22.43 (37.68)	20.27 (14.88)	26.21 (48.74)	26.21 (48.74)	27.33 (27.90)	26.96 (10.12)	24.68 (62.64)
Required >5 minutes (%)*	167 (99)	160 (95)	180 (94)	158 (99)	156 (100)	152 (97)	149 (99)	155 (95)
**Self-reported difficulty**								
Very easy	16 (9.5)	11 (6.5)	23 (12.0)	14 (8.8)	3 (1.9)	2 (1.3)	1 (0.7)	31 (19.0)
Easy	39 (23.1)	30 (17.9)	71 (37.0)	33 (20.6)	40 (25.6)	20 (12.8)	36 (24.0)	45 (27.6)
Not too easy, not too difficult	85 (50.3)	70 (41.7)	75 (39.1)	84 (52.5)	74 (47.4)	79 (50.6)	71 (47.3)	63 (38.7)
Difficult	25 (14.8)	44 (26.2)	22 (11.5)	24 (15.0)	31 (19.9)	48 (30.8)	30 (20.0)	22 (13.5)
Very difficult	4 (2.4)	13 (7.7)	1 (0.5)	5 (3.1)	8 (5.1)	7 (4.5)	12 (8.0)	2 (1.2)

* As % of those that completed the entire questionnaire

For peer review only

Box S4. Patients' self-reported health problems.

N (%)	AT	HR	HU	DE	NL	NO	ES	UK
I prefer not to answer	4 (2.4)	10 (6.0)	3 (1.6)	4 (1.9)	2 (1.3)	0 (0)	6 (4)	3 (1.8)
Asthma	20 (11.8)	19 (11.3)	26 (13.5)	18 (8.7)	19 (12.2)	29 (18.6)	6 (4)	41 (25.2)
Cancer during the past five years	10 (5.9)	5 (3.0)	11 (5.7)	4 (1.9)	10 (6.4)	11 (7.1)	18 (12)	10 (6.1)
Chronic back pain or sciatica	69 (40.8)	54 (32.1)	32 (16.7)	47 (22.6)	32 (20.5)	29 (18.6)	50 (33.3)	51 (31.3)
Chronic bronchitis, COPD or emphysema	32 (18.9)	8 (4.8)	9 (4.7)	14 (6.7)	26 (16.7)	7 (4.5)	29 (19.3)	28 (17.2)
Chronic kidney disease	4 (2.4)	6 (3.6)	8 (4.2)	9 (4.3)	4 (2.6)	0 (0)	9 (6)	8 (4.9)
Cognitive problems, memory loss, dementia, Alzheimer's	3 (1.8)	0 (0)	2 (1.0)	2 (1.0)	6 (3.8)	6 (3.9)	0 (0)	4 (2.5)
Colon problem, irritable bowel or colitis	28 (16.6)	18 (10.7)	15 (7.8)	9 (4.3)	34 (21.8)	31 (19.9)	7 (4.7)	32 (19.6)
Congestive heart failure	9 (5.3)	1 (0.6)	4 (2.2)	8 (3.8)	9 (5.8)	5 (3.2)	9 (6)	3 (1.8)
Depression, anxiety or emotional difficulties	63 (37.3)	47 (28.0)	63 (32.8)	36 (17.3)	29 (18.6)	32 (20.5)	36 (24)	70 (42.9)
Diabetes	29 (17.2)	17 (10.1)	43 (22.4)	35 (16.8)	36 (23.1)	16 (10.3)	26 (17.3)	45 (27.6)
Hard of hearing	18 (10.7)	16 (9.5)	27 (14.1)	14 (6.7)	28 (17.9)	35 (22.4)	17 (11.3)	18 (11.0)
Heart disease, angina (chest pain from heart problem), heart attack, bypass surgery or angioplasty	20 (11.8)	19 (11.3)	20 (10.4)	10 (4.8)	26 (16.7)	15 (9.6)	19 (12.7)	28 (17.2)
High blood pressure	67 (39.6)	40 (23.8)	95 (49.5)	68 (32.7)	61 (39.1)	58 (37.2)	50 (33.3)	64 (39.3)
High cholesterol	34 (20.1)	27 (16.1)	46 (24.0)	40 (19.2)	47 (30.1)	38 (24.4)	45 (30)	47 (28.8)
Osteoarthritis (not rheumatoid arthritis)	5 (3.0)	9 (5.4)	59 (30.7)	7 (3.4)	33 (21.2)	46 (29.5)	7 (4.7)	43 (26.4)
Osteoporosis (brittle bones)	15 (8.9)	11 (6.5)	13 (6.8)	6 (2.9)	10 (6.4)	9 (5.8)	21 (14)	16 (9.8)
Overweight	55 (32.5)	46 (27.4)	68 (35.4)	56 (26.9)	49 (31.4)	51 (32.7)	47 (31.3)	47 (28.8)
Poor circulation in your legs	22 (13.0)	27 (16.1)	49 (25.5)	17 (8.2)	21 (13.5)	22 (14.1)	35 (23.3)	23 (14.1)
Rheumatoid arthritis	25 (14.8)	10 (6.0)	19 (9.9)	17 (8.2)	21 (13.5)	10 (6.4)	12 (8)	19 (11.7)

For peer review only - http://bmjopen.bmj.com/site/about/guidelines.xhtml

Rheumatic disease, fibromyalgia or lupus	14 (8.3)	5 (3.0)	13 (6.8)	15 (7.2)	11 (7.1)	16 (10.3)	12 (8)	11 (6.7)
Stomach problem, ulcer, gastritis or reflux	29 (17.2)	27 (16.1)	46 (24.0)	25 (12.0)	21 (13.5)	24 (15.4)	22 (14.7)	28 (17.2)
Stroke	4 (2.4)	4 (2.4)	8 (4.2)	10 (4.8)	3 (1.9)	2 (1.3)	7 (4.7)	9 (5.5)
Thyroid disorder	30 (17.8)	33 (19.6)	23 (12.0)	27 (13.0)	14 (9.0)	18 (11.5)	10 (6.7)	16 (9.8)
Vision problem	26 (15.4)	50 (29.8)	28 (14.6)	29 (13.9)	28 (17.9)	21 (13.5)	56 (37.3)	18 (11.0)
No health problems	1 (0.6)	6 (3.6)	0 (0.0)	28 (13.5)	2 (1.3)	0 (0)	3 (2)	0 (0)
Other health problems	39 (23.1)	30 (17.9)	31 (16.2)	16 (7.7)	39 (25.0)	34 (21.8)	31 (20.7)	73 (44.8)

For peer review only

For peer review only - http://bmjopen.bmj.com/site/about/guidelines.xhtml

Supplementary file Box S5. Coefficients and 95% credibility intervals (CI) of the multiplicative interaction terms to investigate differences between patients (P1) and the other stakeholder groups, i.e., Partners & other informal caregivers (P2), professionals (P3), payers (P4), and policy makers (P5). The bold cells indicate the 95% CI of the multiplicative interaction terms for stakeholder group and attribute that excluded 1.

		**P2 vs P1**			**P3 vs P1**			**P4/5 vs P1**			**P4 vs P1**			**P5 vs P1**		
		**coefficient**	**95% CI**		**coefficient**	**95% CI**		**coefficient**	**95% CI**		**coefficient**	**95% CI**		**coefficient**	**95% CI**	
**AT**	Physical functioning	0.74	0.52	**1.00**	0.64	0.48	**0.82**	0.65	0.48	**0.87**						
	Psych.l well-being	1.25	0.95	1.62	1.14	0.91	1.42	1.06	0.82	1.35						
	Social relations & part	0.93	0.62	1.29	1.16	0.92	1.45	1.09	0.83	1.40						
	Enjoyment of life	1.11	0.88	1.38	0.94	0.78	1.11	0.89	0.72	1.09						
	Resilience	0.98	0.74	1.24	0.87	0.70	1.07	0.79	0.61	1.00						
	Person-centeredness	1.27	0.79	1.83	1.45	**1.07**	1.88	1.16	0.79	1.58						
	Continuity of care	1.01	0.76	1.30	1.17	0.94	1.41	1.07	0.83	1.34						
	Total costs	0.71	0.40	1.10	0.64	0.39	**0.97**	1.29	0.87	1.83						
**DE**	Physical functioning	0.56	0.34	**0.83**	0.52	0.38	**0.69**	0.77	0.57	1.00						
	Psychological well-being	0.81	0.50	1.22	1.04	0.77	1.40	1.19	0.88	1.58						
	Social relations & part	0.98	0.63	1.40	1.01	0.77	1.31	0.88	0.64	1.16						
	Enjoyment of life	1.07	0.84	1.35	0.80	0.66	**0.96**	0.78	0.63	**0.96**						
	Resilience	1.00	0.74	1.29	0.78	0.63	**0.96**	0.73	0.57	**0.91**						
	Person-centeredness	1.09	0.66	1.64	1.70	**1.28**	2.17	1.17	0.82	1.59						
	Continuity of care	1.32	1.00	1.69	1.19	0.98	1.44	0.89	0.69	1.12						
	Total costs	1.16	0.66	1.72	0.95	0.65	1.36	1.59	**1.15**	2.15						
**ES**	Physical functioning	1.23	**1.06**	1.44	0.51	0.41	**0.64**									
	Psychological well-being	0.77	0.64	**0.91**	0.73	0.60	**0.87**									
	Social relations & part	1.01	0.84	1.20	0.73	0.60	**0.88**									
	Enjoyment of life	0.87	0.72	1.03	0.96	0.80	1.13									
	Resilience	1.01	0.83	1.22	0.80	0.65	**0.99**									
	Person-centeredness	1.43	**1.16**	1.75	1.64	**1.35**	1.96									
	Continuity of care	0.95	0.70	1.25	1.12	0.86	1.45									
	Total costs	0.73	0.43	1.11	1.50	**1.06**	2.09									

For peer review only - http://bmjopen.bmj.com/site/about/guidelines.xhtml

**HR**	Physical functioning	0.87	0.62	1.16	0.86	0.59	1.14	1.20	0.89	1.55						
	Psychological well-being	1.04	0.83	1.30	1.07	0.84	1.33	0.83	0.62	1.06						
	Social relations & part	0.92	0.70	1.17	1.00	0.77	1.26	0.91	0.67	1.17						
	Enjoyment of life	1.01	0.83	1.21	1.22	**1.02**	1.45	1.01	0.82	1.23						
	Resilience	0.81	0.63	1.01	0.81	0.63	1.01	1.03	0.81	1.29						
	Person-centeredness	1.14	0.91	1.39	1.08	0.86	1.33	0.95	0.73	1.19						
	Continuity of care	1.20	0.98	1.44	1.26	**1.03**	1.51	0.87	0.68	1.10						
	Total costs	1.00	0.55	1.59	0.70	0.36	1.18	1.20	0.67	1.91						
**HU**	Physical functioning	0.77	0.53	1.06	0.89	0.67	1.16	0.87	0.67	1.10						
	Psychological well-being	1.57	**1.14**	2.08	1.16	0.84	1.55	1.16	0.87	1.52						
	Social relations & part	1.01	0.70	1.35	0.93	0.69	1.22	1.05	0.82	1.32						
	Enjoyment of life	1.30	0.98	1.66	1.14	0.89	1.42	1.04	0.84	1.29						
	Resilience	0.80	0.57	1.08	0.77	0.58	1.01	0.65	0.50	**0.84**						
	Person-centeredness	0.90	0.64	1.19	1.32	**1.04**	1.66	1.01	0.79	1.25						
	Continuity of care	1.05	0.83	1.30	0.95	0.77	1.15	0.81	0.66	**0.97**						
	Total costs	0.60	0.29	1.07	0.83	0.44	1.32	1.42	0.91	2.09						
**NL**	Physical functioning	0.77	0.63	**0.92**	0.73	0.61	**0.87**				0.92	0.79	1.06	0.87	0.74	1.00
	Psychological well-being	0.91	0.74	1.12	1.10	0.93	1.32				1.12	0.94	1.34	0.91	0.77	1.09
	Social relations & part	0.97	0.76	1.22	1.15	0.96	1.38				1.09	0.90	1.32	1.09	0.91	1.30
	Enjoyment of life	1.11	0.96	1.27	0.99	0.87	1.13				1.05	0.92	1.20	0.96	0.85	1.09
	Resilience	0.95	0.81	1.11	0.83	0.71	**0.96**				0.77	0.65	**0.89**	0.93	0.81	1.06
	Person-centeredness	1.05	0.81	1.33	1.02	0.82	1.26				0.80	0.61	1.01	0.98	0.78	1.21
	Continuity of care	1.07	0.85	1.31	0.91	0.72	1.10				0.73	0.56	**0.91**	0.82	0.66	1.00
	Total costs	1.16	0.81	1.55	1.27	0.96	1.62				1.53	**1.19**	1.93	1.43	**1.12**	1.82
**NO**	Physical functioning	0.66	0.53	**0.80**	0.71	0.60	**0.83**				0.77	0.64	**0.90**	0.63	0.53	**0.75**
	Psychological well-being	1.08	0.90	1.27	0.98	0.84	1.14				0.87	0.72	1.03	0.86	0.73	1.01

For peer review only - http://bmjopen.bmj.com/site/about/guidelines.xhtml

	Social relations & part	1.32	**1.09**	1.56	1.19	**1.01**	1.40				0.97	0.79	1.17	1.18	0.98	1.39
	Enjoyment of life	1.12	0.94	1.32	1.11	0.94	1.29				1.04	0.87	1.23	0.99	0.84	1.15
	Resilience	0.83	0.64	1.06	1.00	0.84	1.19				1.09	0.90	1.32	1.07	0.88	1.29
	Person-centeredness	1.25	0.86	1.66	1.08	0.79	1.41				0.79	0.51	1.09	1.10	0.81	1.44
	Continuity of care	0.86	0.67	1.09	0.92	0.74	1.12				0.72	0.57	**0.92**	1.03	0.84	1.26
	Total costs	0.88	0.46	1.41	1.02	0.62	1.48				1.77	**1.25**	2.36	1.13	0.71	1.66
**UK**	Physical functioning	0.82	0.63	1.03	0.94	0.77	1.14	0.94	0.76	1.15						
	Psychological well-being	1.17	0.95	1.42	1.00	0.82	1.20	1.07	0.85	1.31						
	Social relations & part	1.06	0.84	1.29	1.03	0.83	1.25	1.01	0.79	1.25						
	Enjoyment of life	1.15	0.97	1.34	0.98	0.83	1.14	0.94	0.79	1.12						
	Resilience	1.01	0.79	1.26	0.97	0.78	1.17	1.04	0.83	1.29						
	Person-centeredness	1.06	0.79	1.34	1.11	0.87	1.39	1.18	0.90	1.50						
	Continuity of care	0.85	0.65	1.09	0.87	0.68	1.09	0.99	0.77	1.24						
	Total costs	0.89	0.57	1.24	1.11	0.81	1.48	0.82	0.52	1.17						

For peer review only

For peer review only - http://bmjopen.bmj.com/site/about/guidelines.xhtml

For peer review only

For peer review only - http://bmjopen.bmj.com/site/about/guidelines.xhtml

BMJ Open

Comparing patients' and other stakeholders' preferences for outcomes of integrated care for multimorbidity: a discrete choice experiment in eight European countries.

Journal:	*BMJ Open*
Manuscript ID	bmjopen-2020-037547.R1
Article Type:	Original research
Date Submitted by the Author:	22-Jun-2020
Complete List of Authors:	Rutten-van Molken, Maureen; Erasmus Universiteit Rotterdam, Erasmus School of Health Policy & Management<br>Karimi, Milad; Erasmus Universiteit Rotterdam, Erasmus School of Health Policy & Management<br>Leijten, Fenna; Erasmus Universiteit Rotterdam, Erasmus School of Health Policy & Management ; Ministry of defence, Staff Defence Healthcare Organisation<br>Hoedemakers, Maaike; Erasmus Universiteit Rotterdam, Erasmus School of Health Policy & Management<br>Looman, Willemijn; Erasmus Universiteit Rotterdam, Erasmus School of Health Policy & Management<br>Islam, Kamrul; University of Bergen, Department of Economics<br>Askildsen, Jan; University of Bergen, Department of Economics<br>Kraus, Markus; Institut fur Hohere Studien,<br>Ercevic, Darija; Agency for Quality and Accreditation in Health Care and Social Welfare<br>Struckmann, Verena; Berlin University of Technology, Department of Health Care Management<br>Pitter, Janos; Syreon Research Institute<br>Cano, Isaac; Hospital Clinic de Barcelona, Institut d'Investigacions Biomèdiques August Pi i Sunyer (IDIBAPS)<br>Stokes, Jonathan; University of Manchester Institute of Population Health, Centre for Health Economics<br>Jonker, Marcel; Erasmus Universiteit Rotterdam, Health Policy & Management
**Primary Subject Heading**:	Health services research
Secondary Subject Heading:	Patient-centred medicine, Health economics, Health policy
Keywords:	PRIMARY CARE, HEALTH ECONOMICS, International health services < HEALTH SERVICES ADMINISTRATION & MANAGEMENT, Organisation of health services < HEALTH SERVICES ADMINISTRATION & MANAGEMENT, PUBLIC HEALTH

For peer review only - http://bmjopen.bmj.com/site/about/guidelines.xhtml

I, the Submitting Author has the right to grant and does grant on behalf of all authors of the Work (as defined in the below author licence), an exclusive licence and/or a non-exclusive licence for contributions from authors who are: i) UK Crown employees; ii) where BMJ has agreed a CC-BY licence shall apply, and/or iii) in accordance with the terms applicable for US Federal Government officers or employees acting as part of their official duties; on a worldwide, perpetual, irrevocable, royalty-free basis to BMJ Publishing Group Ltd ("BMJ") its licensees and where the relevant Journal is co-owned by BMJ to the co-owners of the Journal, to publish the Work in this journal and any other BMJ products and to exploit all rights, as set out in our licence.

The Submitting Author accepts and understands that any supply made under these terms is made by BMJ to the Submitting Author unless you are acting as an employee on behalf of your employer or a postgraduate student of an affiliated institution which is paying any applicable article publishing charge ("APC") for Open Access articles. Where the Submitting Author wishes to make the Work available on an Open Access basis (and intends to pay the relevant APC), the terms of reuse of such Open Access shall be governed by a Creative Commons licence – details of these licences and which Creative Commons licence will apply to this Work are set out in our licence referred to above.

Other than as permitted in any relevant BMJ Author's Self Archiving Policies, I confirm this Work has not been accepted for publication elsewhere, is not being considered for publication elsewhere and does not duplicate material already published. I confirm all authors consent to publication of this Work and authorise the granting of this licence.

For peer review only

Comparing patients' and other stakeholders' preferences for outcomes of integrated care for multimorbidity: a discrete choice experiment in eight European countries.

Maureen Rutten-van Mölken[1,2], Milad Karimi[1], Fenna Leijten,[1,3] Maaike Hoedemakers[1], Willemijn Looman[1], Kamrul Islam[4], Jan Erik Askildsen[4], Markus Kraus[5], Darija Ercevic[6], Verena Struckmann[7], Janos Gyorgy Pitter[8], Isaac Cano[9], Jonathan Stokes[10], Marcel Jonker[1], on behalf of the SELFIE consortium

[1] School of Health Policy and Management, Erasmus University Rotterdam, Rotterdam, the Netherlands,

[2] Institute for Medical Technology Assessment, Erasmus University Rotterdam, Rotterdam, the Netherlands

[3] Staff Defence Healthcare Organisation, Dutch Ministry of Defence, the Netherlands

[4] Department of Economics, University of Bergen, Bergen, Norway

[5] Institute for Advanced Studies, Vienna, Austria

[6] Agency for Quality and Accreditation in Health Care and Social Welfare, Zagreb, Croatia

[7] Department of Health Care Management, Technische Universität Berlin, Berlin, Germany

[8] Syreon Research Institute, Budapest, Hungary

[9] Hospital Clinic de Barcelona, Institut d'Investigacions Biomèdiques August Pi i Sunyer (IDIBAPS), Universitat de Barcelona. Barcelona, Spain

[10] Centre for Primary Care and Health Services Research, University of Manchester, Manchester, United Kingdom

Email corresponding author Maureen Rutten-van Mölken: m.rutten@eshpm.eur.nl (MRvM)

ORCID-ID: 0000-0001-8706-3159

Keywords: multimorbidity, preferences, discrete choice experiment, integrated care, Triple Aim, informal care, policy making, SELFIE, Multi-Criteria Decision Analysis, Europe

Target journal: BMJ; Word count 4628

For peer review only

For peer review only - http://bmjopen.bmj.com/site/about/guidelines.xhtml

Abstract (word count 300)

Objectives: To measure relative preferences for outcomes of integrated care of patients with multimorbidity from eight European countries and compare them to the preferences of other stakeholders within these countries.

Design: A discrete choice experiment (DCE) was conducted in each country, asking respondents to choose between two integrated care programmes for persons with multimorbidity.

Setting: Preference data collected in Austria(AT), Croatia(HR), Germany(DE), Hungary(HU), Netherlands(NL), Norway(NO), United Kingdom(UK).

Participants: Patients with multimorbidity, partners & other informal caregivers, professionals, payers, policy makers.

Main outcome measures: Preferences of participants regarding outcomes of integrated care described as health/wellbeing, experience with care, and cost outcomes, i.e., physical functioning, psychological wellbeing, social relationships & participation, enjoyment of life, resilience, person-centeredness, continuity of care, total costs. Each outcome had three levels of performance.

Results: 5122 respondents completed the DCE. In all countries, patients with multimorbidity, as well as most other stakeholder groups, assigned the (second) highest preference to enjoyment of life. The patients top-three most frequently included physical functioning, psychological wellbeing, and continuity of care. Continuity of care also entered the top-three of professionals, payers and policy makers in four countries (AT, DE, HR, HU). Of the five stakeholder groups, preferences of professionals differed most often from preferences of patients. Professionals assigned lower weights to physical functioning in AT, DE, ES, NL, NO and higher weights to person-centeredness in AT, DE, ES, HU. Payers and policy makers assigned higher weights than patients to costs, but these weights were relatively low.

Conclusion: The wellbeing outcome enjoyment of life is the most important outcome of integrated care in multimorbidity. This calls for a greater involvement of social and mental care providers. The difference in opinion between patients and professionals calls for shared decision making, whereby efforts to improve wellbeing and person-centeredness should not divert attention from improving physical functioning.

For peer review only - http://bmjopen.bmj.com/site/about/guidelines.xhtml

Strengths and limitations

We designed an online DCE to measure the relative importance of health, well-being, experience, and cost outcomes of integrated care to patients with multimorbidity from eight European countries.

The choice of outcomes was largely driven by focus groups involving patients with multimorbidity.

We compared patients' preferences with the preferences of other stakeholders including informal caregivers, professionals, payers, and policy makers in a large sample of 5122 respondents.

Online discrete choice experiments are cognitively challenging and respondents may not represent all patients with multimorbidity.

When completing the DCE, it might be challenging for the other stakeholders to separate their professional role from the other roles they might have.

For peer review only

For peer review only - http://bmjopen.bmj.com/site/about/guidelines.xhtml

Introduction

Persons with multimorbidity may have complex care needs requiring the involvement of different types of professionals from different sectors of the healthcare, social care and/or community care systems. If the services provided by different professionals are not well aligned, they may experience conflicting treatment goals, harmful effects of interacting treatments, overly-demanding appeals on self-management capabilities, and inefficiencies like over- and under-treatment.

It is hypothesized, that persons with multimorbidity would benefit from person-centered integrated care programmes.[1-3] Integrated care programmes commonly adopt a holistic approach towards people with multimorbidity, which means that, instead of the disease, the person with his or her needs, capabilities, preferences, resources, and (social) environment is put in the centre.[4] In the EU-funded Horizon2020 project SELFIE (Sustainable intEgrated chronic care modeLs for multimorbidity: delivery, FInancing, and performancE), we study 17 promising integrated care programmes from eight European countries, i.e., Austria (AT), Croatia (HR), Germany (DE), Hungary (HU), the Netherlands (NL), Norway (NO), Spain (ES) and the United Kingdom (UK). In many of these programmes an interdisciplinary individual care plan, with well-aligned health and social care services, is designed to specifically target the goals and priorities jointly set by the person with multimorbidity and his or her formal and informal care givers. Many programmes also offer tailored self-management support and assign a case manager to the most complex patients, to ensure that they have a single contact point that helps them navigate through the system.

Integrated care programmes for persons with multimorbidity often aim to improve outcomes that go beyond health and include their broader sense of wellbeing, experience with care, and costs. This is often referred to as the Triple Aim of improving population health/wellbeing, experience and costs.[5] Therefore, we decided to measure a broad set of outcome measures in the evaluation studies of the 17 programmes in the SELFIE project. However, the importance of these outcomes is almost never measured. In the SELFIE project we were specifically interested in the importance of these outcomes to persons with multimorbidity. Understanding their preferences is vital for the optimal design of person-centered integrated care services and inform decision making on implementation and upscaling of these services. In the SELFIE project, we aim to use the preferences as weights in Multi-Criteria Decision Analyses (MCDA) of the 17 programmes.[6] In an MCDA, a wide range of different outcomes is aggregated into a single, overall value score. This overall value score combines the performance of the integrated care programmes on different outcomes, each weighted according to its respective preference weight.[7,8] Besides the persons with multi-morbidity, there are many other stakeholders involved in decision making about integrated care and their preferences may differ from patients' preferences. Therefore, we aim to repeat the MCDA's in the SELFIE project, using the preference weights from different groups of stakeholders.

The aim of the current paper is twofold. Firstly, to present the relative magnitude of the preferences for the Triple Aim outcomes in persons with multimorbidity from eight European countries. Secondly, the aim is to compare the <u>P</u>atients' preferences to the preferences of <u>P</u>artners & other informal caregivers, <u>P</u>rofessionals, <u>P</u>ayers and <u>P</u>olicy makers, together referred to as the 5 P's. This will be a within-country comparison, because the decision context of the future MCDA's relates to local or regional level decisions about the adaptation, reimbursement, continuation, and scaling-up of the 17 existing integrated care programmes.

For peer review only - http://bmjopen.bmj.com/site/about/guidelines.xhtml

Methods

Discrete choice experiment (DCE)

The preferences were obtained using discrete choice experiments (DCEs). A DCE is a theoretically well founded method to determine how much respondents are willing to trade-off some outcomes against others.[9-11] In our DCE, respondents from the 5P's were given questions that asked them to choose between two integrated care programmes for persons with multimorbidity, labelled programme A and programme B. There was no opt-out. Each programme was described in terms of outcomes with systematically varying levels that describe the performance of the programme on the outcomes. The outcomes were largely based on focus groups and included measures of health and wellbeing: 1) physical functioning, 2) psychological wellbeing, 3) social relationships & participation, 4) enjoyment of life, 5) resilience; measures of experience: 6) person-centeredness, 7) continuity of care; and 8) total health- and social care costs. The definitions are given in Table 1. An example of a DCE question is given in Figure 1.

(Table 1. Definitions of the outcomes and levels)

(Figure 1. Example of a DCE question)

Each outcome (attributes in DCE terminology) had three levels indicating a poor, average and good performance of the programme on that outcome. The definitions of the outcomes and levels were identical across the eight countries, except for costs. The three levels of costs were based on country-specific estimates of the mean total health and social care costs for people with multimorbidity in 2017 (middle level) and increased and decreased by 20% to obtain the poor and good performance level. This percentage was chosen to ensure enough variation in levels while at the same time remaining within realistic boundaries. The costs were expressed in the national currency.

DCE design

A Bayesian efficient heterogeneous design algorithm was used to optimise the DCE designs based on the D-efficiency criterion.[12] Ten different sub-designs with 18 choice tasks per sub-design were used. In each sub-design either four or five attribute levels were overlapped between programme A and B, to reduce the overall complexity of the choice tasks and improve response efficiency.[13] One restriction was built into the design, namely, the best level of the psychological wellbeing attribute and the worst level of enjoyment of life, and vice versa, were not presented simultaneously for the same programme.

The initial DCE designs were optimized using priors that were based on the available literature (See online supplementary file Box S1). These DCE designs were subsequently updated, based upon updated priors that reflected the information obtained from approximately the first 50 respondents in each stakeholder group. The design updates were performed separately for each country/stakeholder group and the updated priors were the average of the literature-based priors and the conditional logit estimates of the first 50 respondents.

For peer review only - http://bmjopen.bmj.com/site/about/guidelines.xhtml

Questionnaire design

The online DCE survey started with an explanation of the SELFIE project, the outcomes, type of questions, and the perspective from which the questions should be answered (i.e. one of the five P's). Each respondent completed a randomly chosen sub-design with 18 choice tasks, preceded by two practice-questions. The order of appearance of the choice tasks within each sub-design was randomised. The 18 choice tasks were presented in three sections of six choice tasks each, with several background questions in between. The background questions related to sociodemographic characteristics and the respondents' health conditions. Colour coding was used for the different levels of the outcomes as shown in Figure 1, and outcomes that had the same level in the two integrated care programmes were presented in grey.[13] When respondents hovered the mouse over an outcome, the definition of that outcome became visible. At the end of the survey, respondents received a multiple-choice question on the level of difficulty of the questionnaire.

The questionnaire was designed in English and translated into each country's respective language, using the same translation protocol, which included forward and backward translations by native-language speakers with an excellent level of English.

Respondents

Respondents from the stakeholder groups were recruited in the eight countries participating in the SELFIE project during 2017-2018. Sample size calculations[14] led to a target number of 150 respondents per country per stakeholder group. Each country recruited respondents from three to five different groups of stakeholders. All countries recruited respondents from the first three P's (i.e., patients with multimorbidity, partners & other informal caregivers, professionals). The Netherlands and Norway recruited respondents from all five P's. The other countries combined the respondents from the payers and policy makers into one group, either because they have a Beveridge-like healthcare model in which there is a national/regional health service with a single payer (HR, HU, UK, ES) or because they were in the midst of a comprehensive health insurance reform (AT) or an election (DE), making it difficult to recruit a sufficient number of respondents from the two groups separately.

Patients with multimorbidity and *partners & other informal caregivers* were recruited from internet panels managed by market research organisations, except in Spain where they were personally invited to participate by the members of the Spanish team of the SELFIE project who were physicians and nurses. In Spain, patients were recruited in the waiting rooms of a hospital, geriatric residences, and support groups for families with Alzheimer. All patients had two or more chronic conditions. Partners & other informal caregivers had to provide voluntary care or support to a family member, friend, or other acquaintance who needed help due to physical, mental or ageing-related health problems, for at least two weeks.

Professionals were also recruited through an internet panel organisation in the UK and the Netherlands. In the other countries, they were recruited through the networks of the authors and a snowball sampling method, in which we asked respondents if they could invite other responders within their organisations. Examples of organisations approached include care provider organisations or professional associations like nursing associations.

For peer review only - http://bmjopen.bmj.com/site/about/guidelines.xhtml

All *payers* and *policy makers* in the UK and about one third of the payers and one sixth of the policy makers in the Netherlands were recruited via an internet panel. Payers had to have paid employment in an occupation in which they were (in)directly involved in the financing or payment of integrated care and/or care for persons with multimorbidity. Policy makers had to be (in)directly involved in policy- or decision making about health care, social care or welfare. In the other countries, payers and policy makers were recruited through our own networks and snowball sampling. Examples of payer organisations that were approached include health care insurance companies and departments of municipalities responsible for paying social care. Examples of policy makers include politicians, public servants from the ministries of health, ministries of social care, provincial or local governments, and official governmental advisory bodies.

Patient and public involvement

The selection of the outcomes in the DCE was driven by the results of eight focus groups with in total 58 persons that had multiple chronic conditions. These focus groups were organised in 2016 in the countries participating in the SELFIE project.[15] As the participants of these focus groups frequently mentioned the importance of outcomes that went beyond the traditional health outcomes such as physical functioning, we included enjoyment of life, social relationships & participation, and resilience to represent aspects of wellbeing and 'positive health'.[16] They also frequently mentioned negative experiences related to the single-disease focus of each provider, misaligned treatment goals and advices, and fragmentation or duplication of care. That is the reason why 'person-centeredness' and 'continuity of care' were identified as the two key elements of experience of care.

The selection of outcomes was further informed by national workshops with stakeholder representatives in these countries, an international advisory board meeting within the SELFIE project (see acknowledgement), a review of outcomes measured in the 17 integrated care programmes, and a literature review on outcome measures of integrated care.[17] In each country, two to four persons with multimorbidity and two to four informal caregivers were present at the national workshops. One patient representative and one representative from an informal caregiver organisation were present at the international advisory board meeting.

The online DCE survey was pilot tested in six elderly with multimorbidity from the Netherlands, including two think-aloud completions of the online questionnaire. Their feedback was used to improve the visual design and instructions of the questionnaire.

The results of the weight elicitation study were discussed in national stakeholder workshops and the international advisory board meetings of the SELFIE project, where both patients and informal caregivers were present. They were reimbursed for their travel and accommodation costs and did not receive other remuneration.

Ethics approval

We adhered to the national regulations regarding medical ethics approvals and waivers. Letters of Medical Ethics Approval of the MCDA's and waivers were obtained from each country and forwarded to

For peer review only - http://bmjopen.bmj.com/site/about/guidelines.xhtml

the European Commission as a Deliverable of the SELFIE project. All respondents read the project information and provided online consent to take part before starting the online DCE survey.

Statistical analysis

A scale heterogeneity multinomial logit (S-MNL) model was used to analyse the responses by stakeholder group.[18,19] This model contains respondent-specific scale parameters that account for differential choice variability caused by the fact that in some respondents the error-terms are more important relative to the observed attribute-coefficients than in others. This is often interpreted as a difference in choice consistency.[20] The model also included a dummy variable to test whether respondents were more likely to choose the integrated care programme that was presented left or right. As this was not the case, this dummy was not included in the final model.

The relative importance weight of each of the included attributes was calculated as the contribution of the attribute's best level coefficient (level 3) to the sum of all attributes' best level coefficients. This was done because the weights, that will later be used in the MCDA's of the integrated care programmes,[6] need to represent the preference for the full swing from the worst to the best level of an attribute.

Differences in the mean preferences of stakeholder groups were evaluated using S-MNL models that analyzed all stakeholder groups within a country simultaneously. Multiplicative interaction terms were used to assess the difference in preferences between the first stakeholder group (i.e. patients with multimorbidity) and each of the other stakeholder groups. There is an indication of a difference when the 95% credible interval (CI) of the interaction term of a particular stakeholder group for a particular attribute does not contain 1.

All models were programmed in the BUGS language and estimated using OpenBUGS using Bayesian Markov Chain Monte Carlo (MCMC) methods (Syntax provided in online supplementary file Box S2).

Results

Respondents

Altogether, 5122 respondents completed the study, among which 1314 patients with multimorbidity. Of all patients who consented to complete the questionnaire, the proportion that completed all DCE questions was above 75%, except in Austria (67%). The mean time to complete the questionnaire was around 25 minutes (See online supplementary file Box S3).

Table 2 presents the characteristics of the respondents. Patients were older, less educated, in poorer health, and less often had a paid job than the other stakeholders. The health of the partners & informal caregivers was less good than the health of the professionals, payers and policy makers. There was a relatively high proportion of females among the professionals. The top five of most frequently reported diseases (not risk factors) among patients across all countries included 1) depression, anxiety or emotional difficulties, 2) chronic back pain or sciatica, 3) diabetes, 4) stomach problem, ulcer, gastritis or reflux, and 5) cardiovascular diseases (See online supplementary file Box S4).

For peer review only - http://bmjopen.bmj.com/site/about/guidelines.xhtml

(Table 2. Characteristics of the respondents included in the analysis)

Preferences of patients with multimorbidity

Table 3 shows the results of the regression analyses for the patients with multimorbidity. A positive regression coefficient means that respondents had a higher preference for (i.e., placed a higher weight on) that level of the outcome than level one. The larger the coefficient the stronger the preference. All outcomes contributed significantly to patients' choices, in the expected direction. The DCE-coefficients of outcome-levels two and three were almost all significantly different from level one. The coefficients of level three were always higher than of level two, except for non-significant disordered levels of social relationships & participation and person-centeredness in Germany, continuity of care in Norway and Spain, and costs in Spain.

The magnitude of the preference for the best level (level 3) was used as an indicator of the importance of the outcome measures. In all countries, except Hungary and Spain, enjoyment of life was valued most by patients, followed by either continuity of care (HR, DE), psychological wellbeing (NL, UK), physical functioning (NO), or resilience (AT). In Hungary, patients assigned the highest value to continuity of care, whereas in Spain, patients assigned the highest value to psychological wellbeing, both followed by enjoyment of life. In six of the eight countries (not HR and HU), physical functioning was placed in the top three. Social relationships & participation, person-centeredness and costs were generally valued lower by the patients than other outcomes. The scale parameter was highest in Austria, indicating that the difference in choice consistency was greatest among the Austrian patients.

(Table 3. Preferences for outcomes of integrated care of patients with multimorbidity)

Within-country comparison of preferences of patients and other stakeholders

Figure 2 presents the relative preference weights, sorted by importance among patients with multimorbidity. Within countries, there was considerable agreement between patients and the other stakeholders, with enjoyment of life mostly at the top and costs at the bottom. Nevertheless, there were differences.

As an indication of these differences, we counted the number of times that the 95% credibility intervals of the multiplicative interaction terms for stakeholder group and attribute excluded one (See online supplementary file Box S5). Of all stakeholder groups, the preferences of professionals differed most often from the preferences of patients. These differences were most frequently related to physical functioning and person-centeredness. Physical functioning was valued lower by professionals than patients in Austria, Germany, Netherlands, Norway and Spain. Person-centeredness was valued higher by professionals than patients in Austria, Hungary, Germany, and Spain.

Differences in relative weights between patients and payers/policy makers within a country were most frequently related to costs, which was more important to the latter in Austria, Germany, the Netherlands, and Norway. However, the relative weight that they assigned to costs remained low.

For peer review only - http://bmjopen.bmj.com/site/about/guidelines.xhtml

Payers and/or policy makers assigned lower values to continuity of care (HU, NL, NO), and resilience (DE, ES, HU, NL) than patients.

When the partners & other informal caregivers' weights differed from the patients, that mostly related to physical functioning, which was valued lower than patients in Austria, Germany, Netherlands, and Norway and higher than patients in Spain.

(Figure 2. Relative weight of the outcomes by stakeholder and country.)

Discussion

Main findings

This is the first international preference study among such a large number of persons with multimorbidity and other stakeholders involved in decision making about person-centered integrated care. We found that there was considerable agreement between patients, partners & other informal caregivers, professionals, payers and policy makers. In all countries, they assigned the highest or second highest value to enjoyment of life and the lowest or second lowest value to costs. Of the health and wellbeing outcomes, psychological wellbeing, physical functioning and resilience were generally valued higher than social relationships & participation. Of the two experience outcomes, continuity of care was generally valued higher than person-centeredness. Patients as well as the other stakeholders in the two Eastern European countries, Hungary and Croatia, assigned a relatively high value to continuity of care. Continuity of care also entered the top-three of professionals, payers and policy makers in Germany and Austria. Representatives from Eastern European countries suggested that the high preference assigned to continuity of care might illustrate problems with access to care and long waiting lists in various parts of their healthcare systems. German and Austrian stakeholders also pointed to a relation with capacity issues in especially the elderly care in their countries.

We also found indications of within-country differences in preferences between patients and other stakeholders. Of all stakeholder groups, professionals' preferences differed most frequently from patients' preferences and these differences most often pertained to physical functioning and person-centeredness. Professionals assigned lower values to physical functioning than patients in five countries, and higher values to person-centeredness in four countries. Preference heterogeneity was also found for costs, which received higher values from payers and policy makers than from patients in four countries. However, the relative value of costs compared to the other outcomes remained low for these stakeholders as well. When discussing why costs commonly ranked last, even among payers and policy makers, stakeholder representatives generally felt that this was in not in line with real world decision making. The low value that patients assigned to costs might be related to the fact that they were informed that these costs did not have to be paid out-of-pocket. The low value assigned by payers and policy makers might be a reflection of the normative viewpoint that costs should not play an important role when it comes to health. It might also be related to the challenging task of separating their professional role from the other roles they might have. Payers and policy makers can easily relate to, or might be, patients and informal caregivers themselves.

Comparison with previous research

For peer review only - http://bmjopen.bmj.com/site/about/guidelines.xhtml

In contrast to most previous DCE studies of similar interventions, we defined the DCE attributes in terms of Triple Aim outcomes and not in terms of characteristics of the services provided. The choices made by the respondents indicated that they did make trade-offs between health, wellbeing and experience outcomes. That finding is in line with a recent study that also measured preferences for both health and wellbeing outcomes in the same valuation procedure. They did so in response to the recognition that the Quality Adjusted Life Year (QALY) does not capture the full value of interventions that involve social care.[21] Mulhern et al. included the health domains of the EQ-5D (EuroQol five-dimension questionnaire) and the wellbeing domains of the ASCOT (Adult Social Care Outcomes Toolkit) in the same DCE and found that the top-4 of most valued outcomes by the Australian general public included one ASCOT domain, namely 'control over ones daily life', which ranked fourth after the EQ-5D domains pain and discomfort, mobility and anxiety/depression.[21] They further found that social participation got the lowest value. This is in line with our study, where this outcome ended-up in the bottom-three in five countries (AT, DE, HR, HU, NL), based on patients' values.

Mühlbacher and Bethge conducted a series of online DCE's among US patients and German insurees (not only patients) that focused on different features and building blocks of integrated service delivery.[22] In contrast to our study, costs were an important attribute in both countries. It ranked highest in Germany and in the US only 'shared decision making' was slightly more important. The difference is most likely explained by the fact that costs were defined as out-of-pocket costs.

Burton et al, conducted a DCE that specifically aimed to value different aspects of person-centeredness in UK patients with either chronic pain or chronic respiratory disease. They found that 'taking account of the current living situation', and 'working with the patients on what they want to get from life' were the most important aspects.[23] Although our definition of person-centeredness as 'care that matches an individual's needs, capabilities, and preferences and where decisions are made jointly based on good information' more or less incorporated these two aspects, that did not prevent this outcome to be ranked relatively low when it was compared to the other outcomes by the patients in Western European countries.

Limitations of the study

The first limitation is the cognitive challenge of completing a DCE. Respondents are expected to understand and interpret the definitions of the outcomes as provided. For that reason, we did not only explain the outcomes before the start of the DCE task, but also ensured that the definitions of the outcomes became visible in every choice task when respondents hovered the mouse over an outcome-heading. Respondents are further expected to consider all outcomes and make choices based on trade-offs between all outcomes. It has been reported in the literature that, although the majority of respondents does this, lower educated and less health literate respondents more often use simplified heuristics such as basing their choices on one or two high priority attributes.[24] To reduce the complexity of the choices, we have used attribute-level overlap and colour coding. Nevertheless, the proportion of patients that found the questionnaire (very) difficult varied from 8.4% in Hungary to 35.3% in Norway. However, the observation that only a small proportion of patients in all countries completed the questionnaire in less than five minutes may illustrate their engagement in the DCE task. Excluding these patients from the analyses did not affect the results (available upon request).

For peer review only - http://bmjopen.bmj.com/site/about/guidelines.xhtml

The second limitation is related to the assumption of preference independence underlying an MCDA,[8] which means that the preference for a particular outcome is not influenced by the level of performance on the other outcomes. This was an important criterion when selecting the outcomes for the DCE. It was also the reason why enjoyment of life was framed positively (having pleasure and happiness in life) and psychological wellbeing was framed negatively (the absence of stress, worrying, listlessness, anxiety, and feeling down). However, that does not avoid conceptual overlap entirely. We acknowledge that enjoyment of life is a broad outcome measure that might be associated with some of the other outomes measures.

A last limitation concerns the generalizability of the results, especially of the persons with multimorbidity and the informal caregivers which were recruited through an existing internet panel. Because of that, respondents with lower levels of education, health literacy and health numeracy are likely to be underrepresented in our study. This does not apply to Spain, where patients were recruited by professionals, which might have led to an over-representation of patients with high healthcare utilization and informal caregivers with a higher burden of informal care.

Implications

First, the high preference for enjoyment of life, which was defined as having pleasure and happiness in life, underlines the importance of measuring outcomes beyond health to assess the wider value of integrated care programmes for people with multimorbidity. This is not often done, and certainly not routinely. Second, this high preference for enjoyment of life calls for more attention for social and mental support, something which is not exclusively provided by the healthcare sector. Patients care more about the big picture, i.e., the pleasure and happiness in life, rather than the process of care, unless there is something clearly lacking in this process, such as continuity of care. Third, although it is good that many of the current integrated care programmes already pay a lot of attention to wellbeing and person-centeredness, these efforts should not divert attention from improving physical functioning where possible. Fourth, the differences in relative preferences for physical functioning and person-centeredness between patients and professionals in several countries stress the need for shared decision making to prioritise services that improve outcomes most important to patients. Fifth, the existence of preference heterogeneity with respect to outcomes of integrated care among stakeholders stresses the importance of informing decision makers from multiple perspectives. The MCDA's of the 17 integrated care programmes that we are conducting are particularly suited for that.[7] These MCDA's will be used to inform pragmatic, local and regional level decisions on different options to implement, adapt, or scale-up existing integrated care programmes. In this context, the downside of an MCDA of using an outcome that does not allow a comparison between integrated care and other interventions, like the QALY, is acceptable. When such a comparison is required for system-level reimbursement decisions, it can be informative to compare the results of the MCDA to the results of a cost per QALY analysis.

Conclusion

Integrated care programmes aim to improve multiple outcomes. The most important outcome to patients with multimorbidity was the wellbeing outcome enjoyment of life, which calls for a greater involvement of the social and mental care sector. This also stresses the importance of including

For peer review only - http://bmjopen.bmj.com/site/about/guidelines.xhtml

outcomes beyond health into the evaluation of these programmes. Process-related outcomes were less important to patients unless there were issues with the process, like the lack of continuity of care. The other stakeholders largely agreed with the preferences of the patients, although the preference heterogeneity with respect to physical functioning and person-centeredness underlines the importance of shared decision making between patients with multimorbidity and professionals.

For peer review only

Acknowledgements

We would like to thank all the anonymous respondents who devoted time to the completion of the weight-elicitation study. We are grateful for the contributions of the members of the international advisory board of the SELFIE project and the participants of the national workshops. The advisory board consisted of Rudi Westendorp (Center for Healthy Ageing), Ronald van Breugel (health insurer VGZ), Jan de Maeseneer (Emeritus professor of Family Medicine), Mieke Rijken (NIVEL), Viktoria Stein (International Foundation of Integrated Care), Luke Slawomirski (OECD), Ian Forde (formerly OECD), Rick Greene (International Alliance of Carer Organizations), Nadine Henningsen (Canadian Homecare Organization), Dominik Tomek, (European Patients' Forum), Derek Steward (NHS NIHR, Patient and Public Health Involvement), Antonia Croy (patient advocate), and Ikka Kunnamo (Editor in Chief Evidence-Based Medicine Guidelines, Duodecim Medical Publications). We are also grateful for the input that we have received from the participants of the national workshops in the eight participating countries.

For peer review only

Funding statement

The SELFIE project has received funding from the European Union's Horizon 2020 research and innovation program under grant agreement No 634288.

Disclaimer

The content of this paper reflects only the SELFIE group's views and the European Commission is not liable for any use that may be made of the information contained herein.

Conflict of interest statement

All authors have completed the ICMJE uniform disclosure form at www.icmje.org/coi_disclosure.pdf and declare: all authors had financial support from the European Union's Horizon 2020 research and innovation program under grant agreement No 634288 for the submitted work; no financial relationships with any organisations that might have an interest in the submitted work; no other relationships or activities that could appear to have influenced the submitted work.

Data sharing statement

The individual-level DCE data will not be made publicly available. Upon request to the corresponding author, we may consider in collaboration with the respective SELFIE-partner, whether country-specific data can be shared. Other findings from the overarching SELFIE study can be found on the SELFIE website https://www.selfie2020.eu/.

For peer review only

Contributorship statements

MRvM was the coordinator of SELFIE, designed the study, had access to the data from all countries, supervised the analyses, controlled the decision to publish, and wrote the paper. She accepts full responsibility for the conduct of the study.

MK was part of the coordinating team of SELFIE, contributed to data analysis, interpretation of data for all countries, and writing the paper.

FL was part of the coordinating team of SELFIE, co-designed the study, contributed to data collection, data analyses, interpretation of data for all countries, and reviewed the paper.

MH was part of the coordinating team of SELFIE, co-designed the study, contributed to data collection, data analyses, interpretation of data for all countries, and reviewed the paper.

WL was part of the coordinating team of SELFIE, co-designed the study, contributed to data collection, data analyses, interpretation of data for all countries, and reviewed the paper

KI contributed to the translation of the DCE into Norwegian, data collection and interpretation in Norway, and reviewed the paper.

JEA contributed to the translation of the DCE for Norway, data collection and interpretation in Norway, and reviewed the paper.

MKr contributed to the translation of the DCE for Austria, data collection and interpretation in Austria, and reviewed the paper.

VS contributed to the translation of the DCE for Germany, data collection and interpretation in Germany, and reviewed the paper.

DR contributed to the translation of the DCE for Croatia, data collection and interpretation in Croatia, and reviewed the paper.

JGP contributed to the translation of the DCE for Hungary, data collection and interpretation in Hungary, and reviewed the paper.

IC contributed to the translation of the DCE for Spain, data collection and interpretation in Spain, and reviewed the paper.

JS contributed to the translation of the DCE for the UK, data collection and interpretation in the UK, and reviewed the paper.

MJ optimised the DCE designs, conducted the Bayesian statistical analyses, and reviewed the paper.

For peer review only

For peer review only - http://bmjopen.bmj.com/site/about/guidelines.xhtml

Table 1. Definitions of the outcomes and levels.

Outcome (attribute)	Definition	Levels
Physical functioning	acceptable physical functioning and being able to do daily activities without needing assistance (e.g., getting dressed, sitting down and getting up from a chair, taking your medications)	1. Severely limited in physical functioning and activities of daily living<br>2. Moderately limited in physical functioning and activities of daily living<br>3. Hardly or not at all limited in physical functioning and activities of daily living
Psychological wellbeing	the absence of stress, worrying, listlessness, anxiety, and feeling down	1. Always or mostly stressed, worried, listless, anxious, and down<br>2. Regularly stressed, worried, listless, anxious, and down<br>3. Seldom or never stressed, worried, listless, anxious, and down
Social relationships & participation	having meaningful connections with others as desired	1. No or barely any meaningful connections with others<br>2. Some meaningful connections with others<br>3. A lot of meaningful connections with others
Enjoyment of life	having pleasure and happiness in life	1. No or barely any pleasure and happiness in life<br>2. Some pleasure and happiness in life<br>3. A lot of pleasure and happiness in life
Resilience	the ability to recover from or adjust to difficulties and to restore one's balance	1. Poor ability to recover, adjust, and restore balance<br>2. Fair ability to recover, adjust, and restore balance<br>3. Good ability to recover, adjust, and restore balance
Person-centeredness	care that matches an individual's needs, capabilities, and preferences and where decisions are made jointly based on good information	1. Not or barely person-centered<br>2. Somewhat person-centered<br>3. Highly person-centered
Continuity of care	good collaboration, smooth transitions between caregivers, and no waste of time	1. Poor collaboration, transitions, and timeliness<br>2. Fair collaboration, transitions, and timeliness<br>3. Good collaboration, transitions, and timeliness
Total health- and social care costs	total health- and social care costs per participant in the programme, per year. Note:	1. 8500 euro per participant per year *<br>2. 7000 euro per participant per year<br>3. 5500 euro per participant per year

For peer review only - http://bmjopen.bmj.com/site/about/guidelines.xhtml

	These are costs paid for by the health insurer / government.	

* These are the Dutch values. The value for the other countries are: Austria: 8,000, 6,600, 5,200 euro; Croatia: 7,200, 6,000, 4,800 kuna (973, 810, 648 euro); Hungary: 600,000, 500,000, 400,000 forint (1,951, 1,626, 1,300 euro); Germany: 4,800, 4,000, 3,200 euro; Norway: 115,000, 95,000, 75,000 krone (12,330, 10,185, 8,041 euro); Spain: 5,400, 4,500, 3,600 euro; United Kingdom: 3,600, 3,000, 2,400 pounds (4,130, 3,441, 2,753 euro)

For peer review only

Table 2. Characteristics of the respondents included in the analysis.

	Patients	Partners	Professionals	Payers	Policy makers
**Austria**	N=169	N=184	N=144	N=99 (combined)	
Age mean (SD)	51.0 (15.0)	42.9 (14.7)	44.7 (8.8)	45.8 (10.7)	
Gender (% female)	78 (46.2)	103 (56.0)	110 (77.5)	53 (53.5)	
Educational level (%)^					
Low	15 (8.9)	10 (5.4)	0 (0)	0 (0)	
Medium	126 (74.5)	125 (67.9)	40 (28.1)	15 (15.2)	
High	28 (16.6)	49 (26.6)	102 (71.8)	81 (84.9)	
General health (%)					
Poor or Fair	87 (51.5)	30 (16.3)	4 (2.8)	2 (2)	
Good	67 (39.6)	70 (38.0)	33 (23.2)	23 (23.2)	
Very good or excellent	15 (8.9)	70 (45.7)	102 (71.8)	24 (74.7)	
Number of health problems (mean, SD)	3.4 (2.2)	2.0 (2.0)	0.7 (1.1)	0.6 (1.0)	
**Croatia**	N=168	N=169	N=103	N=73 (combined)	
Age	43.1 (13.7)	42.6 (10.8)	44.4 (11.5)	45.3 (10.3)	
Gender (% female)	104 (61.9)	96 (56.8)	79 (76.7)	62 (84.9)	
Educational level (%)^					
Low	87 (51.8)	91 (53.9)	7 (6.8)	5 (6.8)	
Medium	39 (23.2)	33 (19.5)	38 (36.9)	26 (35.6)	
High	42 (25.0)	45 (26.7)	58 (56.4)	42 (57.5)	
General health (%)					
Poor or Fair	74 (44.1)	25 (14.8)	9 (8.8)	8 (10.9)	
Good	44 (26.2)	27 (16.0)	26 (25.2)	21 (28.8)	
Very good or excellent	50 (29.8)	117 (69.3)	68 (66.0)	44 (60.2)	
Number of health problems (mean, SD)	3.2 (2.3)	1.6 (1.6)	1.2 (1,3)	1.25 (1.6)	
**Germany**	N=160	N=208	N=170	N=110 (combined)	
Age	52.7 (14.6)	46.8 (14.5)	42.4 (10.7)	45.6 (11.0)	
Gender (% female)	77 (48.1)	102 (49.0)	123 (73.7)	65 (59.1)	
Educational level (%)^					
Low	53 (33.2)	76 (36.5)	31 (18.6)	2 (1.8)	
Medium	85 (53.2)	90 (43.2)	68 (40.7)	36 (32.7)	
High	22 (13.9)	42 (20.2)	68 (40.7)	72 (65.4)	
General health (%)					
Poor or Fair	106 (66.3)	49 (23.5)	18 (10.8)	8 (7.3)	
Good	48 (30.0)	98)47.1)	66 (39.5)	36 (32.7)	
Very good or excellent	6 (3.8)	61 (29.4)	83 (49.7)	66 (60.0)	
Number of health problems (mean, SD)	4.7 (2.5)	2.6 (2.7)	1.4 (1.7)	0.87 (1.2)	
**Hungary**	N=192	N=166	N=163	N=153 (combined)	
Age	51.1 (14.1)	43.9 (14.3)	45.4 (10.9)	46.7 (10.8)	
Gender (% female)	102 (53.1)	80 (48.2)	84 (51.5)	101 (66.0)	
Educational level (%)^					

For peer review only

For peer review only - http://bmjopen.bmj.com/site/about/guidelines.xhtml

	Patients	Partners	Professionals	Payers	Policy makers
Low	65 (33.9)	51 (30.7)	1 (0.6)	3 (2.0)	
Medium	70 (36.5)	80 (48.2)	13 (8.0)	21 (13.7)	
High	57 (29.7)	35 (21.1)	149 (90.4)	129 (84.3)	
General health (%)					
Poor or Fair	120 (62.5)	44 (26.5)	23 (14.1)	18 (11.8)	
Good	61 (31.8)	71 (42.8)	46 (28.2)	50 (32.7)	
Very good or excellent	11 (5.7)	51 (30.7)	94 (57.7)	85 (55.6)	
Number of health problems (mean, SD)	4.0 (2.4)	2.1 (2.1)	1.1 (1.5)	1.1 (1.4)	
**Netherlands**	N=156	N=158	N=155	N=104	N=151
Age	60.4 (11.8)	52.2 (13.0)	42.0 (12.5)	42.8 (11.7)	46.3 (12.1)
Gender (% female)	77 (49.0)	81 (51.3)	86 (55.5)	48 (46.2)	95 (62.9)
Educational level (%)^					
Low	14 (9.0)	8 (5.1)	1 (0.6)	0	2 (1.3)
Medium	70 (45.0)	76 (48.1)	48 (31.0)	1 (1.0)	0
High	72 (46.2)	74 (46.8)	106 (68.3)	103 (99.1)	149 (89.7)
General health (%)					
Poor or Fair	86 (55.2)	40 (25.3)	11 (7.1)	7 (6.7)	14 (9.2)
Good	63 (40.4)	77 (48.7)	59 (38.1)	38 (36.5)	51 (33.8)
Very good or excellent	7 (4.5)	42 (26.0)	85 (54.9)	59 (56.8)	86 (57.0)
Number of health problems (mean, SD)	4.0 (1.9)	2.2 (2.5)	1.1 (1.5)	0.7 (1.0)	0.8 (1.2)
**Norway**	N=156	N=156	N=162	N=122	N=180
Age	56.5 (14.7)	53.7 (12.9)	45.7 (10.7)	51.3 (10.3)	54.7 (13.2)
Gender (% female)	70 (44.9)	78 (50.00)	124 (76.5)	68 (55.7)	97 (53.9)
Educational level (%)^					
Low	11 (7.1)	1 (0.6)	2 (1.2)	1 (0.8)	0
Medium	52 (33.3)	39 (25.0)	4 (2.5)	1 (0.8)	35 (19.4)
High	93 (59.6)	116 (74.4)	156 (96.3)	120 (98.4)	145 (80.6)
Poor or Fair	78 (50.0)	38 (24.4)	8 (4.9)	5 (4.1)	17 (9.4)
Good	47 (30.1)	64 (41.0)	33 (20.4)	17 (13.9)	50 (27.8)
Very good or excellent	31 (19.9)	54 (34.6)	121 (74.7)	100 (82)	113 (62.8)
Number of health problems (mean, SD)	3.6 (1.7)	2.2 (1.7)	1.1 (1.5)	0.79 (1.1)	1.5 (1.6)
**Spain**	N=150	N=151	N=152		
Age	62.8 (9.8)	55.3 (11.7)	40.8 (10.7)		
Gender (% female)	65 (43.3)	103 (68.2)	40.76 (10.65)		
Educational level (%)^					
Low	68 (45.4)	57 (37.8)	0		
Medium	39 (26)	54 (35.7)	30 (19.7)		
High	43 (28.7)	40 (26.4)	122 (80.3)		
General health (%)					
Poor or Fair	69 (46.0)	45 (29.8)	2 (1.3)		
Good	63 (42.0)	70 (46.4)	30 (19.7)		

For peer review only - http://bmjopen.bmj.com/site/about/guidelines.xhtml

	Patients	Partners	Professionals	Payers	Policy makers
Very good or excellent	18 (12.0)	36 (23.8)	120 (78.9)		
Number of health problems (mean, SD)	3.9 (2.5)	2.08 (1.7)	0.9 (1.0)		
**United Kingdom**	N=163	N=233	N=161	N=181 (combined)	
Age	56.4 (14.4)	45 (13.8)	47.7 (10.8)	45.5 (11.0)	
Gender (% female)	94 (57.7)	126 (54.1)	51 (31.7)	74 (40.9)	
Educational level (%)^					
Low	60 (36.8)	60 (25.8)	0	3 (1.7)	
Medium	45 (27.6)	57 (24.5)	2 (1.2)	13 (7.2)	
High	58 (35.6)	116 (49.7)	159 (98.8)	165 (91.1)	
General health (%)					
Poor or Fair	129 (79.2)	61 (26.2)	5 (3.1)	18 (10)	
Good	28 (17.2)	75 (32.2)	15 (9.3)	33 (18.2)	
Very good or excellent	6 (3.7)	97 (41.7)	141 (87.6)	130 (71.8)	
Number of health problems (mean, SD)	4.6 (2.4)	1.9 (2.1)	0.7 (1.0)	1.1 (1.6)	

^low: no post-secondary education; high: Bachelor's degree or higher

For peer review only

Table 3. Preferences for outcomes of integrated care of patients with multimorbidity.

		Coefficients Bayesian S-MNL model: mean and 95% credibility interval															
Outcome	Level	AT		HR		HU		DE		NL		NO		ES		UK	
Physical functioning	2	1.11	0.73-1.78	0.59	0.40-0.77	0.47	0.30-0.63	0.81	0.61-1.02	1.25	1.00-1.50	1.74	1.46-2.02	1.41	1.14-1.71	0.92	0.72-1.13
	3	1.19	0.79-1.87	0.71	0.51-0.91	0.69	0.50-0.87	1.00	0.77-1.24	1.95	1.65-2.28	1.93	1.61-2.26	1.44	1.14-1.78	1.22	0.99-1.46
Psycholo-gical wellbeing	2	0.43	0.22-0.71	0.65	0.47-0.82	0.005	-0.14-0.16	0.25	0.07-0.43	0.94	0.73-1.17	0.94	0.72-1.16	1.26	0.97-1.59	0.55	0.37-0.73
	3	0.98	0.63-1.57	1.05	0.83-1.28	0.33	0.16-0.50	0.64	0.41-0.88	2.03	1.72-2.38	1.89	1.60-2.19	1.69	1.37-2.03	1.30	1.08-1.54
Social relations & participation	2	0.53	0.34-0.78	0.67	0.50-0.84	0.37	0.22-0.52	0.52	0.34-0.70	0.85	0.66-1.06	1.08	0.85-1.30	1.17	0.90-1.45	0.78	0.60-0.97
	3	0.62	0.39-0.97	0.81	0.61-1.01	0.58	0.41-0.75	0.48	0.30-0.68	1.03	0.81-1.27	1.20	0.97-1.44	1.24	0.98-1.54	1.02	0.83-1.22
Enjoyment of life	2	1.14	0.81-1.71	0.95	0.77-1.14	0.59	0.42-0.75	0.98	0.79-1.19	1.80	1.54-2.08	1.94	1.66-2.24	1.63	1.31-1.98	1.50	1.28-1.73
	3	1.56	1.15-2.28	1.41	1.18-1.65	0.72	0.54-0.91	1.36	1.12-1.62	2.76	2.45-3.14	2.68	2.31-3.08	1.65	1.30-2.05	2.16	1.88-2.47
Resilience	2	0.89	0.58-1.44	0.72	0.55-0.91	0.60	0.44-0.76	0.72	0.54-0.92	1.37	1.15-1.59	0.85	0.65-1.04	1.06	0.82-1.31	0.79	0.60-0.98
	3	1.19	0.85-1.80	1.13	0.93-1.34	0.71	0.54-0.89	1.04	0.83-1.27	1.81	1.56-2.08	1.19	0.97-1.42	1.18	0.92-1.47	1.09	0.88-1.30
Person-centered-ness	2	0.05	-0.17-0.25	0.68	0.51-0.86	0.46	0.31-0.62	0.31	0.14-0.48	0.52	0.32-0.71	0.31	0.12-0.50	0.63	0.43-0.84	0.47	0.31-0.64
	3	0.31	0.11-0.53	0.96	0.78-1.15	0.71	0.53-0.90	0.26	0.08-0.44	0.95	0.74-1.16	0.50	0.31-0.70	0.72	0.50-0.95	0.72	0.53-0.90
Continuity of care	2	0.66	0.43-1.00	0.91	0.73-1.10	0.86	0.70-1.03	0.66	0.48-0.84	1.08	0.85-1.31	1.30	1.07-1.54	0.70	0.49-0.93	0.72	0.53-0.91
	3	0.97	0.70-1.38	1.19	0.99-1.40	1.00	0.82-1.18	1.08	0.85-1.32	1.28	1.05-1.53	1.20	0.97-1.44	0.63	0.42-0.85	0.94	0.74-1.15
Total costs	2	0.38	0.15-0.69	0.21	0.06-0.37	0.15	-0.002-0.30	0.18	0.02-0.34	0.17	-0.01-0.34	0.07	-0.12-0.25	0.35	0.15-0.56	0.36	0.18-0.54

For peer review only

For peer review only - http://bmjopen.bmj.com/site/about/guidelines.xhtml

3	0.44	0.21-0.76	0.24	0.07-0.42	0.19	0.03-0.36	0.27	0.10-0.44	0.43	0.24-0.62	0.23	0.05-0.42	0.15	-0.06-0.38	0.58	0.40-0.77
Scale parameter tau	1.39	0.21-1.12	0.57	0.38-0.77	0.72	0.47-1.02	1.07	0.75-1.60	0.65	0.48-0.84	0.58	0.41-0.78	1.23	0.83-2.05	0.56	0.38-0.74
No. of observations	6,120		6,228		6,984		5,940		5,760		5,688		5,400		5,940	
No. of respondents	170		173		194		166		160		158		150		165	

For peer review only

Figure 1. Example of a DCE question.

Figure 2. Relative preferences for outcomes of all stakeholders, sorted by the preferences of patients with multi-morbidity in each country (order of bars at each outcome: patients, partners and other informal caregivers, professionals, payers and policy makers);

Footnote Figure 2 * 95% credibility interval excludes 1, indicating that the stakeholder group differed from the patients with multi-morbidity (See online supplementary file box S5). The relative weight of the partners and other informal caregivers is 0 in Hungary. The Netherlands and Norway have 5 bars per attribute because they had respondents from all five stakeholder groups. The other countries combined the respondents from the payers and policy makers into one group and therefore have 4 bars per attribute, except for Spain which has 3 bars because they had not administered the questionnaire to payers and policy makers.

For peer review only

References

1. Banerjee S. Multimorbidity--older adults need health care that can count past one. *Lancet* 2015;385(9968):587-89.
2. Rijken M, Hujala A, van Ginneken E, et al. Managing multimorbidity: Profiles of integrated care approaches targeting people with multiple chronic conditions in Europe. *Health policy* 2018;122(1):44-52.
3. Struckmann V, Leijten FRM, van Ginneken E, et al. Results of a scoping review on integrated care for persons with multi-morbidity. *Health policy* 2018;122(1):23-35.
4. Leijten FRM, Struckmann V, van Ginneken E, et al. The SELFIE framework for integrated care for multi-morbidity: Development and description. *Health policy* 2018;122(1):12-22.
5. Berwick DM, Nolan TW, Whittington J. The triple aim: care, health, and cost. *Health affairs* 2008;27(3):759-69.
6. Rutten-van Mölken MPMH, Leijten F, Hoedemakers M, et al. Strengthening the evidence-base of integrated care for people with multi-morbidity in Europe using Multi-Criteria Decision Analysis (MCDA). *BMC health services research* 2018;18:576.
7. Thokala P, Duenas A. Multiple criteria decision analysis for health technology assessment. *Value in health : the journal of the International Society for Pharmacoeconomics and Outcomes Research* 2012;15(8):1172-81.
8. Marsh K, M IJ, Thokala P, et al. Multiple Criteria Decision Analysis for Health Care Decision Making--Emerging Good Practices: Report 2 of the ISPOR MCDA Emerging Good Practices Task Force. *Value in health : the journal of the International Society for Pharmacoeconomics and Outcomes Research* 2016;19(2):125-37.
9. Hensher D, Rose J, Greene W. Applied Choice Analysis: A Primer.: Cambridge: Cambridge University Press 2005.
10. Louviere J, Hensher D, J S. Stated Choice Methods: Analysis and Application.: Cambridge: Cambridge University Press 2000.
11. Ryan M, Farrar S. Using conjoint analysis to elicit preferences for health care. *Britisch Medical Journal* 2000;320(7248):1530-3.
12. Sandor Z, Wedel M. Heterogeneous Conjoint Choice Designs. *J Marketing Research* 2005;42(2):210-18.
13. Jonker MF, Donkers B, de Bekker-Grob E, et al. Attribute level overlap (and color coding) can reduce task complexity, improve choice consistency, and decrease the dropout rate in discrete choice experiments. *Health economics* 2019;28(3):350-63.
14. de Bekker-Grob EW, Donkers B, Jonker MF, et al. Sample Size Requirements for Discrete-Choice Experiments in Healthcare: a Practical Guide. *The patient* 2015;8(5):373-84.
15. Leijten FRM, Hoedemakers M, Struckmann V, et al. Defining good health and care from the perspective of persons with multimorbidity: results from a qualitative study of focus groups in eight European countries. *BMJ open* 2018;8(8):e021072.
16. Huber M, Knottnerus JA, Green L, et al. How should we define health? *Bmj* 2011;343:d4163.
17. Boland M, Leijten F, Hoedemakers M, et al. Outcomes and indicators in integrated care for persons with multi-morbidity. 2017. https://www.selfie2020.eu/wp-content/uploads/2018/05/SELFIE-WP4-Deliverable-Report-1.pdf.
18. Fiebig DG, Keane MP, Louviere J, et al. The generalized multinomial logit model: accounting for scale and coefficient heterogeneity. *Marketing Science* 2010;29(3):393-421.

For peer review only - http://bmjopen.bmj.com/site/about/guidelines.xhtml

19. Greene WH, Hensher DA. Does scale heterogeneity across individuals matter? An empirical assessment of alternative logit models. *Transportation* 2010;37(3):413-28.
20. Lancsar E, Fiebig DG, Hole AR. Discrete Choice Experiments: A Guide to Model Specification, Estimation and Software. *PharmacoEconomics* 2017;35(7):697-716.
21. Mulhern B, Norman R, De Abreu Lourenco R, et al. Investigating the relative value of health and social care related quality of life using a discrete choice experiment. *Social science & medicine* 2019;233:28-37.
22. Muhlbacher A, Bethge S. Patients Preferences. In: Ameling V SU, Goodwin N, Balicer R, Nolte E, Suter E,, ed. Handbook Integrated Care. 1st ed. Cham Switzerland: Springer 2017.
23. Burton CD, Entwistle VA, Elliott AM, et al. The value of different aspects of person-centred care: a series of discrete choice experiments in people with long-term conditions. *BMJ open* 2017;7(4):e015689.
24. Veldwijk J, Determann D, Lambooij MS, et al. Exploring how individuals complete the choice tasks in a discrete choice experiment: an interview study. *BMC medical research methodology* 2016;16:45.

For peer review only

For peer review only - http://bmjopen.bmj.com/site/about/guidelines.xhtml

	Care programme A	Care programme B
ⓘ **Physical functioning**	**Severely** limited in physical functioning and activities of daily living	**Moderately** limited in physical functioning and activities of daily living
ⓘ **Psychological well-being**	**Always or mostly** stressed, worried, listless, anxious, and down	**Always or mostly** stressed, worried, listless, anxious, and down
ⓘ **Social relationships and participation**	**No or barely any** meaningful connections with others	**No or barely any** meaningful connections with others
ⓘ **Enjoyment of life**	**Some** pleasure and happiness in life	**No or barely any** pleasure and happiness in life
ⓘ **Resilience**	**Good** ability to recover, adjust, and restore balance	**Good** ability to recover, adjust, and restore balance
ⓘ **Person-centeredness**	**Highly** person-centred	**Highly** person-centred
ⓘ **Continuity of care**	**Poor** collaboration, transitions, and timeliness	**Fair** collaboration, transitions, and timeliness
ⓘ **Total health- and social care costs**	**5600 pounds** per participant per year	**7000 pounds** per participant per year
**Which care programme do you prefer, A or B?**	**A** ◯	**B** ◯

only

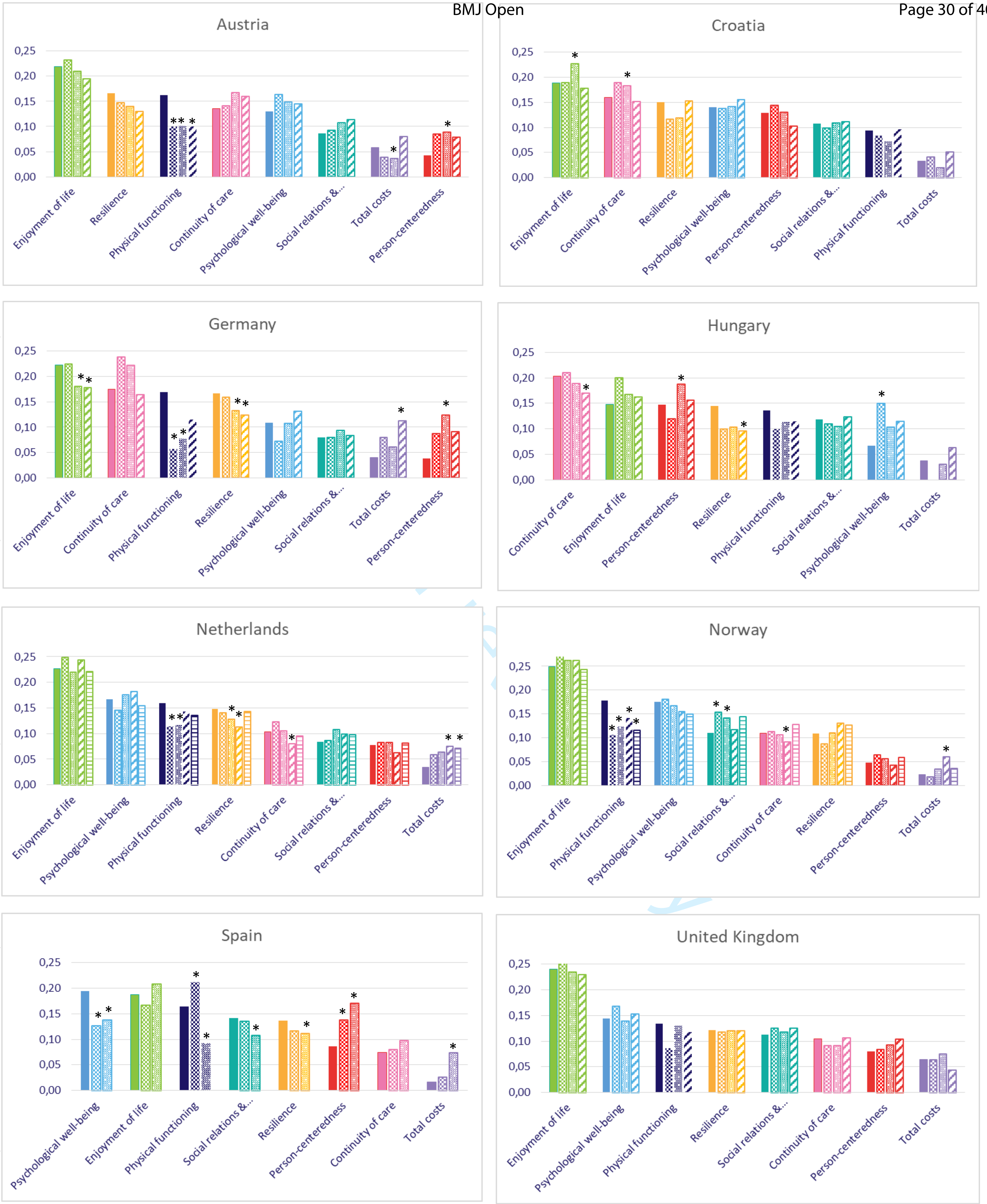
Austria
0,25
0,20
0,15
0,10
0,05
0,00
Enjoyment of life
Resilience
Physical functioning
Continuity of care
Psychological well-being
Social relations &...
Total costs
Person-centeredness
Croatia
Enjoyment of life
Continuity of care
Resilience
Psychological well-being
Person-centeredness
Social relations &...
Physical functioning
Total costs
Germany
Enjoyment of life
Continuity of care
Physical functioning
Resilience
Psychological well-being
Social relations &...
Total costs
Person-centeredness
Hungary
Continuity of care
Enjoyment of life
Person-centeredness
Resilience
Physical functioning
Social relations &...
Psychological well-being
Total costs
Netherlands
Enjoyment of life
Psychological well-being
Physical functioning
Resilience
Continuity of care
Social relations &...
Person-centeredness
Total costs
Norway
Enjoyment of life
Physical functioning
Psychological well-being
Social relations &...
Continuity of care
Resilience
Person-centeredness
Total costs
Spain
Psychological well-being
Enjoyment of life
Physical functioning
Social relations &...
Resilience
Person-centeredness
Continuity of care
Total costs
United Kingdom
Enjoyment of life
Psychological well-being
Physical functioning
Resilience
Social relations &...
Continuity of care
Person-centeredness
Total costs

Online supplementary file

Box S1. Original, literature-based, priors.

	DCE-Level		
**DCE-Attribute**	**Worst (1)**	**Average (2) Mean (SD)**	**Best (3) Mean (SD)**
Physical functioning	Reference	0.30 (0.12)	0.60 (0.23)
Psychological wellbeing	Reference	0.20 (0.08)	0.40 (0.16)
Social relations & participation	Reference	0.25 (0.10)	0.45 (0.18)
Enjoyment of life	Reference	0.20 (0.08)	0.40 (0.16)
Resilience	Reference	0.20 (0.08)	0.40 (0.16)
Person-centeredness	Reference	0.10 (0.04)	0.25 (0.10)
Continuity of care	Reference	0.15 (0.06)	0.30 (0.12)
Total costs	Reference	0.15 (0.06)	0.35 (0.14)

For peer review only

For peer review only - http://bmjopen.bmj.com/site/about/guidelines.xhtml

Box S2. Example of OpenBUGS model code for the S-MNL models.

All models were programmed in the BUGS language and estimated using OpenBUGS using Bayesian Markov Chain Monte Carlo (MCMC) methods. This entails selecting prior distributions for the unknown model parameters and updating these via the likelihood of the observed choice data. The specification of the priors and the model codes are presented below. All estimations used 10,000 MCMC iterations to let three chains converge from divergent starting points and another 20,000 MCMC iterations to reliably approximate the posterior distributions of the parameters. Convergence was evaluated based on a visual inspection of the MCMC chains and the diagnostics as included in the OpenBUGS software. Custom MCMC slice samplers were used for the GMNL-scale and the rho parameters; these are available from the authors upon request.

Model #1. Separate S-MNL models for each stakeholder group

```
model {

 # N  = number of respondents
 # N1 = number of respondents in P1 group
 # N2 = number of respondents in P2 group
 # N3 = number of respondents in P3 group
 # N4 = number of respondents in P4 group
 # T  = number of choice tasks
 # V  = number of parameters

 # likelihood of pairwise choice tasks
 for (n in 1:N){
   for(t in 1:T){
     Y[n,t] <- 0
     Y[n,t] ~ dcat2(phi[n,t])
 }}

 # utility function P1
 for (n in 1:N1){
   for (t in 1:T){
     phi[n,t]  <- (attr1[n,t] + attr2[n,t] + attr3[n,t] + attr4[n,t] + attr5[n,t] +
                   attr6[n,t] + attr7[n,t] + attr8[n,t]) * gmnl_scale[n]
     attr1[n,t] <- (beta[1,1]  * X[n,t,1]  + beta[1,2]  * X[n,t,2] )
     attr2[n,t] <- (beta[1,3]  * X[n,t,3]  + beta[1,4]  * X[n,t,4] )
     attr3[n,t] <- (beta[1,5]  * X[n,t,5]  + beta[1,6]  * X[n,t,6] )
     attr4[n,t] <- (beta[1,7]  * X[n,t,7]  + beta[1,8]  * X[n,t,8] )
     attr5[n,t] <- (beta[1,9]  * X[n,t,9]  + beta[1,10]  * X[n,t,10])
     attr6[n,t] <- (beta[1,11] * X[n,t,11] + beta[1,12] * X[n,t,12])
     attr7[n,t] <- (beta[1,13] * X[n,t,13] + beta[1,14] * X[n,t,14])
     attr8[n,t] <- (beta[1,15] * X[n,t,15] + beta[1,16] * X[n,t,16])
 }}

 # utility function P2
 for (n in N1+1:N1+N2){
   for (t in 1:T){
     phi[n,t]  <- (attr1[n,t] + attr2[n,t] + attr3[n,t] + attr4[n,t] + attr5[n,t] +
                   attr6[n,t] + attr7[n,t] + attr8[n,t]) * gmnl_scale[n]
     attr1[n,t] <- (beta[2,1] * X[n,t,1]  + beta[2,2] * X[n,t,2] )
     attr2[n,t] <- (beta[2,3] * X[n,t,3]  + beta[2,4] * X[n,t,4] )
     attr3[n,t] <- (beta[2,5] * X[n,t,5]  + beta[2,6] * X[n,t,6] )
     attr4[n,t] <- (beta[2,7] * X[n,t,7]  + beta[2,8] * X[n,t,8] )
     attr5[n,t] <- (beta[2,9] * X[n,t,9]  + beta[2,10]* X[n,t,10])
     attr6[n,t] <- (beta[2,11]* X[n,t,11] + beta[2,12]* X[n,t,12])
     attr7[n,t] <- (beta[2,13]* X[n,t,13] + beta[2,14]* X[n,t,14])
     attr8[n,t] <- (beta[2,15]* X[n,t,15] + beta[2,16]* X[n,t,16])
 }}


 # utility function P3
 for (n in N1+N2+1:N1+N2+N3){
   for (t in 1:T){
     phi[n,t]  <- (attr1[n,t] + attr2[n,t] + attr3[n,t] + attr4[n,t] + attr5[n,t] +
                   attr6[n,t] + attr7[n,t] + attr8[n,t]) * gmnl_scale[n]
     attr1[n,t] <- (beta[3,1] * X[n,t,1]  + beta[3,2] * X[n,t,2] )
     attr2[n,t] <- (beta[3,3] * X[n,t,3]  + beta[3,4] * X[n,t,4] )
     attr3[n,t] <- (beta[3,5] * X[n,t,5]  + beta[3,6] * X[n,t,6] )
     attr4[n,t] <- (beta[3,7] * X[n,t,7]  + beta[3,8] * X[n,t,8] )
     attr5[n,t] <- (beta[3,9] * X[n,t,9]  + beta[3,10]* X[n,t,10])
```


For peer review only - http://bmjopen.bmj.com/site/about/guidelines.xhtml

For peer review only

```
      attr6[n,t] <- (beta[3,11]* X[n,t,11] + beta[3,12]* X[n,t,12])
      attr7[n,t] <- (beta[3,13]* X[n,t,13] + beta[3,14]* X[n,t,14])
      attr8[n,t] <- (beta[3,15]* X[n,t,15] + beta[3,16]* X[n,t,16])
  }}

  # utility function P4
  for (n in N1+N2+N3+1:N1+N2+N3+N4){
    for (t in 1:T){
      phi[n,t]  <- (attr1[n,t] + attr2[n,t] + attr3[n,t] + attr4[n,t] + attr5[n,t] +
                     attr6[n,t] + attr7[n,t] + attr8[n,t]) * gmnl_scale[n]
      attr1[n,t] <- (beta[4,1] * X[n,t,1]  + beta[4,2] * X[n,t,2] )
      attr2[n,t] <- (beta[4,3] * X[n,t,3]  + beta[4,4] * X[n,t,4] )
      attr3[n,t] <- (beta[4,5] * X[n,t,5]  + beta[4,6] * X[n,t,6] )
      attr4[n,t] <- (beta[4,7] * X[n,t,7]  + beta[4,8] * X[n,t,8] )
      attr5[n,t] <- (beta[4,9] * X[n,t,9]  + beta[4,10]* X[n,t,10])
      attr6[n,t] <- (beta[4,11]* X[n,t,11] + beta[4,12]* X[n,t,12])
      attr7[n,t] <- (beta[4,13]* X[n,t,13] + beta[4,14]* X[n,t,14])
      attr8[n,t] <- (beta[4,15]* X[n,t,15] + beta[4,16]* X[n,t,16])
  }}

  # priors on beta's
  beta[1,1:V] ~ dmnorm(zeros[], tau_beta[,])
  beta[2,1:V] ~ dmnorm(zeros[], tau_beta[,])
  beta[3,1:V] ~ dmnorm(zeros[], tau_beta[,])
  beta[4,1:V] ~ dmnorm(zeros[], tau_beta[,])

  for (b in 1:V){
   zeros[b] <- 0
   for (bb in 1:V){
     tau_beta[b,bb] <- equals(b,bb)/100
  }}

  # priors on gmnl_scale parameters
  # subject to a mean-of-one constraint for identification
  gmnl_scale[1:N1] ~ dGMNLscale(tau[1])
  gmnl_scale[N1+1:N1+N2] ~ dGMNLscale(tau[2])
  gmnl_scale[N1+N2+1:N1+N2+N3] ~ dGMNLscale(tau[3])
  gmnl_scale[N1+N2+N3+1:N1+N2+N3+N4] ~ dGMNLscale(tau[4])
  # this implies that SD(tau) is restricted between 0.01 and 10
  for (group in 1:4){
    tau[group] ~ dunif(0.01,10000)
  }

  # monitor SD of gmnl_scale parameters
  for (group in 1:4){
    gmnl_SD[group] <- sqrt( 1.0/ tau[group] )
  }

  # monitor attribute importances
  for (group in 1:4){
    weight[group,1] <- beta[group,2]
    weight[group,2] <- beta[group,4]
    weight[group,3] <- beta[group,6]
    weight[group,4] <- beta[group,8]
    weight[group,5] <- beta[group,10]
    weight[group,6] <- beta[group,12]
    weight[group,7] <- beta[group,14]
    weight[group,8] <- beta[group,16]
    total_weight[group] <- sum(weight[group,])
    attr_importance[group,1] <- weight[group,1] / total_weight[group]
    attr_importance[group,2] <- weight[group,2] / total_weight[group]
    attr_importance[group,3] <- weight[group,3] / total_weight[group]
    attr_importance[group,4] <- weight[group,4] / total_weight[group]
    attr_importance[group,5] <- weight[group,5] / total_weight[group]
    attr_importance[group,6] <- weight[group,6] / total_weight[group]
    attr_importance[group,7] <- weight[group,7] / total_weight[group]
    attr_importance[group,8] <- weight[group,8] / total_weight[group]
  }
}
```


For peer review only

Model #2. Combined S-MNL model with multiplicative attribute-importance parameters

```
model {

 # likelihood of pairwise choice tasks
 for (n in 1:N){
   for(t in 1:T){
     Y[n,t] <- 0
     Y[n,t] ~ dcat2(phi[n,t])
 }}

 # utility function P1
 for (n in 1:N1){
   for (t in 1:T){
     phi[n,t]  <- (attr1[n,t] + attr2[n,t] + attr3[n,t] + attr4[n,t] + attr5[n,t] +
                   attr6[n,t] + attr7[n,t] + attr8[n,t]) * gmnl_scale[n] * scale[1]
     attr1[n,t] <- (beta[1]  * X[n,t,1]  + beta[2]  * X[n,t,2] )
     attr2[n,t] <- (beta[3]  * X[n,t,3]  + beta[4]  * X[n,t,4] )
     attr3[n,t] <- (beta[5]  * X[n,t,5]  + beta[6]  * X[n,t,6] )
     attr4[n,t] <- (beta[7]  * X[n,t,7]  + beta[8]  * X[n,t,8] )
     attr5[n,t] <- (beta[9]  * X[n,t,9]  + beta[10] * X[n,t,10])
     attr6[n,t] <- (beta[11] * X[n,t,11] + beta[12] * X[n,t,12])
     attr7[n,t] <- (beta[13] * X[n,t,13] + beta[14] * X[n,t,14])
     attr8[n,t] <- (beta[15] * X[n,t,15] + beta[16] * X[n,t,16])
 }}

 # utility function P2
 for (n in N1+1:N1+N2){
   for (t in 1:T){
     phi[n,t]  <- (attr1[n,t] + attr2[n,t] + attr3[n,t] + attr4[n,t] + attr5[n,t] +
                   attr6[n,t] + attr7[n,t] + attr8[n,t]) * gmnl_scale[n] * scale[2]
     attr1[n,t] <- (beta[1] * X[n,t,1]  + beta[2] * X[n,t,2] ) * rho[2,1]
     attr2[n,t] <- (beta[3] * X[n,t,3]  + beta[4] * X[n,t,4] ) * rho[2,2]
     attr3[n,t] <- (beta[5] * X[n,t,5]  + beta[6] * X[n,t,6] ) * rho[2,3]
     attr4[n,t] <- (beta[7] * X[n,t,7]  + beta[8] * X[n,t,8] ) * rho[2,4]
     attr5[n,t] <- (beta[9] * X[n,t,9]  + beta[10]* X[n,t,10]) * rho[2,5]
     attr6[n,t] <- (beta[11]* X[n,t,11] + beta[12]* X[n,t,12]) * rho[2,6]
     attr7[n,t] <- (beta[13]* X[n,t,13] + beta[14]* X[n,t,14]) * rho[2,7]
     attr8[n,t] <- (beta[15]* X[n,t,15] + beta[16]* X[n,t,16]) * rho[2,8]
 }}


 # utility function P3
 for (n in N1+N2+1:N1+N2+N3){
   for (t in 1:T){
     phi[n,t]  <- (attr1[n,t] + attr2[n,t] + attr3[n,t] + attr4[n,t] + attr5[n,t] +
                   attr6[n,t] + attr7[n,t] + attr8[n,t]) * gmnl_scale[n] * scale[3]
     attr1[n,t] <- (beta[1] * X[n,t,1]  + beta[2] * X[n,t,2] ) * rho[3,1]
     attr2[n,t] <- (beta[3] * X[n,t,3]  + beta[4] * X[n,t,4] ) * rho[3,2]
     attr3[n,t] <- (beta[5] * X[n,t,5]  + beta[6] * X[n,t,6] ) * rho[3,3]
     attr4[n,t] <- (beta[7] * X[n,t,7]  + beta[8] * X[n,t,8] ) * rho[3,4]
     attr5[n,t] <- (beta[9] * X[n,t,9]  + beta[10]* X[n,t,10]) * rho[3,5]
     attr6[n,t] <- (beta[11]* X[n,t,11] + beta[12]* X[n,t,12]) * rho[3,6]
     attr7[n,t] <- (beta[13]* X[n,t,13] + beta[14]* X[n,t,14]) * rho[3,7]
     attr8[n,t] <- (beta[15]* X[n,t,15] + beta[16]* X[n,t,16]) * rho[3,8]
 }}

 # utility function P4
 for (n in N1+N2+N3+1:N1+N2+N3+N4){
   for (t in 1:T){
     phi[n,t]  <- (attr1[n,t] + attr2[n,t] + attr3[n,t] + attr4[n,t] + attr5[n,t] +
                   attr6[n,t] + attr7[n,t] + attr8[n,t]) * gmnl_scale[n] * scale[4]
     attr1[n,t] <- (beta[1] * X[n,t,1]  + beta[2] * X[n,t,2] ) * rho[4,1]
     attr2[n,t] <- (beta[3] * X[n,t,3]  + beta[4] * X[n,t,4] ) * rho[4,2]
     attr3[n,t] <- (beta[5] * X[n,t,5]  + beta[6] * X[n,t,6] ) * rho[4,3]
     attr4[n,t] <- (beta[7] * X[n,t,7]  + beta[8] * X[n,t,8] ) * rho[4,4]
     attr5[n,t] <- (beta[9] * X[n,t,9]  + beta[10]* X[n,t,10]) * rho[4,5]
     attr6[n,t] <- (beta[11]* X[n,t,11] + beta[12]* X[n,t,12]) * rho[4,6]
     attr7[n,t] <- (beta[13]* X[n,t,13] + beta[14]* X[n,t,14]) * rho[4,7]
     attr8[n,t] <- (beta[15]* X[n,t,15] + beta[16]* X[n,t,16]) * rho[4,8]
 }}

 # priors on beta's
 beta[1:V] ~ dmnorm(zeros[], tau_beta[,])

 for (b in 1:V){
  zeros[b] <- 0
  for (bb in 1:V){
    tau_beta[b,bb] <- equals(b,bb)/100
 }}
```


For peer review only - http://bmjopen.bmj.com/site/about/guidelines.xhtml

```
# priors on gmnl_scale parameters
# subject to a mean-of-one constraint for identification
gmnl_scale[1:N1] ~ dGMNLscale(tau[1])
gmnl_scale[N1+1:N1+N2] ~ dGMNLscale(tau[2])
gmnl_scale[N1+N2+1:N1+N2+N3] ~ dGMNLscale(tau[3])
gmnl_scale[N1+N2+N3+1:N1+N2+N3+N4] ~ dGMNLscale(tau[4])
# this implies that SD(tau) is restricted between 0.01 and 10
for (group in 1:4){
  tau[group] ~ dunif(0.01,10000)
}

# monitor SD of gmnl_scale parameters
for (group in 1:4){
  gmnl_SD[group] <- sqrt( 1.0/ tau[group] )
}

# priors on relative attribute importance parameters (P1 is the base-case sample)
# subject to a mean-of-one constraint for identification
# see Jonker et al. 2019, this prior places 95% of the prior mass between 0.4 and 2.0
 for (att in 1:8){
  rho[1,att] <- 1
}
for (group in 2:4){
  rho[group,1:8] ~ dscale(6.25)
}

# priors on the relative scale parameters (P1 is the base-case sample)
# see Jonker et al. 2019, this prior places 95% of the prior mass between 0.4 and 2.0
scale[1] <- 1
for (group in 2:4) {
  scale[group] ~ dlnorm(-0.08,6.25)C(0.1,10)
}
# the truncation normalizing constant in the log prior does not need to be explicitly
# evaluated since the truncation boundaries remain constant during MCMC sampling. Hence
# sensoring C() is sufficient and can be used instead of truncation T(), which isn't implemented


# monitor attribute importances
for (group in 1:4){
  weight[group,1] <- beta[2]  * rho[group,1]
  weight[group,2] <- beta[4]  * rho[group,2]
  weight[group,3] <- beta[6]  * rho[group,3]
  weight[group,4] <- beta[8]  * rho[group,4]
  weight[group,5] <- beta[10] * rho[group,5]
  weight[group,6] <- beta[12] * rho[group,6]
  weight[group,7] <- beta[14] * rho[group,7]
  weight[group,8] <- beta[16] * rho[group,8]
  total_weight[group] <- sum(weight[group,])
  attr_importance[group,1] <- weight[group,1] / total_weight[group]
  attr_importance[group,2] <- weight[group,2] / total_weight[group]
  attr_importance[group,3] <- weight[group,3] / total_weight[group]
  attr_importance[group,4] <- weight[group,4] / total_weight[group]
  attr_importance[group,5] <- weight[group,5] / total_weight[group]
  attr_importance[group,6] <- weight[group,6] / total_weight[group]
  attr_importance[group,7] <- weight[group,7] / total_weight[group]
  attr_importance[group,8] <- weight[group,8] / total_weight[group]
 }
}
```


For peer review only - http://bmjopen.bmj.com/site/about/guidelines.xhtml

Box S3. Indicators of questionnaire completion and perceived difficulty

**Completion**	**AT**	**HR**	**HU**	**DE**	**NL**	**NO**	**ES**	**UK**
Gave informed consent	253	229	283	207	207	203	150	208
Finished all DCE scenario's (%)	170 (67)	173 (76)	194 (93)	166 (80)	160 (77)	158 (78)	150 (100)	165 (79)
Finished entire questionnaire	169 (68)	168 (73)	192 (68)	160 (77)	156 (75)	156 (77)	150 (100)	163 (78)
Mean (SD) time to completion entire questionnaire	22.87 (21.73)	22.43 (37.68)	20.27 (14.88)	26.21 (48.74)	26.21 (48.74)	27.33 (27.90)	26.96 (10.12)	24.68 (62.64)
Required >5 minutes (%)*	167 (99)	160 (95)	180 (94)	158 (99)	156 (100)	152 (97)	149 (99)	155 (95)
**Self-reported difficulty**								
Very easy	16 (9.5)	11 (6.5)	23 (12.0)	14 (8.8)	3 (1.9)	2 (1.3)	1 (0.7)	31 (19.0)
Easy	39 (23.1)	30 (17.9)	71 (37.0)	33 (20.6)	40 (25.6)	20 (12.8)	36 (24.0)	45 (27.6)
Not too easy, not too difficult	85 (50.3)	70 (41.7)	75 (39.1)	84 (52.5)	74 (47.4)	79 (50.6)	71 (47.3)	63 (38.7)
Difficult	25 (14.8)	44 (26.2)	22 (11.5)	24 (15.0)	31 (19.9)	48 (30.8)	30 (20.0)	22 (13.5)
Very difficult	4 (2.4)	13 (7.7)	1 (0.5)	5 (3.1)	8 (5.1)	7 (4.5)	12 (8.0)	2 (1.2)

* As % of those that completed the entire questionnaire

For peer review only

For peer review only - http://bmjopen.bmj.com/site/about/guidelines.xhtml

Box S4. Patients' self-reported health problems.

N (%)	AT	HR	HU	DE	NL	NO	ES	UK
I prefer not to answer	4 (2.4)	10 (6.0)	3 (1.6)	4 (1.9)	2 (1.3)	0 (0)	6 (4)	3 (1.8)
Asthma	20 (11.8)	19 (11.3)	26 (13.5)	18 (8.7)	19 (12.2)	29 (18.6)	6 (4)	41 (25.2)
Cancer during the past five years	10 (5.9)	5 (3.0)	11 (5.7)	4 (1.9)	10 (6.4)	11 (7.1)	18 (12)	10 (6.1)
Chronic back pain or sciatica	69 (40.8)	54 (32.1)	32 (16.7)	47 (22.6)	32 (20.5)	29 (18.6)	50 (33.3)	51 (31.3)
Chronic bronchitis, COPD or emphysema	32 (18.9)	8 (4.8)	9 (4.7)	14 (6.7)	26 (16.7)	7 (4.5)	29 (19.3)	28 (17.2)
Chronic kidney disease	4 (2.4)	6 (3.6)	8 (4.2)	9 (4.3)	4 (2.6)	0 (0)	9 (6)	8 (4.9)
Cognitive problems, memory loss, dementia, Alzheimer's	3 (1.8)	0 (0)	2 (1.0)	2 (1.0)	6 (3.8)	6 (3.9)	0 (0)	4 (2.5)
Colon problem, irritable bowel or colitis	28 (16.6)	18 (10.7)	15 (7.8)	9 (4.3)	34 (21.8)	31 (19.9)	7 (4.7)	32 (19.6)
Congestive heart failure	9 (5.3)	1 (0.6)	4 (2.2)	8 (3.8)	9 (5.8)	5 (3.2)	9 (6)	3 (1.8)
Depression, anxiety or emotional difficulties	63 (37.3)	47 (28.0)	63 (32.8)	36 (17.3)	29 (18.6)	32 (20.5)	36 (24)	70 (42.9)
Diabetes	29 (17.2)	17 (10.1)	43 (22.4)	35 (16.8)	36 (23.1)	16 (10.3)	26 (17.3)	45 (27.6)
Hard of hearing	18 (10.7)	16 (9.5)	27 (14.1)	14 (6.7)	28 (17.9)	35 (22.4)	17 (11.3)	18 (11.0)
Heart disease, angina (chest pain from heart problem), heart attack, bypass surgery or angioplasty	20 (11.8)	19 (11.3)	20 (10.4)	10 (4.8)	26 (16.7)	15 (9.6)	19 (12.7)	28 (17.2)
High blood pressure	67 (39.6)	40 (23.8)	95 (49.5)	68 (32.7)	61 (39.1)	58 (37.2)	50 (33.3)	64 (39.3)
High cholesterol	34 (20.1)	27 (16.1)	46 (24.0)	40 (19.2)	47 (30.1)	38 (24.4)	45 (30)	47 (28.8)
Osteoarthritis (not rheumatoid arthritis)	5 (3.0)	9 (5.4)	59 (30.7)	7 (3.4)	33 (21.2)	46 (29.5)	7 (4.7)	43 (26.4)
Osteoporosis (brittle bones)	15 (8.9)	11 (6.5)	13 (6.8)	6 (2.9)	10 (6.4)	9 (5.8)	21 (14)	16 (9.8)
Overweight	55 (32.5)	46 (27.4)	68 (35.4)	56 (26.9)	49 (31.4)	51 (32.7)	47 (31.3)	47 (28.8)
Poor circulation in your legs	22 (13.0)	27 (16.1)	49 (25.5)	17 (8.2)	21 (13.5)	22 (14.1)	35 (23.3)	23 (14.1)
Rheumatoid arthritis	25 (14.8)	10 (6.0)	19 (9.9)	17 (8.2)	21 (13.5)	10 (6.4)	12 (8)	19 (11.7)

For peer review only - http://bmjopen.bmj.com/site/about/guidelines.xhtml

Rheumatic disease, fibromyalgia or lupus	14 (8.3)	5 (3.0)	13 (6.8)	15 (7.2)	11 (7.1)	16 (10.3)	12 (8)	11 (6.7)
Stomach problem, ulcer, gastritis or reflux	29 (17.2)	27 (16.1)	46 (24.0)	25 (12.0)	21 (13.5)	24 (15.4)	22 (14.7)	28 (17.2)
Stroke	4 (2.4)	4 (2.4)	8 (4.2)	10 (4.8)	3 (1.9)	2 (1.3)	7 (4.7)	9 (5.5)
Thyroid disorder	30 (17.8)	33 (19.6)	23 (12.0)	27 (13.0)	14 (9.0)	18 (11.5)	10 (6.7)	16 (9.8)
Vision problem	26 (15.4)	50 (29.8)	28 (14.6)	29 (13.9)	28 (17.9)	21 (13.5)	56 (37.3)	18 (11.0)
No health problems	1 (0.6)	6 (3.6)	0 (0.0)	28 (13.5)	2 (1.3)	0 (0)	3 (2)	0 (0)
Other health problems	39 (23.1)	30 (17.9)	31 (16.2)	16 (7.7)	39 (25.0)	34 (21.8)	31 (20.7)	73 (44.8)

For peer review only

Supplementary file Box S5. Coefficients and 95% credibility intervals (CI) of the multiplicative interaction terms to investigate differences between patients (P1) and the other stakeholder groups, i.e., Partners & other informal caregivers (P2), professionals (P3), payers (P4), and policy makers (P5). The bold cells indicate the 95% CI of the multiplicative interaction terms for stakeholder group and attribute that excluded 1.

		**P2 vs P1**			**P3 vs P1**			**P4/5 vs P1**			**P4 vs P1**			**P5 vs P1**		
		**coefficient**	**95% CI**		**coefficient**	**95% CI**		**coefficient**	**95% CI**		**coefficient**	**95% CI**		**coefficient**	**95% CI**	
**AT**	Physical functioning	0.74	0.52	**1.00**	0.64	0.48	**0.82**	0.65	0.48	**0.87**						
	Psych.l well-being	1.25	0.95	1.62	1.14	0.91	1.42	1.06	0.82	1.35						
	Social relations & part	0.93	0.62	1.29	1.16	0.92	1.45	1.09	0.83	1.40						
	Enjoyment of life	1.11	0.88	1.38	0.94	0.78	1.11	0.89	0.72	1.09						
	Resilience	0.98	0.74	1.24	0.87	0.70	1.07	0.79	0.61	1.00						
	Person-centeredness	1.27	0.79	1.83	1.45	**1.07**	1.88	1.16	0.79	1.58						
	Continuity of care	1.01	0.76	1.30	1.17	0.94	1.41	1.07	0.83	1.34						
	Total costs	0.71	0.40	1.10	0.64	0.39	**0.97**	1.29	0.87	1.83						
**DE**	Physical functioning	0.56	0.34	**0.83**	0.52	0.38	**0.69**	0.77	0.57	1.00						
	Psychological well-being	0.81	0.50	1.22	1.04	0.77	1.40	1.19	0.88	1.58						
	Social relations & part	0.98	0.63	1.40	1.01	0.77	1.31	0.88	0.64	1.16						
	Enjoyment of life	1.07	0.84	1.35	0.80	0.66	**0.96**	0.78	0.63	**0.96**						
	Resilience	1.00	0.74	1.29	0.78	0.63	**0.96**	0.73	0.57	**0.91**						
	Person-centeredness	1.09	0.66	1.64	1.70	**1.28**	2.17	1.17	0.82	1.59						
	Continuity of care	1.32	1.00	1.69	1.19	0.98	1.44	0.89	0.69	1.12						
	Total costs	1.16	0.66	1.72	0.95	0.65	1.36	1.59	**1.15**	2.15						
**ES**	Physical functioning	1.23	**1.06**	1.44	0.51	0.41	**0.64**									
	Psychological well-being	0.77	0.64	**0.91**	0.73	0.60	**0.87**									
	Social relations & part	1.01	0.84	1.20	0.73	0.60	**0.88**									
	Enjoyment of life	0.87	0.72	1.03	0.96	0.80	1.13									
	Resilience	1.01	0.83	1.22	0.80	0.65	**0.99**									
	Person-centeredness	1.43	**1.16**	1.75	1.64	**1.35**	1.96									
	Continuity of care	0.95	0.70	1.25	1.12	0.86	1.45									
	Total costs	0.73	0.43	1.11	1.50	**1.06**	2.09									

For peer review only

For peer review only - http://bmjopen.bmj.com/site/about/guidelines.xhtml

**HR**	Physical functioning	0.87	0.62	1.16	0.86	0.59	1.14	1.20	0.89	1.55						
	Psychological well-being	1.04	0.83	1.30	1.07	0.84	1.33	0.83	0.62	1.06						
	Social relations & part	0.92	0.70	1.17	1.00	0.77	1.26	0.91	0.67	1.17						
	Enjoyment of life	1.01	0.83	1.21	1.22	**1.02**	1.45	1.01	0.82	1.23						
	Resilience	0.81	0.63	1.01	0.81	0.63	1.01	1.03	0.81	1.29						
	Person-centeredness	1.14	0.91	1.39	1.08	0.86	1.33	0.95	0.73	1.19						
	Continuity of care	1.20	0.98	1.44	1.26	**1.03**	1.51	0.87	0.68	1.10						
	Total costs	1.00	0.55	1.59	0.70	0.36	1.18	1.20	0.67	1.91						
**HU**	Physical functioning	0.77	0.53	1.06	0.89	0.67	1.16	0.87	0.67	1.10						
	Psychological well-being	1.57	**1.14**	2.08	1.16	0.84	1.55	1.16	0.87	1.52						
	Social relations & part	1.01	0.70	1.35	0.93	0.69	1.22	1.05	0.82	1.32						
	Enjoyment of life	1.30	0.98	1.66	1.14	0.89	1.42	1.04	0.84	1.29						
	Resilience	0.80	0.57	1.08	0.77	0.58	1.01	0.65	0.50	**0.84**						
	Person-centeredness	0.90	0.64	1.19	1.32	**1.04**	1.66	1.01	0.79	1.25						
	Continuity of care	1.05	0.83	1.30	0.95	0.77	1.15	0.81	0.66	**0.97**						
	Total costs	0.60	0.29	1.07	0.83	0.44	1.32	1.42	0.91	2.09						
**NL**	Physical functioning	0.77	0.63	**0.92**	0.73	0.61	**0.87**				0.92	0.79	1.06	0.87	0.74	1.00
	Psychological well-being	0.91	0.74	1.12	1.10	0.93	1.32				1.12	0.94	1.34	0.91	0.77	1.09
	Social relations & part	0.97	0.76	1.22	1.15	0.96	1.38				1.09	0.90	1.32	1.09	0.91	1.30
	Enjoyment of life	1.11	0.96	1.27	0.99	0.87	1.13				1.05	0.92	1.20	0.96	0.85	1.09
	Resilience	0.95	0.81	1.11	0.83	0.71	**0.96**				0.77	0.65	**0.89**	0.93	0.81	1.06
	Person-centeredness	1.05	0.81	1.33	1.02	0.82	1.26				0.80	0.61	1.01	0.98	0.78	1.21
	Continuity of care	1.07	0.85	1.31	0.91	0.72	1.10				0.73	0.56	**0.91**	0.82	0.66	1.00
	Total costs	1.16	0.81	1.55	1.27	0.96	1.62				1.53	**1.19**	1.93	1.43	**1.12**	1.82
**NO**	Physical functioning	0.66	0.53	**0.80**	0.71	0.60	**0.83**				0.77	0.64	**0.90**	0.63	0.53	**0.75**
	Psychological well-being	1.08	0.90	1.27	0.98	0.84	1.14				0.87	0.72	1.03	0.86	0.73	1.01

For peer review only - http://bmjopen.bmj.com/site/about/guidelines.xhtml

	Social relations & part	1.32	**1.09**	1.56	1.19	**1.01**	1.40				0.97	0.79	1.17	1.18	0.98	1.39
	Enjoyment of life	1.12	0.94	1.32	1.11	0.94	1.29				1.04	0.87	1.23	0.99	0.84	1.15
	Resilience	0.83	0.64	1.06	1.00	0.84	1.19				1.09	0.90	1.32	1.07	0.88	1.29
	Person-centeredness	1.25	0.86	1.66	1.08	0.79	1.41				0.79	0.51	1.09	1.10	0.81	1.44
	Continuity of care	0.86	0.67	1.09	0.92	0.74	1.12				0.72	0.57	**0.92**	1.03	0.84	1.26
	Total costs	0.88	0.46	1.41	1.02	0.62	1.48				1.77	**1.25**	2.36	1.13	0.71	1.66
**UK**	Physical functioning	0.82	0.63	1.03	0.94	0.77	1.14	0.94	0.76	1.15						
	Psychological well-being	1.17	0.95	1.42	1.00	0.82	1.20	1.07	0.85	1.31						
	Social relations & part	1.06	0.84	1.29	1.03	0.83	1.25	1.01	0.79	1.25						
	Enjoyment of life	1.15	0.97	1.34	0.98	0.83	1.14	0.94	0.79	1.12						
	Resilience	1.01	0.79	1.26	0.97	0.78	1.17	1.04	0.83	1.29						
	Person-centeredness	1.06	0.79	1.34	1.11	0.87	1.39	1.18	0.90	1.50						
	Continuity of care	0.85	0.65	1.09	0.87	0.68	1.09	0.99	0.77	1.24						
	Total costs	0.89	0.57	1.24	1.11	0.81	1.48	0.82	0.52	1.17						

For peer review only

For peer review only

For peer review only - http://bmjopen.bmj.com/site/about/guidelines.xhtml
